# Emerging biological archives can reveal ecological and climatic change in Antarctica

**DOI:** 10.1111/gcb.16356

**Published:** 2022-09-08

**Authors:** Jan M. Strugnell, Helen V. McGregor, Nerida G. Wilson, Karina T. Meredith, Steven L. Chown, Sally C. Y. Lau, Sharon A. Robinson, Krystyna M. Saunders

**Affiliations:** ^1^ Centre for Sustainable Tropical Fisheries and Aquaculture and College of Science and Engineering James Cook University Townsville Queensland Australia; ^2^ Securing Antarctica's Environmental Future James Cook University Townsville Queensland Australia; ^3^ Securing Antarctica's Environmental Future, School of Earth, Atmospheric and Life Sciences University of Wollongong Wollongong New South Wales Australia; ^4^ Securing Antarctica's Environmental Future Western Australian Museum Western Australia Australia; ^5^ Research and Collections Western Australian Museum Western Australia Australia; ^6^ School of Biological Sciences University of Western Australia Crawley Western Australia Australia; ^7^ Securing Antarctica's Environmental Future Australian Nuclear Science and Technology Organisation Lucas Heights New South Wales Australia; ^8^ Securing Antarctica's Environmental Future, School of Biological Sciences Monash University Melbourne Victoria Australia; ^9^ Institute for Marine and Antarctic Studies University of Tasmania Hobart Tasmania Australia

**Keywords:** benthos, coalescent inference, lake sediments, mosses, paleoecology, peat, sclerochronology, Southern Ocean, stable isotopes, terrestrial invertebrate

## Abstract

Anthropogenic climate change is causing observable changes in Antarctica and the Southern Ocean including increased air and ocean temperatures, glacial melt leading to sea‐level rise and a reduction in salinity, and changes to freshwater water availability on land. These changes impact local Antarctic ecosystems and the Earth's climate system. The Antarctic has experienced significant past environmental change, including cycles of glaciation over the Quaternary Period (the past ~2.6 million years). Understanding Antarctica's paleoecosystems, and the corresponding paleoenvironments and climates that have shaped them, provides insight into present day ecosystem change, and importantly, helps constrain model projections of future change. Biological archives such as extant moss beds and peat profiles, biological proxies in lake and marine sediments, vertebrate animal colonies, and extant terrestrial and benthic marine invertebrates, complement other Antarctic paleoclimate archives by recording the nature and rate of past ecological change, the paleoenvironmental drivers of that change, and constrain current ecosystem and climate models. These archives provide invaluable information about terrestrial ice‐free areas, a key location for Antarctic biodiversity, and the continental margin which is important for understanding ice sheet dynamics. Recent significant advances in analytical techniques (e.g., genomics, biogeochemical analyses) have led to new applications and greater power in elucidating the environmental records contained within biological archives. Paleoecological and paleoclimate discoveries derived from biological archives, and integration with existing data from other paleoclimate data sources, will significantly expand our understanding of past, present, and future ecological change, alongside climate change, in a unique, globally significant region.

## Graphical abstract

Antarctic and Southern Ocean biological archives such as extant moss beds and peat profiles, biological proxies in lake and marine sediments, vertebrate animal colonies, and extant terrestrial and benthic marine invertebrates, record the nature and rate of past ecological change, paleoenvironmental drivers of that change, and constrain ecosystem and climate models. Recent significant advances have led to new applications and greater power in elucidating the environmental records contained within biological archives. Paleoecological and paleoclimate discoveries derived from biological archives will significantly expand our understanding of past, present and future ecological change, alongside climate change, in a unique, globally significant region.

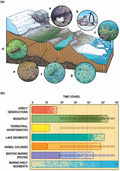

## 1 INTRODUCTION

Antarctica and the Southern Ocean are key drivers of the Earth's atmospheric and oceanic systems and are of fundamental importance to global climate change. Increased ice sheet instability and consequently, global sea level rise, together with changes in the Antarctic Circumpolar Current (ACC), which largely drives global ocean overturning circulation and maintains low temperatures in Antarctica (Rintoul, 2018), threatens the livelihoods of billions of people and global biodiversity (Pörtner et al., 2022). At a more regional scale, Antarctica and its surrounding sub‐Antarctic islands have unique ecosystems under increasing pressure from global climate change, including local changes in response to ice sheet variability, glacier retreat and sea ice extent (Constable et al., 2022). As the substantial risks currently facing these ecosystems are becoming clear (Chown et al., 2022; Lee et al., 2022), research questions are increasingly focusing on understanding the ecosystem impacts of climate change in the Antarctic region. Terrestrial and marine biological archives can reveal the paleoecology of a species or ecosystem, which shows their responses to environmental and climate change. This can help shape predictions about how species may respond to future change and allows comparison of trends across species and regions to identify the most important environmental change factors (Younger et al., 2016). A new generation of biological archives and associated proxies are now emerging.

We define biological archives as of biological origin, including preserved historical biological remains and records within Antarctic organisms alive today, that reveal the Antarctic region's environmental history and/or the paleoecology of that species or ecosystem, including changes in biodiversity, demography and distribution, which together shed light on their responses to environmental and climate change, and can be used to constrain models for the future.

**FIGURE 1:**
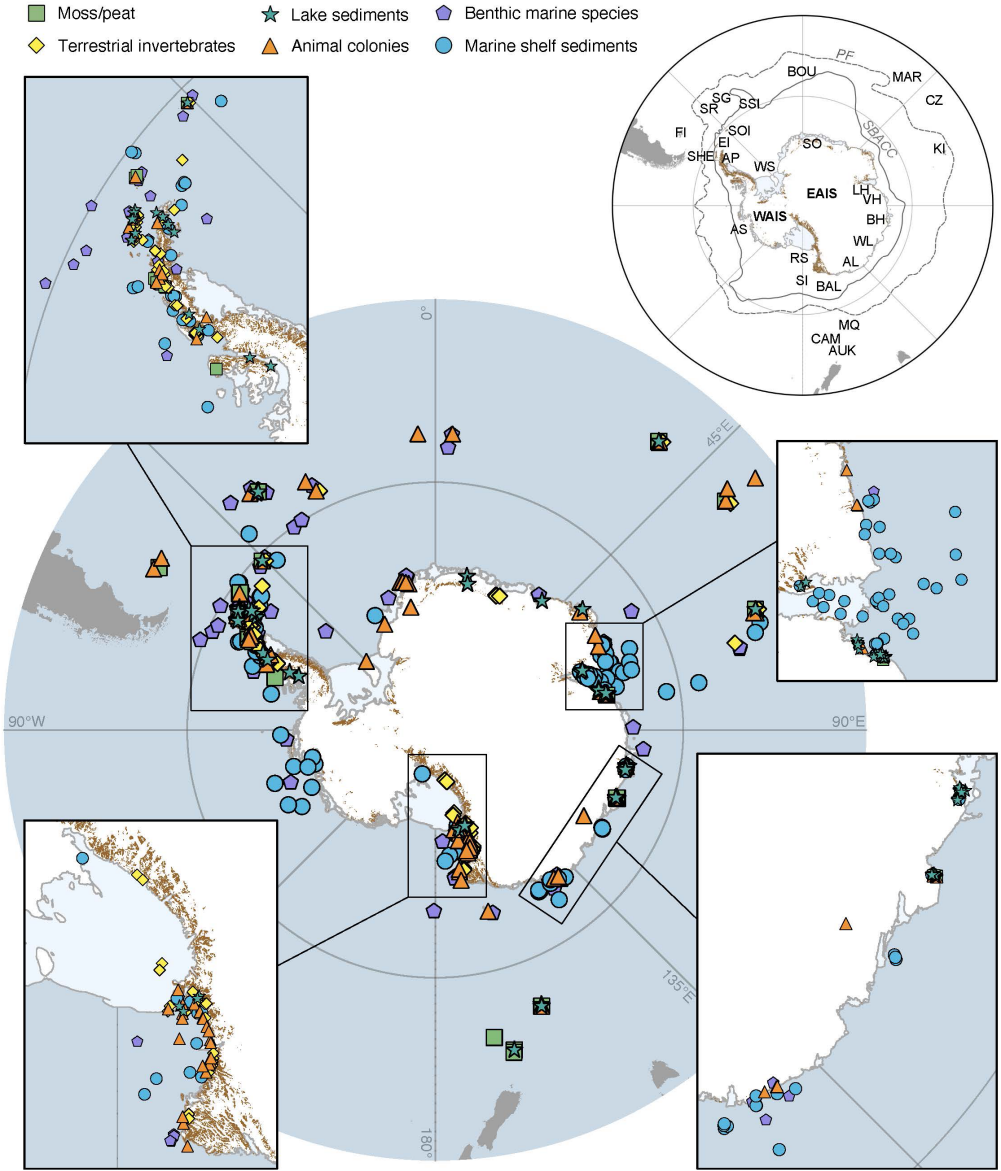
Map of Antarctica and the Southern Ocean showing the locations of studies investigating biological archives with a Quaternary focus. Ice‐free areas (brown areas on the Antarctic continent) indicate the Antarctic Conservation Biogeographic Regions (ACBRs) in (Terauds & Lee, 2016) and are important locations for moss beds, terrestrial invertebrates, and many animal colonies. Currents: PF, Polar Front; SBACC, Southern Boundary of the Antarctic Circumpolar Current. Ice Sheet: EAIS, East Antarctic Ice Sheet; WAIS, West Antarctic Ice Sheet. Localities: AL, Adélie Land; AP, Antarctic Peninsula; AS, Amundsen Sea; AUK, Auckland Is.; BAL, Balleny Is.; BH, Bunger Hills; BOU, Bouvet Is.; CAM, Campbell Is.; CZ, Crozet Is.; EI, Elephant Is.; FI, Falkland Is.; KI, Kerguelen Is.; LH, Larsemann Hills; MAR, Marion Is.; MQ, Macquarie Is.; RS, Ross Sea; SG, South Georgia; SHE, South Shetland Is.; SI, Scott Is.; SOI, South Orkney Is.; SR, Shag Rocks; SSI, South Sandwich Is.; VH, Vestfold Hills; WL, Wilkes Land; WS, Weddell Sea. For references of each datapoint, see Figure S1 and Tables S1–S6.

Some biological proxy records are derived from ‘traditional’ archives, such as moss and peat records, lake and marine sediments, and animal colonies, with new possibilities emerging due to advances in technology and the ability to interrogate records with multiple analytical techniques. Other biological archives are novel and unique to the Antarctic region. Records from these biological archives complement ice and marine sediment core paleoclimate records, because they fill knowledge gaps on aspects of Antarctic climate, ecosystems and environments, which cannot be otherwise obtained. Biota in the Antarctic terrestrial and lacustrine environments are largely limited to the ice‐free areas comprising 0.18%, or 21,745 km^2^ of the Antarctic continent (Burton‐Johnson et al., 2016), where ice cores are unavailable (Convey et al., 2008) (Figure 1). These ice‐free areas provide a substrate for groups such as mosses, lichens, algae, invertebrates, fungi, and microbes (Convey et al., 2008; Lee et al., 2022), from the tops of nunataks protruding through the ice to the many coastal oases (Wauchope et al., 2019). Ice‐free areas are also important breeding grounds for several species of birds, including some penguins, petrels, and seals. In marine environments, a diverse, unique, benthic‐dominated fauna exists on the continental margin and adjacent deep sea, and importantly, in difficult to reach ice shelf regions (Clarke, 2008).

Here, we review the utility of emerging and novel Antarctic biological archives and highlight the analytical techniques being used to investigate them. We discuss their use to date, and document their strengths and weaknesses. We propose next steps to further develop the approaches, including integrating them with existing datasets, and thus present a broader picture of Antarctic climate, ecosystem and environmental change and variability.

## 2 EMERGING BIOLOGICAL ARCHIVES AND ASSOCIATED PROXIES

**FIGURE 2:**
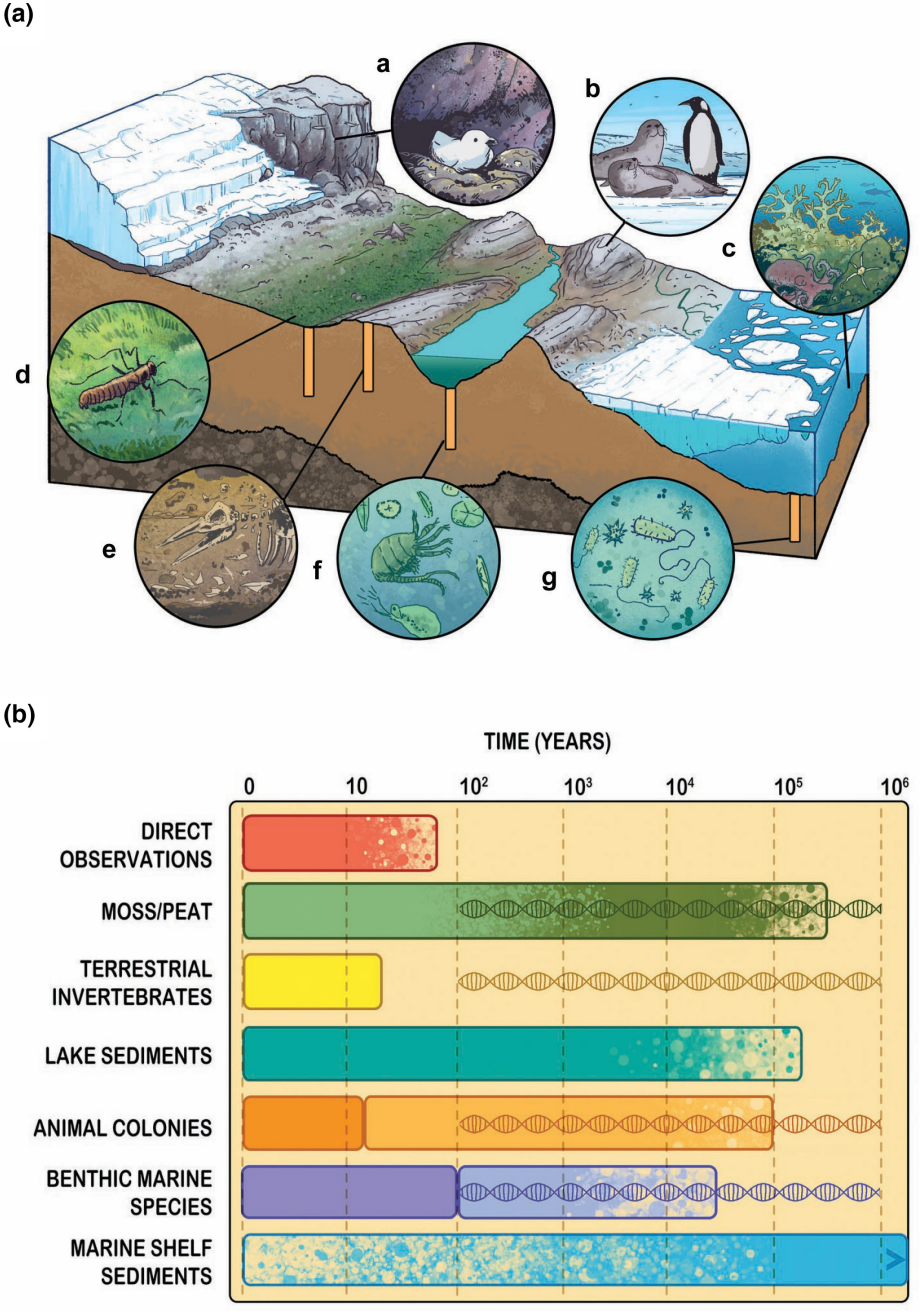
Landscape location and timespan of the Antarctic and Southern Ocean biological archives. (A) Antarctic and Southern Ocean biological archives in situ. (a) snow petrel breeding colony; (b) extant seal and penguin colonies; (c) extant marine benthos, such as brittle stars, octopus, and corals; (d) moss beds, which with time, become peat, and their associated invertebrate communities, here the Antarctic midge, *Belgica antarctica*; (e) historic preserved colonies of seals and penguins, which can contain bones, guano, feathers, hairs, eggs shells; and (f) lake sediments that contain preserved traces of copepods (e.g., *Boeckella poppei* generally mandibles and spermatophores), cladocerans (e.g., *Daphniopsos studeri*) and diatoms; and, (g) marine shelf sediments (e.g., pelagic Archaea), here shown beneath an ice shelf. (B) Indicative time span of the utility of biological archives compared to direct instrumental observations (red), which commenced in the 1950s. Live moss (light green) can be frozen under ice for 1,530 years, be revived and continue to grow (Roads et al., 2014). Partially decayed moss and organic matter and their transformation to peat over time (dark green). Live terrestrial invertebrates (yellow) (tardigrades can reproduce after being frozen for 30.5 years) (Tsujimoto et al., 2016). Lake sediment core (aqua) records date back to 300,000 years (Hendy, 2000). Live colonial animals (dark orange) (snow petrels, seals and penguins live for ~25 years or less), historic preserved animal colonies (light orange) are known from ~100,000 years ago. Benthic marine species (dark purple), (some Southern Ocean scleractinian coral species likely live for ~100 years (Roman Gonzalez, 2021)). Fossilised benthic marine species (light purple), (fossil coral can be used for sclerochronological studies over 10,000s of years (Wilson et al., 2020)). Marine shelf sediment core (blue) records have received greater attention over deep time scales. Erosion of shelf sediments during ice advance limits their utility in obtaining continuous records back past the last glaciation. DNA symbol indicates the use of genetic data from modern moss, terrestrial invertebrates, animal colonies, and benthic marine species to investigate past population size using coalescent methods over a timeframe from ~100 – 1,000,000 years. Illustrator: Daniel A. Becker.

The biological archives and proxies we review largely cover some or all of the Quaternary Period (~2.6 millions of years ago (Ma) to today; Figure 2). During this time the Antarctic was subject to multiple cycles of glaciation, and these have played a strong role in shaping its modern ecosystems. At times of glacial maxima, such as the Last Glacial Maximum (LGM), 26‐19.5 thousand years ago (ka), grounded Antarctic ice sheets expanded out over much of the continental shelf and many sub‐Antarctic islands (Hodgson et al., 2014). This reduced available habitat for terrestrial (Convey et al., 2008) and benthic marine (Thatje et al., 2005) organisms. Oceanographic fronts are understood to have shifted northwards during glacial maxima (Gersonde et al., 2005). Perennial Antarctic sea ice also expanded around the continent and sub‐Antarctic islands (Huybrechts, 2002), and although primary productivity was reduced overall (Hillenbrand & Cortese, 2006), polynyas (ice‐free open water areas) were likely key areas of productivity (Thatje et al., 2008). Conversely, during interglacial periods, such as the Last Interglacial (LIG, ~130–116 ka), ice sheets retreated, leading to greater availability of ice‐free habitats both in marine and terrestrial environments. During these periods only seasonal sea ice coverage of the continental margin would have occurred (Thatje et al., 2005). During the LIG global sea levels were 5–10 m higher than present and mean global air temperatures were 1.5 ± −0.5°C higher than the pre‐industrial period (Fox‐Kemper, 2021).

Antarctic and sub‐Antarctic organisms are very sensitive to their surrounding environment and climate, and therefore they can integrate paleoenvironment and paleoclimate information into their structure throughout their life including, but not limited to, precipitation, salinity, sea ice cover extent, temperature, wind patterns, and strength (Gornitz, 2009). The cold Antarctic environment is well‐suited to preserving historical life in lakes, terrestrial ice‐free environments and their associated communities, including abandoned ancient penguin, petrel, and seal colonies, whereas modern organisms present in these environments contain biological proxies in the form of growth rings or increments (e.g., marine molluscs, scleractinian, and stylasterid corals). Furthermore, the genomes of living organisms contain archives of past demographic change of populations (Donnelly & Tavaré, 1995; Griffiths & Tavaré, 1994), which can be strongly influenced by environmental change and climate (de Bruyn et al., 2011).

Common to all proxies used to reconstruct past climate, environment or ecology is the need to establish a relationship between the proxy and the parameter(s) of interest (Ruddiman, 2013). The relationship is preferably quantitative, and usually empirically derived, but this is not always possible due to multiple factors contributing to the signal, or the absence of a modern equivalent that can be used to establish a relationship. All proxies, including biological proxies, used to reconstruct past climate, ecological, or environmental parameters have a range of common sources of uncertainty (Evans et al., 2013). These comprise random and systematic errors, including analytical uncertainties, uncertainties arising from changes in the preservation of the proxy back in time, assumptions about the relationship between the proxy and the parameter of interest, such as whether the relationship is linear or nonlinear, direct or indirect, assumptions about the stationarity of the relationship back in time, and whether there is more than one parameter affecting a given proxy (Evans et al., 2013). Local context is also important. Common to all reconstructions of the past, and another major source of uncertainty, is a need to estimate the age of the proxy material, again this is preferably a quantitative estimate (‘absolute’ age) where the uncertainties are known. Antarctic biological proxies are subject to the same broad assumptions and uncertainties described here, and in addition, each have their own specific strengths and limitations as discussed below.

## 3 LIVE MOSSES AND PEATS

**FIGURE 3:**
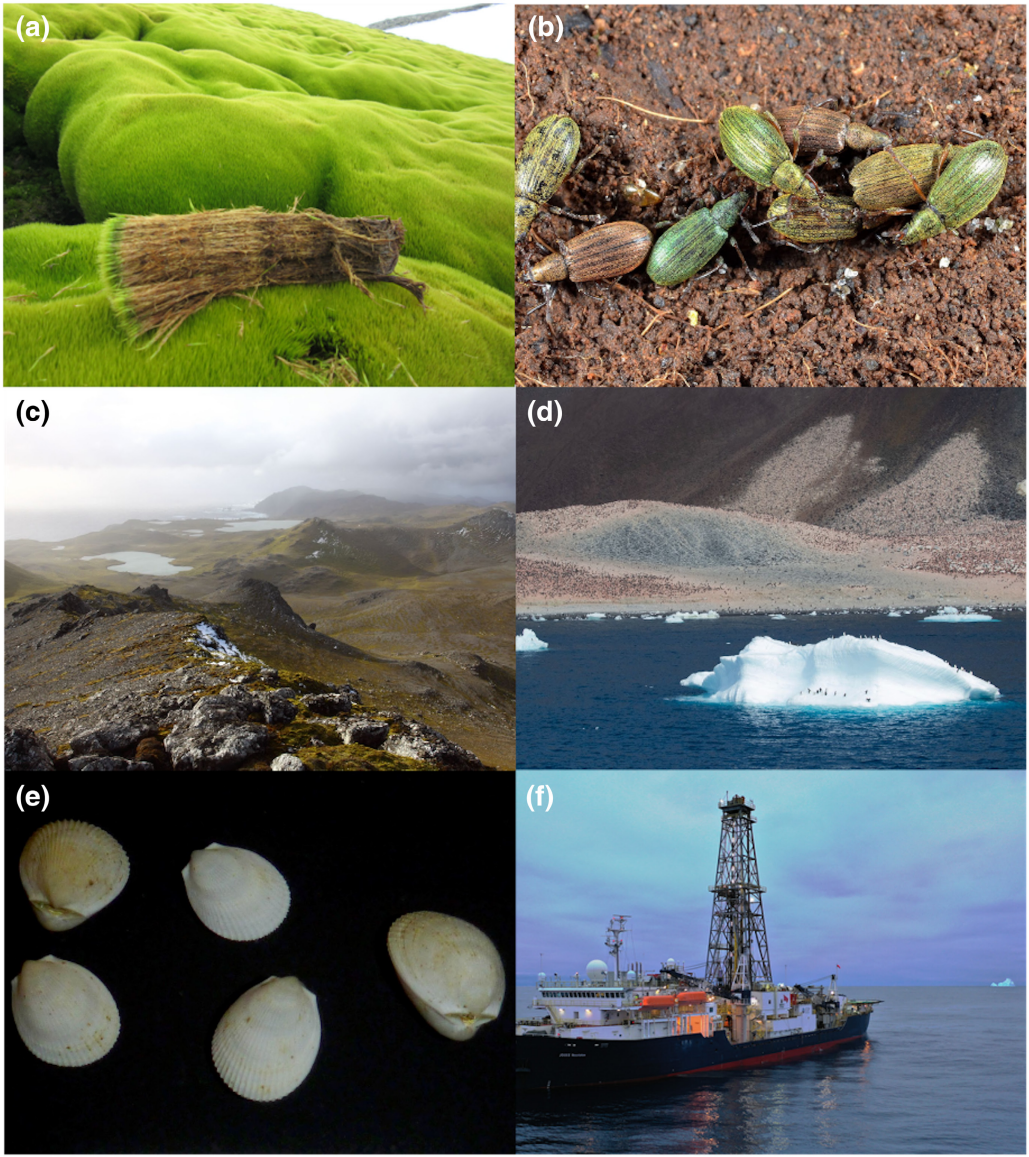
Images of Antarctic and Southern Ocean biological archives. (a) Moss samples from a moss bed on Byers Peninsula, Livingston Island (photo credit—Melinda Waterman), (b) sub‐Antarctic weevil, *Ectemnorhinus viridis*, a terrestrial invertebrate from Kerguelen Island (photo credit—Bernard Chaubert), (c) Sub‐Antarctic lakes on Macquarie Island (photo credit—Krystyna Saunders), (d) Adélie penguin colony, Paulet Island (photo credit—Steven Chown), (e) *Limatula* sp., a benthic marine invertebrate, (photo credit—Nerida Wilson/Greg Rouse), (f) R/V *JOIDES Resolution* on an ocean drilling expedition as part of the IODP (International Ocean Discovery Program) in Amundsen Sea (photo credit—Gohl et al., 2017).

Mosses are the dominant plants found in most ice‐free areas of Antarctica and are present in a range of growth forms, from components of biological soil crusts and small buttons and turfs on the continent, to moss banks and the living tops of peat archives on the Peninsula (Figures 1, 2, and 3). Buried peat sequences and waterlogged ‘peatlands’, which are saturated peat‐forming ecosystems with persistent near‐surface water tables, are not as common as moss banks in Antarctica (Loisel et al., 2017); however, they represent the main type of peat accumulation in the sub‐Antarctic (Bergstrom et al., 2002).

Preserved for long periods of time due to the freezing temperatures, these archives span from the present day, to samples collected by past explorers and preserved in herbaria around the world, to the basal layers of peat made up of preserved moss and other organic matter. The ability of moss and peat records to preserve a record of the environment in which they were growing opens the possibility of understanding how climate has changed in ice‐free areas in the past (Tables 1 and 2). Since these plants are found in many ice‐free areas, they can be used to reconstruct past climates going back from the present (mosses) to hundreds and even thousands of years (peat records) around the continent and on sub‐Antarctic islands. Records in Antarctica started accumulating approximately 2.8 ka (Charman et al., 2018), while in the sub‐Antarctic most span from the early or mid‐Holocene to present (e.g., Bergstrom et al., 2002). However, some begin at the end of, or during, the last glacial (McGlone et al., 1997; Selkirk et al., 1988) (Figures 2a,b, and 3a).

**TABLE 1:** Examples of recent studies of biological archives present in the Southern Ocean and Antarctica and corresponding analytical techniques that are being applied to them to investigate past climatic and ecological change

	Modern DNA	Ancient DNA	Isotopic dating	Stable isotopes	Organic geochemical analysis	Elemental analysis	Macrofossils/pollen	Diatoms, testate amoebae, radiolarian species	Growth rings
Moss beds and peat	(Biersma et al., 2020; Biersma, Jackson, Bracegirdle, et al., 2018; Biersma, Jackson, Stech, et al., 2018; Pisa et al., 2014)		(Amesbury et al., 2017; Clarke et al., 2012; Robinson et al., 2018)	(Royles & Griffiths, 2015; Yu et al., 2016; Amesbury et al., 2017; Robinson et al., 2018; Stelling & Yu, 2019)	(Loisel et al., 2017)	(Van der Putten et al., 2015)	(McGlone et al., 2000; Royles et al., 2012; Stelling et al., 2018)	Testate amoebae (Amesbury et al., 2017; Charman et al., 2018) Diatoms (Bishop et al., 2021)	
Terrestrial invertebrates	*Belgica antarctica* (Antarctic midge) (Kelley et al., 2014); *Cryptopygus sverdrupi* (Collembola) (Stevens & D'Haese, 2014); Collembola species (Collins et al., 2020); Freshwater crustacea (Maturana et al., 2020); Weevils (Baird et al., 2021)						Antarctic mites (Hodgson & Convey, 2005); *Pachnobium dreuxi* (Ectemnorhinini weevil) (Chapelin‐Viscardi et al., 2010)		
Lake sediments		(Bissett et al., 2005; Coolen et al., 2004; Ficetola et al., 2018)	(Berg et al., 2020; Hodgson, Whitehouse, et al., 2016; Saunders et al., 2018)	(Browne et al., 2017; Noon et al., 2003; Royles et al., 2012)	(Berg, White, Jivcov, et al., 2019; Hodgson et al., 2006; Loisel et al., 2017)	(Govil et al., 2016; Píšková et al., 2019; Roberts et al., 2017)	(Gibson et al., 2007; Strother et al., 2015; Zwier et al., 2022)	Diatoms (Perren et al., 2020; Roberts & McMinn, 1999; Watcham et al., 2011)	
Animal colonies	*Arctocephalus gazella (*Antarctic fur seals) (Cleary et al., 2021), *Eudyptes* sp. (Macaroni, Rock hopper and Royal penguins) (Frugone et al., 2018), Weddell seal, (*Leptonychotes weddellii*) (Younger et al., 2016)	*Aptenodytes forsteri* (Emperor penguin) (Li et al., 2014), *Mirounga leonina* (southern elephant seal) (de Bruyn et al., 2014)	*Pagodroma nivea* (snow petrel) (Berg, Melles, et al., 2019), *Aptenodytes forsteri* (Emperor penguin) (Li et al., 2014), *Aptenodytes forsteri* (Emperor penguin) (Huang et al., 2016)	*Aptenodytes forsteri* (Emperor penguin) (Huang et al., 2016) *Pagodroma nivea* (snow petrel) (McClymont et al., 2022)	*Pagodroma nivea* (snow petrel) (Berg, Melles, et al., 2019), seals (Huang et al., 2011)	*Pagodroma nivea* (snow petrel) (Berg, Melles, et al., 2019), *Pygoscelis adeliae* (Adelie penguin) (Xu et al., 2020), *Aptenodytes forsteri* (Emperor penguin) ( Huang et al., 2016)	*Pygoscelis adeliae* (Adelie penguin) (Xu et al., 2020), *Aptenodytes forsteri* (Emperor penguin) (Huang et al., 2016), seals (Huang et al., 2011)	*Pagodroma nivea* (snow petrel) (Berg, Melles, et al., 2019)	
Benthic marine invertebrates	*Ophionotus victoriae* (brittle star) (Lau et al., 2021; Strugnell et al., 2012) *Nacella concinna* (limpet) (González‐Wevar et al., 2016) *Paralomis birsteini* (king crab) (Hellberg et al., 2019)		*Errina* sp. (stylasterid coral) (King et al., 2018), *Desmophyllum dianthus*, *Caryophyllia* spp., *Paraconotrochus antarcticus* (scleractinian, coral) (Wilson et al., 2020)	*Laternula elliptica* (bivalve) (Tada et al., 2006), *Cellaria incula, C. nutti, C. nodulata* (bryozoan) (Smith, 2007)		*Trematomus bernacchii*, *T. pennellii*, *Pagothenia borchgrevinki* (ice fish) (McMullin et al., 2017) *Laternula elliptica* (bivalve) (Wing et al., 2020)	*Desmophyllum dianthus*, *Caryophyllia* spp., *Paraconotrochus antarcticus* (scleractinian, coral) (Wilson et al., 2020)		*Aequiyoldia eightsii* (bivalve) (Román‐González, Scourse, Butler, et al., 2017), *Adamussium eightsii* (bivalve) (Cronin et al., 2020)
Marine shelf sediments			(Kingslake et al., 2018; Smith et al., 2010)	(Lund et al., 2021; Swann et al., 2013)	(Lamping et al., 2021; Shevenell et al., 2011; Vorrath et al., 2019)	(Ashley et al., 2021; Hillenbrand et al., 2017)		Diatoms (Kingslake et al., 2018; Scherer et al., 2007) Radiolarians (Lawler et al., 2021)	

*Notes*: Isotopic dating methods include radiocarbon dating (^14^C), lead‐210 (^210^Pb), caesium‐137 (^137^Cs), stable isotopes includes: δ^13^C, δ^15^N, δ^18^O; organic geochemical analysis include biomarkers and organic matter characterization techniques such as cholesterol, cholestanol, fluorescence and UV absorbing pigments, liquid chromatography organic carbon detection (LC‐OCD), ultrahigh‐resolution Fourier‐transform ion cyclotron resonance mass spectrometry (FT‐ICR‐MS), organic matter concentration, elemental analysis includes ion chromatography (IC) and inductively coupled plasma‐atomic emission spectroscopy (ICP‐AES), Itrax X‐ray fluorescence scanning, ion beam analysis (IBA), laser ablation, secondary ion mass spectrometry (SIMS), Macrofossils include hairs, bones, egg shells etc, Diatom species recovered from seal, snow petrel, and penguin diets are being used to indicate freshwater, brackish, and marine environments, Growth rings refer to the periodic accretional patterns in hard body structures such as shells and corals. See Supplementary information 1 for additional examples.

**TABLE 2:** Past climatic, environmental and ecological variables investigated using Southern Ocean and Antarctic biological archives, and their strengths and limitations. Limitations that apply to all proxies and archives are discussed in the main text

Archive	Application	Strengths	Limitations
Moss beds and peat	Microclimatic environmental variables including: Air temperature (Amesbury et al., 2017; Foster et al., 2016) Nutrient sources (Bergstrom et al., 2002; Wasley et al., 2012) Seasonality (Charman et al., 2018) Water availability (Amesbury et al., 2017; Bramley‐Alves et al., 2015; Robinson et al., 2018; Royles & Griffiths, 2015)	‐High‐resolution (e.g., seasonal‐annual) ‐Micro‐climate information ‐Usually continuous record preserved for long periods of time (up to thousands of years) ‐Biological proxies (e.g. testate amoebae) respond quickly to environmental changes	‐Slow growth rates reduce precision of dating and increase uncertainty ‐Susceptible to disturbance, weathering, erosion ‐Can have marked changes in growth rates ‐Do not appear in areas outside of their physiological limits (i.e. lack of spatial distribution as proxy data) ‐Mixing of timing horizons can occur through root growth ‐Coalescent‐based analyses to infer demographic are influenced by numbers of individuals and populations, and the sampling of polymorphisms ‐Small sample sizes tend to underestimate, or fail to detect, population expansion for coalescent‐based analyses
Terrestrial invertebrates	Deglaciation/inundation (Baird et al., 2021) Ice free areas/refugia (Collins et al., 2020; Kelley et al., 2014; Stevens & D'Haese, 2014) Impact of human activities (Anthropocene change) (Baird et al., 2020)	‐Distributed throughout ice‐free Antarctica and sub‐Antarctic islands ‐Can be easily collected in the field ‐Taxonomy of major taxa (e.g. mites, weevil, springtail) is relatively well‐resolved for genetic inferences	‐Only offer proxy to past and current environmental changes in locations where samples are collected ‐Lack of baseline information (e.g. growth, reproduction, mutation rate) to calibrate event dating ‐Signals of past events can be masked or eroded by noise (e.g. changes in allele frequency due to severe bottlenecks) ‐Quality of biological samples degrade in long term storage which may limit genetic inferences ‐Coalescent‐based analyses to infer demographic change are influenced by numbers of individuals and populations, and the sampling of polymorphisms ‐Small sample sizes tend to underestimate, or fail to detect, population expansion for coalescent‐based analyses
Lake sediments	Air temperature (Foster et al., 2016) Deglaciation/inundation (Berg, White, Jivcov, et al., 2019; Verleyen et al., 2011) Ice free areas/refugia (Cromer et al., 2006; Gibson & Bayly, 2007) Impact of human activities (Anthropocene change) (Ficetola et al., 2018; Saunders et al., 2013) Nutrient sources (Choudhary et al., 2022) Primary productivity (Chen et al., 2013) UV environment (Hodgson et al., 2005; Verleyen et al., 2005) Salinity (Roberts & McMinn, 1999; Roberts et al., 2001) Southern Hemisphere westerly winds (Perren et al., 2020; Saunders et al., 2018) Vegetation change (Zwier et al., 2022)	‐Abundant throughout ice‐free Antarctica and sub‐Antarctic islands ‐Multi‐proxy analyses possible ‐Multi‐decadal to multi‐millennial resolution ‐Span at least to Holocene to Last Interglacial ‐Link ice sheets and glaciers to the sea ‐Usually continuous ‐Biological proxies (e.g. diatoms) respond quickly to environmental changes	‐Can be difficult separating causes of change (e.g., climate vs. environment vs. human impacts) ‐Chronologies rely on absence of sediment mixing ‐Potential radiocarbon reservoir effects ‐Preferential preservation of biological proxies ‐Limited potential for calibration to observational data ‐Challenging to obtain *seda*DNA due to tiny amounts, thus contamination with modern DNA is an issue ‐Strict contamination control required ‐Reference genetic databases required to make full use of metagenomic approaches
Animal colonies	Deglaciation/inundation (Berg, White, Hermichen, et al., 2019; Cleary et al., 2021; de Bruyn et al., 2009) Ice free areas/refugia (Berg, Melles, et al., 2019; de Bruyn et al., 2009) Impact of human activities (Anthropocene change) Nutrient sources (Huang et al., 2011) Ocean circulation (Trucchi et al., 2014) Primary productivity/surface productivity (Berg, Melles, et al., 2019; Emslie, 2021; Xu et al., 2020) Sea ice (Berg, Melles, et al., 2019; Cleary et al., 2021; de Bruyn et al., 2014; McClymont et al., 2022)	‐Multi‐proxy analyses possible ‐Data types include archival materials from ancient colonies, and genetic data from extant individuals ‐Proxies to paleo‐ and modern environments ‐Provide direct evidence of potential impacts from direct and indirect human activities ‐Well‐preserved ancient DNA in ancient colonies provides ability to calibrate molecular clocks ‐Generation times well understood	‐Terrestrial animal colony information can rarely be extracted from other types of archives ‐Sampled materials can be patchy in distribution due to fieldwork constraints ‐Signals of past events can be masked or eroded by noise (e.g. changes in allele frequency due to severe bottlenecks) ‐Quality of biological samples degrade in long term storage which may limit genetic inferences ‐Coalescent‐based analyses to infer demographic change are influenced by numbers of individuals and populations, and the sampling of polymorphisms ‐Small sample sizes tend to underestimate, or fail to detect, population expansion for coalescent‐based analyses
Benthic marine invertebrates	Air temperature (Brey et al., 2011) Fast ice duration (Román‐González, Scourse, Richardson, et al., 2017) Ice free areas/refugia (González‐Wevar et al., 2016; Lau et al., 2021; Strugnell et al., 2012) Ocean circulation (including Circumpolar deep water) (King et al., 2018; Wilson et al., 2020) Ocean temperature (Román‐González, Scourse, Richardson, et al., 2017) Primary productivity/surface productivity (Brey et al., 2011; Cronin et al., 2020; Román‐González, Scourse, Butler, et al., 2017; Smith, 2007) Salinity (Tada et al., 2006) Sea ice (Cronin et al., 2020; McMullin et al., 2017; Wing et al., 2020)	‐Data resolution range from local to circum‐Antarctic scale (including sub‐Antarctic islands and the deep sea) ‐Multiple types of analyses from one specimen (e.g., sclerochronology, DNA) ‐ Information can span years (sclerochronology; within a specimen's life time) or since Miocene (DNA; time since the emergence of Antarctic benthic fauna) ‐Sclerochronology can be annually resolved and absolutely dated	‐Taxonomic uncertainties in many species, which hinder the interpretation of species‐level and population‐level data ‐Lack of baseline information (e.g. growth, reproduction, mutation rate) to calibrate event dating ‐Signals of past events can be masked or eroded by noise (e.g. changes in allele frequency due to severe bottlenecks) ‐Quality of biological samples degrade in long term storage which may limit genetic inferences ‐Coalescent based analyses to infer demographic change are influenced by numbers of individuals and populations, and the sampling of polymorphisms ‐Small sample sizes tend to underestimate, or fail to detect, population expansion for coalescent‐based analyses
Advances in investigating marine shelf sediments	Ice sheet advance/retreat (Kingslake et al., 2018) Ocean circulation (Crosta et al., 2022) Ocean temperature (Gersonde et al., 2005; Shevenell et al., 2011) Primary productivity/surface productivity (Ashley et al., 2021; Leventer et al., 2006) Sea ice (Gersonde et al., 2005; Smith et al., 2010; Swann et al., 2013; Vorrath et al., 2020)	‐Multiple types of analyses on one record (e.g., biological, (bio)geochemical, physical) providing multiple lines of evidence of biodiversity, climate, ecosystem and environmental changes ‐Most span the Holocene or longer ‐Multi‐decadal to multi‐millennial resolution ‐Provide a link between the ice‐covered and ice‐free land, sea ice zone and the ocean ‐Respond to multiple climate and environmental parameters, in most cases providing a general overview of changes ‐Species assemblages and relative abundances of planktonic organisms change quickly in response to environmental change.	‐Reference databases required to make full use of genetic and microfossil approaches ‐Often lacking biogenic material (foraminifera, diatoms) ‐Chronologies rely on absence of sediment mixing ‐Challenging to obtain *seda*DNA due to tiny amounts ‐Strict protocols required to prevent contamination of sediments with modern DNA ‐Protocols for sedaDNA not yet well‐established ‐Erosion during ice advance limits utility in obtaining continuous records back past the last glaciation

*Note*: See Supplementary information 1 for additional examples.

Mosses quickly colonize newly exposed ice‐free areas as glaciers retreat, where summer sunlight melts nearby snow banks, providing vital water for organisms to emerge from the long winter and grow throughout the short summer season. Undisturbed, these mosses can then grow for hundreds or thousands of years (Amesbury et al., 2017; Clarke et al., 2012; Robinson et al., 2018), provided they receive sufficient summer melt and nutrients, and sunlight is available (Wasley et al., 2012) (Figure 2a,b). If conditions are too extreme to support growth, the mosses can persist in the location and growth will recommence if conditions improve (Cannone et al., 2017; Roads et al., 2014), or, alternatively, the moss beds may become the substrate on which lichen communities establish and thrive (Bishop et al., 2021; Wasley et al., 2012). Changes from mosses to lichens induced by environmental change are accompanied by changes in the communities that live within these vegetation types (e.g., diatom composition, see Bishop et al., 2021). On the Antarctic Peninsula, mosses often provide a nursery for establishment of vascular plants, which may then outcompete them (Bokhorst et al., 2022; Casanova‐Katny & Cavieres, 2012).

Growth rates are extremely slow (0.2 to 5.6 mm year^−1^, Convey et al., 2014) and mosses represent the Antarctic version of dendrochronology, but on a miniature scale. Mosses lack vascular tissue, which means carbon is sequestered into the section where it was originally fixed in photosynthesis, similar to tree rings. Radiocarbon methods (and especially ‘bomb pulse’ radiocarbon, which measures additional ^14^C introduced into the atmosphere via nuclear weapons testing in the 20th century) can be used to date mosses, which is often complemented by ^210^Pb dating for the last ca. 100–150 years (Amesbury et al., 2017; Clarke et al., 2012; Robinson et al., 2018). Depending on growth rates the resolution in dated moss sections range from sub‐annual to decadal. Resolution is mainly limited by the slow growth rates, which necessitate measurements of longer shoot samples that encompass multiple years of growth. The growth rates of plants like mosses are intimately connected to the microclimate they are found in (Lembrechts & Lenoir, 2019).

Pollen, spores, and plant macrofossils are commonly used to reconstruct vegetation change in peat records in the Antarctic and sub‐Antarctic (e.g., Stelling et al., 2018; Van der Putten et al., 2012, 2015). Another biological proxy found in peat records is testate amoebae, which have been used as indicators of microbial productivity and mass accumulation rates. Most work has been conducted in the Antarctic Peninsula region, where changes have been interpreted as a response to temperature and/or precipitation variability (Amesbury et al., 2017; Royles et al., 2013). Limited work has been undertaken in the sub‐Antarctic, where the focus has been on the link between changes in the taxonomic composition of testate amoebae communities and their biomass, to the input of wind‐blown oceanic sea salt aerosols onto peatlands, highlighting the potential for testate amoebae to be used as a proxy for past wind conditions (Whittle et al., 2019). Testate amoebae have also been used to develop quantitative temperature reconstructions (Charman et al., 2018). These are based on correlating testate amoebae productivity and mass accumulation rates with meteorological temperature data from nearby stations (e.g., Charman et al., 2018). Direct interpretations of proxy records in terms of climate data recorded at meteorological stations are complicated, as biological indicators are generally influenced by, and in fact integrate, more than one climate factor (Royles et al., 2016). Microclimatic variation also needs to be considered (Lembrechts and Lenoir 2019). The development of more comprehensive, and more appropriately scaled, models for climate across the region would represent a major advance (Maclean, 2020) and multiple proxies offer a better chance of developing these.

Additionally, Antarctic plants record signatures of the environment as they grow, in particular in the carbon (δ^13^C), nitrogen (δ^15^N), and oxygen (δ^18^O) stable isotope ratios contained in their tissues (Amesbury et al., 2017; Bramley‐Alves et al., 2015; Robinson et al., 2018; Royles & Griffiths, 2015). δ^13^C in moss cellulose correlate with the degree of saturation of moss turfs by water, with external water films slowing diffusion of gases into the moss cells, and subsequently reducing the ability of Rubisco (enzyme) to discriminate against ^13^C (Bramley‐Alves et al., 2015; Royles & Griffiths, 2015). This has allowed reconstructions of microclimate water availability in coastal Antarctica. Records of δ^13^C in moss cellulose confirm that the Antarctic Peninsula has become warmer and wetter (Amesbury et al., 2017) and have revealed a drying trend in the Windmill islands, East Antarctica over the past half‐century (Clarke et al., 2012; Robinson et al., 2018). These data are supported by other biological proxies such as diatoms in lake sediments, which corroborate changes in water availability (Roberts et al., 2006). δ^13^C in moss cellulose has also been used to estimate photosynthetic limitation by CO_2_ supply and model CO_2_ assimilation rate, which has implications for understanding carbon cycling (Royles et al., 2012).

There is also potential for δ^18^O in moss cellulose to provide information on water sources and moisture conditions, although these techniques are less well developed (Royles et al., 2016; Stelling & Yu, 2019). Nevertheless, δ^18^O in moss cellulose has been used in combination with δ^13^C to develop paleoclimate records for the Antarctic Peninsula, where over the last ca. 1700 years, two distinct intervals of dry conditions were identified between 600–950 CE and 1450–1950 CE (Stelling & Yu, 2019). Further development of stable isotope techniques to more widely incorporate δ^18^O should enhance the value of the records available from moss cellulose. Essential to this would be to confirm how moss δ^18^O responds to environmental change, which requires controlled growth experiments, such as those performed by Bramley‐Alves et al. (2015).

δ^15^N found in chlorophyll and plant proteins record sources of nutrients for terrestrial vegetation and their invertebrate communities in Antarctica (Bokhorst et al., 2019; Lee et al., 2009; Wasley et al., 2012) and the sub‐Antarctic (Erskine et al., 1998). However, despite its potential as an indicator of past nutrient sources, δ^15^N has rarely been used as a paleo‐proxy in the region, other than in the sub‐Antarctic, where δ^15^N signatures in fossil peat at Macquarie Island mainly reflect past changes in the proportion of plant nitrogen derived from animal sources (Bergstrom et al., 2002).

Pigments (e.g., flavonoids) preserved in plant cells can be extracted and identified by high‐pressure liquid chromatography (HPLC, Waterman et al., 2017, 2018). Tracing changes in such pigments down moss plants could identify if past climates were more or less stressful for growth. Meanwhile, signatures of vascular plants based on identification of lignin phenols and neutral sugars can be used to determine periods of longer and warmer growing seasons (Loisel et al., 2017). Combined with genomic techniques, such methods could investigate if increased ultraviolet radiation, as a result of ozone depletion, has accelerated mutation in these haploid organisms or if they have responded by increasing production of sunscreen pigments. These tiny plants can provide a climate history not just for themselves but also the microbes, fungi, and invertebrates that live among them.

## 4 TERRESTRIAL INVERTEBRATES

Groups such as nematodes, tardigrades, rotifers, and mites are found on many of the ice‐free areas of the continent and in the sub‐Antarctic (Phillips et al., 2022) (Figures 1, 2, and 3b). Springtails are relatively common too, but absent from some parts of East Antarctica (Baird et al., 2019). The broad distributions, reasonable abundance and species richness of the groups provide a resource for understanding the evolution of the Antarctic terrestrial landscape.

For understanding Earth history and its influence on biodiversity, traditional DNA‐ and RNA‐based methods, such as those using limited mitochondrial and nuclear DNA and molecular clock estimates, have for some time been applied to Antarctic terrestrial groups, including invertebrates (Moon et al., 2017) (Figure 2b; Tables 1 and 2). These studies have generally confirmed a residence time in refugia for populations of various species dating further back than the LGM, resulting in calls to revise the paradigm of general extinction of the continental biota during the LGM (Collins et al., 2020; Convey et al., 2008; Kelley et al., 2014; Short et al., 2022; Stevens & D'Haese, 2014). For the sub‐Antarctic, many results published in earlier studies confirmed substantial dispersal across the region, including to the continent, among islands, and to other continental areas (e.g., Mortimer et al., 2011; Stevens et al., 2006), while also confirming the significance of local vicariance due to glaciation or volcanic activity, including through refugia in the LGM, on population processes (Mortimer & Jansen van Vuuren, 2006).

Subsequent work, based on more recent techniques, such as genome‐wide single nucleotide polymorphisms or phylogenomics, have largely confirmed the early studies' focus on ice‐free refugia pre‐dating the LGM, through refining estimates of colonization patterns and dispersal frequency (Baird et al., 2020). One of the few studies using fossil data to calibrate phylogenomic‐based trees shows close relationships exist between Earth history and the diversification of both marine and terrestrial Antarctic taxa (Baird et al., 2021). All of these DNA‐based studies, along with others based on the sedimentary record of terrestrial or aquatic species (e.g., Pinseel et al., 2021), demonstrate their value not only for constraining the timing of major environmental changes but also for understanding the reciprocal interactions between Earth and life through time. One of the major opportunities that animal genomic approaches afford is better understanding connectivity between ice‐free areas and the timing of exposure of these areas. In conjunction with cosmogenic nuclide approaches and other proxies, genomics may offer considerable power to help constrain models of ice sheet dynamics throughout the Quaternary and deeper time (Convey et al., 2020).

## 5 LAKE SEDIMENTS

Lakes occur throughout ice‐free areas of Antarctica and on many sub‐Antarctic islands (Figures 1, 2, and 3c). They range from small ponds to lakes greater than 100,000 km^2^ (Gibson et al., 2006). Benthic cyanobacteria and diatoms dominate their biomass, and aquatic mosses are among the highest forms of plant life (Hodgson et al., 2004). Most records span part or all of the Holocene (last 12 ka; e.g., Saunders et al., 2018; Verleyen et al., 2011), although exceptions exist in Antarctica, with records extending prior to the LGM (e.g., Hendy, 2000; Hodgson et al., 2006) (Figure 2b). The use of lake sediments for paleoclimate, paleoecological and/or paleoenvironmental studies have a long history in the region due to their spatial extent, relative abundance compared to other archives, potential for different types of analyses, and broad range of questions that can be addressed (Tables 1 and 2).

In recent years, the development and application of high‐resolution scanning techniques has significantly expanded the potential for detailed sediment core images and the amount and types of biogeochemical and geochemical data obtainable from a lake sediment core (Table 1). For example, micro x‐ray fluorescence (Berg, White, Jivcov, et al., 2019; Perren et al., 2020; Roberts et al., 2017) and hyperspectral imaging in the visible and near‐infrared range (Aymerich et al., 2016; Saunders et al., 2018) have been used to determine inputs of minerals related to catchment dynamics, such as evidence of glacier fluctuations (Berg, White, Jivcov, et al., 2019), impact of volcanism on past penguin populations via signatures of guano and guano‐related elements (Roberts et al., 2017), and changes in Southern Hemisphere westerly wind strength (Perren et al., 2020; Saunders et al., 2018). Elsewhere, both methods have been used for understanding within‐lake processes, such as past aquatic productivity (Davies et al., 2015; Zander et al., 2022), demonstrating broader potential application in the region. It is important to note, however, that data need to be verified and calibrated using more conventional methods such as x‐ray diffraction and HPLC (Davies et al., 2015; Píšková et al., 2019; Zander et al., 2022).

Organic matter characterization techniques such as fluorescence analysis and liquid chromatography organic carbon detection have been applied to Antarctic lake waters to understand biological production and biogeochemical cycling (Kida et al., 2019). These techniques, if applied to soil‐water extracts obtained from lake sediment cores, have the potential to provide information on past biological activity for the region. For example, marine biomarkers and methods for characterizing dissolved organic matter have been used in ice cores to reconstruct regional environmental conditions during the Antarctic Cold Reversal (14.6–12.7 ka, (Fogwill et al., 2020)), and a similar approach could be applied to lake sediments.

Application of a broader range of isotopic techniques (e.g., stable carbon including both dissolved organic carbon (δ^13^C_DOC_) and dissolved inorganic carbon (δ^13^C_DIC_), oxygen (δ^18^O), hydrogen (δ^2^H), and nitrogen (δ^15^N) isotopic ratios) would help understand how nutrients cycle in terrestrial lake environments with changing temperatures. As glaciers and ice sheets melt, and subglacial groundwater discharges into marine environments, Antarctica is likely to be an important source of atmospheric carbon dioxide not yet included in the global carbon budget (Connolly et al., 2020; McDonough et al., 2022). Determining the bioavailability of ancient organic matter and whether it influences downstream marine ecosystems in the Southern Ocean is yet to be understood. Of particular relevance to quantitative paleoclimate reconstructions, extraction of sedimentary glycerol dialkyl glycerol tetraethers (GDGTs) from lake sediments provides an opportunity for quantitative temperature reconstructions, which has so far only been applied once in the region (Foster et al., 2016).

Genetic techniques have infrequently been applied to lake sediments in the Antarctic region (i.e., *seda*DNA) (Bissett et al., 2005; Coolen et al., 2004; Ellis‐Evans, 1996; Ficetola et al., 2018), (Table 1), despite their potential being recognized since at least the mid‐1990s (Ellis‐Evans, 1996). As demonstrated elsewhere, such as in the Arctic, use of genomic techniques combined with conventional analyses have the potential to reconstruct currently unknown trophic interactions and evolutionary adaptation to changing environments and climates in the Antarctic region (Cuenca‐Cambronero et al., 2022; Ellegaard et al., 2020).

These studies demonstrate the potential for combined use of established methods with high‐resolution scanning techniques on intact cores providing detailed core images and extensive biogeochemical and geochemical data, together with recent advances in organic matter characterization, biomarkers (such as GDGTs), and genomic methods. This approach could provide a more holistic view of past ecosystems and their interactions with climate and environmental changes than previously possible.

## 6 ANIMAL COLONIES

Marine predators, such as penguins, seals and snow petrels, provide a wealth of high‐resolution information regarding paleoenvironments and climates through investigation of their preserved remains in ancient colonies (e.g., Berg, White, Hermichen, et al., 2019; Emslie, 2021), genetic data sequenced from extant individuals (e.g., Cleary et al., 2021), or a combination of these methods (de Bruyn et al., 2009) (Figures 2 and 3d, Tables 1 and 2). Depending on the species, ancient colonies can contain layers of materials, such as guano, bones, egg shells, feathers, hairs, skin, stomach oil (known as mumiyo), preserved tissues, and prey or dietary remains (Figure 2a; Table 1). These archives are studied using a range of complementary analytical techniques including radiocarbon dating, stable isotopes, high‐resolution organic matter fluorescence, lipid biomarkers, gross morphological approaches, and ancient DNA (aDNA) (Table 1). Together these have been used across several species to infer age constraints on the availability of ice‐free habitat available for breeding, which directly reflects the timing of deglaciation or inundation at terrestrial sites (Younger et al., 2016).

Analyses of dietary and/or stable isotope composition of animal remains reflects the environment in which the animals were feeding (e.g., in polynyas versus loose pack ice), which sheds light on sea ice conditions, and more broadly, can inform us of species responses to environmental change (Berg, White, Hermichen, et al., 2019; McClymont et al., 2022) (Tables 1 and 2). Several studies indicate that episodic occupation of sites by Adélie penguins on the Antarctic continent (e.g., Xu et al., 2020) reflects periods of enhanced marine productivity and greater nesting site availability during past warmer climates, in particular the penguin ‘optimum’ (~2–5 million years ago, Ma) (see Younger et al. (2016) and references within). Seal hairs (Hodgson & Johnston, 1997) and changes in the deposition of bio‐elements from penguin guano (Sun et al., 2000) can be used as proxies for population size; the latter study finding a peak in population size corresponding to a time of high precipitation (~1.4–1.8 ka), suggesting a climatic influence. Elemental concentrations for seal populations have so far focused on changes during the 20th century in response to human activities (e.g., Huang et al., 2011; Yang et al., 2010). However, there is scope to extend these records back further to look at natural variability in animal populations.

Coalescent‐based methods (primarily Bayesian skyline plots [BSPs] (Drummond et al., 2005) to date) have also been applied to genetic data sequenced from modern individuals to estimate past changes in effective population size (Ne) over time (Figure 2b; Tables 1 and 2). These support findings from previous studies of ancient colonies (e.g., Cleary et al. (2021)) and are mostly used to provide additional information over older time scales (Figure 2b). Large historical increases in population size have been reported in Adélie, Chinstrap, Emperor, Gentoo, King and Macaroni penguins, and some but not all species of Rockhopper penguins, in response to increased availability of ice‐free breeding habitat and/or more productive foraging habitats resulting from warmer climates (Frugone et al., 2018; Younger et al., 2016). In general, the accuracy of BSPs is influenced by numbers of individuals and populations, and the sampling of polymorphisms (see Grant, (2015) for a review). Small sample sizes tend to underestimate, or fail to detect, population expansions. Furthermore, markers with low levels of polymorphism are unable to resolve recent events, and pooling across populations can mask signals of population change when populations are genetically heterogenous.

Importantly, several studies of penguins and seals have demonstrated the power of integrating aDNA sequenced from historic colonies with genetic data from modern animals (e.g., de Bruyn et al., 2009; Millar et al., 2008) (Table 1). aDNA appears to be well preserved in the cold Antarctic environment and samples from historic colonies have been sequenced from as old as ~44 ka (Subramanian et al., 2009). aDNA has allowed investigation into the response of southern elephant seals to changing habitat availability resulting from the retreat of the grounded ice sheet 7.5–8 ka and has been used to locate the source population (Macquarie Island) for a now extinct breeding site situated on the Victoria Land coast (Ross Sea) (de Bruyn et al., 2009). Such studies give insight into the rate and demographic mechanisms of colonization and decolonization resulting from changing environments. Furthermore, sequencing of aDNA allows direct calculation of a molecular evolutionary rate, which is preferable to obtaining rates from species level phylogenetic trees (calibrated with fossils or vicariance events), as these can massively overestimate timings when applied to populations (Grant, 2015). aDNA can, therefore, provide greater confidence in the use of modern DNA to date demographic change over extended time periods (Figure 2b).

Changes in sea ice cover driven by climate change are predicted to impact access to productive foraging grounds and breeding habitats for many marine predators, and these will drive population changes, with increases likely for some species (e.g., Gentoo penguins) and decreases for others (e.g., Adélie, Chinstrap, Emperor and King penguins) (Constable et al., 2022). Further interrogation of biological archives to determine differential species responses to the same environmental drivers will inform future species‐specific conservation strategies. As more ancient animal colonies are exposed due to climatic change, with recent snow melt over the past ~50 years (Emslie, 2021), it is a sad irony that these events will likely provide further opportunities to understand how past environments and climates impacted colonial animals.

## 7 BENTHIC MARINE SPECIES

The Southern Ocean contains a taxonomically diverse fauna of ~9,000 species, the vast majority (88%) of which are benthic (De Broyer & Danis, 2011) (Figure 2a and 3e). The evolution of this unique fauna has been shaped by the oceanographic isolation of the Southern Ocean (caused by the initiation of the ACC, ~34 Ma) and Quaternary glacial cycles, which have driven grounded ice sheets out over the shelf during glacial maxima. Scouring of the continental shelf by ice sheets would have destroyed much of the fossil evidence of recent benthic assemblages (Barnes & Clarke, 2011). However, the modern benthos, alive in Antarctica today, are demonstrating their utility as biological archives of environmental change (Lau & Strugnell, 2022).

Many Southern Ocean benthic taxa lack pelagic larval stages within their development, and several are slow growing (e.g., Barnes et al., 2006; Dahm & Brey, 1998) and long‐lived, (e.g., Burgess et al., 2010; Henry & Torres, 2013), features that contribute to their utility as archives (Figure 2b; Table 2). The hard body structures (e.g., shell, corals) of Antarctic marine species that are laid down in periodic accretional patterns (growth rings) can reflect environmental drivers at the time they were deposited (see Roman Gonzalez (2021) and within). This technique relies on growth rings in individuals being deposited similarly across a population in response to common environmental drivers, and in some cases, growth rings can be annually or seasonally resolved (as is the case for ice cores) and absolutely dated (Peck & Brey, 1996). The physical counting of growth increments (Table 1) to estimate maximum life span has utility in informing generation time, a necessary variable for some demographic analyses using genetic data (see below). In addition, these hard structures can be interrogated using a range of analytical techniques including isotopic ratios, radiocarbon dating, and X‐ray photography (Table 1) and are providing insights into a range of paleoenvironmental and climate conditions (Table 2).

Investigations across a range of Southern Ocean invertebrates have reported signatures of paleoenvironmental conditions contained within growth rings. For example, growth rates have been linked to the duration of past primary productivity (reflecting sea ice extent) in a range of bryozoans (Barnes, 1995; Barnes, 2017; Clark & Peat, 2022; Smith, 2007) and the bivalve, *Aequiyoldia eightsii* (Román‐González, Scourse, Richardson, et al., 2017) (Tables 1 and 2). Important discoveries have been made through isotopic dating of corals whereby signatures of circumpolar deep water (CDW) were recorded, demonstrating their ability to provide insight into the past location and movement of water masses (Table 2). CDW is warmer, and generally deeper, than other water bodies and its intrusion onto the shelf can lead to melting of ice shelves and subsequent loss of grounded ice, which is an important driver of sea level rise. Using radiocarbon to investigate extant deep‐sea stylasterid corals, concurrent signatures of CDW upwelling were detected in distant locations (Ross Sea and Wilkes Land margins) aligned with the end of the Little Ice Age (~1830 CE) (King et al., 2018). Through the application of neodymium isotopes to fossil deep‐sea scleractinian corals, Wilson et al. (2020) reported signatures of Lower CDW throughout the LGM and subsequent deglaciation, suggesting sea ice control on deep ocean structure (Tables 1 and 2). Such studies demonstrate the power of these coral archives in understanding past changes in oceanic water bodies—important drivers of local and global climate change, and their potential to provide key information for constraining climate models.

Additional circum‐Antarctic collection and analysis of extant and sub‐fossil samples from overlapping time periods, including museum samples, should enable signatures to be matched across samples, regions, and even longer time series to be established. Given the fragile nature of these corals, collection via remotely operated vehicles will help maintain sample integrity. Importantly, climate change is threatening calcifying organisms in the Southern Ocean through increased ocean acidification, particularly those with high magnesium calcite or aragonitic skeletons (Figuerola et al., 2021), which means existing archives may be erased in the future.

As is the case for penguins and seals, genetic data sequenced from modern benthic marine species are being analysed using increasingly sophisticated methods to investigate patterns of past demographic change. Determining where species persisted during glacial maxima throughout the Quaternary remains an active research area with implications for understanding biology and constraining climate modelling. Simple signatures visualized from mitochondrial data (Allcock & Strugnell, 2012) sequenced from benthic species, have been complemented with coalescent‐based analyses that indicate potential refugial (ice‐free) locations on the continental shelf. Using this method, suggestions have been made for locations in the Weddell Sea (pycnogonid *Nymphon australe*, octopus *Pareledone turqueti*), Ross Sea (*P. turqueti*), East Antarctica (echinoderm *Sterechinus neumayeri*), and Adélie Land (*P. turqueti*) (Díaz et al., 2018; Lau et al., 2020; Soler‐Membrives et al., 2017; Strugnell et al., 2012) (Tables 1 and 2). There are several challenges in using these approaches, however. In addition to the limitations of BSPs (see Animal colonies above), genetic signatures from the LIG can be eroded from the marked environmental change (i.e., habitat loss) that occurred during the LGM. Furthermore, obtaining well‐preserved samples is logistically challenging and expensive, and those existing in museum collections, while valuable, can be degraded.

Increasingly sophisticated methods using whole genomic data promise greater power in pinpointing the localities that remained ice free during previous glacial maxima, as well as understanding the extent of ice‐free areas during glacial minima—an important reference for current climate change. In particular, the DNA contained within extant organisms can be used to investigate the potential existence of historic marine seaways that may have been present across Antarctica during the LIG period (i.e., West Antarctic ice sheet collapse), and thus can provide a proxy for ice mass loss (Strugnell et al., 2018). If historic marine seaways existed, then marine organisms would have moved across these seaways and signatures of historic gene flow will be contained within their genomes. This information could assist in refining model sensitivity for projecting future sea level rise.

## 8 ADVANCES IN INVESTIGATING ANTARCTIC MARINE SHELF SEDIMENTS

Marine sediment archives consist of organic, biogenic and inorganic particles and sediment that accumulate on the seafloor over time. Many of the proxies in marine sediments are well established and have revealed much about Antarctica's climate and environmental history, including long term cooling since 65 Ma, the initiation of glaciation in Antarctica 34 Ma, and ice sheet dynamics since then (Escutia et al., 2019; Naish et al., 2009; Noble et al., 2020; Florindo et al., 2022 and numerous others). Typically, oxygen isotope records contained within the calcium carbonate shells of benthic foraminifera are used as proxies for ice volume and deep ocean temperature (Zachos et al., 2001). Until recently, most marine sediment cores used to inform current understanding of Antarctic paleoclimate have been obtained from the deep sea, relatively distant from the Antarctic continental shelf (Shevenell & Bohaty, 2012) and in many cases have investigated deeper timescales than the Quaternary (Figure 2b).

There are however, marine sediments from the Antarctic continental margin that also contain a wealth of information about paleoenvironments, even though these records are not necessarily continuous past the LGM, since the advance of ice shelves can remove sediments as they expand across the continental margin. In addition, Antarctica sediments generally lack calcium carbonate shells (although see Scherer et al. (2007)) as they are not well preserved on the Antarctic continental margin (Gersonde et al., 2005). Together this has, in part, prevented detailed direct assessments of changes in the Antarctic ice sheet over time. Collection of marine sediment cores from the Antarctic margin is also logistically challenging. Development of technological advances in collecting cores from the shelf (e.g., ANtarctic DRILLing project [ANDRILL], (Scherer et al., 2007)), and recent International Ocean Discovery Program (IODP) expeditions (Escutia et al., 2019) have provided significant advances and enabled the investigation of biological proxies within these archives (Figure 3f). Continued developments in technology to drill under ice shelves will be important for developing biological paleo‐records from shelf sediments because one major limiting factor is obtaining sediment material from key locations (Hodgson, Bentley, et al., 2016; Koppers & Coggon, 2020).

Nonetheless, understanding past sensitivities of ice shelves is important to help constrain models for future climate change and a range of proxies from marine sediment cores are continuing to be developed for this endeavour. Ice shelves are floating sheets of ice permanently attached to the Antarctic land mass, and are important features for buttressing inland ice, thereby preventing the discharge of land ice into the sea, which contributes to sea level rise (Noble et al., 2020). Diatom and foraminifera assemblages and geochemistry can provide indicators of retreating ice shelves (seeSmith et al. (2019) for a review). Subglacial sediments obtained by drilling at various locations through the Ross Ice shelf and the West Antarctic Ice Sheet (WAIS) have detected diatoms that were deposited during Pleistocene interglacials. These, in conjunction with a combination of radiocarbon dating of organic carbon in subglacial sediments, ice‐penetrating radar and numerical ice sheet modelling, have indicated that during the Holocene the grounding line of the WAIS retreated several hundred kilometres inland before readvancing to its present position (Kingslake et al., 2018). This important finding indicates that the WAIS may be able to retreat far inland without leading to complete ice sheet collapse (Kingslake et al., 2018).

For over 40 years changes in microfossils of siliceous planktonic organisms, such as diatoms and radiolarians contained within marine sediments, have been commonly used paleoclimate proxies to investigate past sea ice extent, ocean temperatures and productivity, particularly at the LGM (see Gersonde et al. (2005) and within). Diatoms are phototrophic algae and live in the surface ocean, are responsible for ~70% of primary production in the Southern Ocean and accumulation of their silicious remains in sediments are one of the major sources of paleoclimate and paleoproductivity information in and around Antarctica (Tréguer et al., 1995). Diatom abundance and species assemblages quickly respond to changes in conditions and they have been used to reconstruct sea surface temperature, ocean circulation, and sea ice variability (Crosta et al., 2022; Panitz et al., 2015). Particular diatom species are sensitive to sea ice extent, so their presence or absence in a diatom assemblage, preserved down core, can indicate sea ice expansion or contraction (see Armand et al., 2005; Crosta et al., 2022; Smith et al., 2019). Similarly, the combination of diatom species present in sediment can be ‘translated’ to a temperature estimate using a transfer function, based on the known ranges and temperature sensitivity of the same species living today (Armand et al., 2005; Crosta et al., 2005; Romero et al., 2005).

An emerging advance in the use of diatom proxies is the development of diatom biomarkers to reconstruct sea ice variations through a combination of the diatom‐derived Ice Proxy of the Southern Ocean with 25 carbon atoms (IPSO25) with phytoplankton‐derived lipids (e.g., sterols, highly branched isoprenoid [HBI] ‐trienes; (Lamping et al., 2021; Vorrath et al., 2019)). The phytoplankton lipids are markers of open ocean conditions, which means the ratio of IPSO25 to phytoplankton biomarkers, the PIPSO25 index, shows the more subtle interplay between sea ice extent and ocean temperatures at decadal resolution, and points to subtle shifts in sea ice seasonality over the past 240 years (Vorrath et al., 2020). Additional advances in the use of diatom records include applying established techniques like δ^18^O analyses to diatom species or revealing sea ice changes on seasonal scales (Swann et al., 2013) or glacial ice discharge (Pike et al., 2013). Of significance is extracting the sea ice records from these established archives and methods in ice shelf areas that have previously not been accessed (Swann et al., 2013).

Other organic biomarkers have also been developed to use as paleoclimate proxies (Lamping et al., 2021). Pelagic marine single‐celled organisms from the domain Archaea, provide an alternative means of estimating past sea surface temperatures. These organisms, present within surface waters today, are also contained within marine sediments, and modify their membrane lipid composition in response to temperature. Therefore, by measuring the organic biomarker TEX_86_ (the tetraether index of tetraethers with 86 carbon atoms) in marine sediment cores, past sea surface temperatures can be estimated (Schouten et al., 2002). Application of this method to marine sediment cores collected from the Antarctic margin detected cooling of sea surface temperatures over the past 12 ka west of the Antarctic Peninsula, and highlighted the importance of regional drivers in determining sea ice change (Shevenell et al., 2011). Additional studies are required to fully characterize the relationship between sea surface temperature and TEX_86_ across different regions in the modern ocean to fully use this proxy to understand the Southern Ocean paleoenvironment (Pearson & Ingalls, 2013).

The use of DNA contained within marine sediments (*seda*DNA) in Antarctica is in its infancy (Armbrecht, 2020). The technique offers promise in understanding past ecosystems across a range of timescales as genetic material is preserved from not only ‘standard’ marine sediment proxies such as diatoms, foraminifera and other microfossils, but rather a wide variety of eukaryotes, prokaryotes, viruses, and archaea. Optimisation of primer pairs can enable focus on particular taxonomic groups of interest. A study investigating *seda*DNA using metabarcoding within Arctic sediment identified changes in biodiversity back to ~100 ka and related these to sea ice changes (De Schepper et al., 2019). Approaches targeting the abundance of a species of sea ice associated dinoflagellate (De Schepper et al., 2019) and sequence variants within a planktic foraminifera (Pawłowska et al., 2020) over ~100 and 140 ka time scales within Arctic cores, respectively, were able to link these to paleoenvironmental conditions. This demonstrates the potential for similar approaches to be applied in the Antarctic context. Significant challenges exist in investigating *seda*DNA due to the tiny amounts of fragmented and degraded DNA present within marine sediments, that are easily contaminated with modern DNA. In addition, application of a metagenomics approach to sequence many organisms simultaneously, is likely the future for these approaches, but their utility is constrained by the limited availability of reference databases (Table 2) (Armbrecht, 2020).

Additional biological proxies showing promise for improving knowledge of Antarctic climate and ecosystem dynamics in the past include δ^18^O of benthic foraminifera as a marker for sea ice related temperature changes, (where well preserved); (Lund et al., 2021) and the promise of extracting more from existing records, for example through the development reference datasets of Southern Ocean radiolarians (Lawler et al., 2021) from surface marine sediments. In addition, elemental analysis (XRF) is performed almost routinely now for marine sediments, and ratios are used to determine not only environmental change but also changes in biological productivity (Tables 1 and 2). Furthermore, multi‐proxy reconstructions between proxies in the same sediment cores and from comparisons of different proxy types that give complementary information, for example, sea ice reconstructions from ice cores and marine sediments (Thomas et al., 2019), and through the discovery of new Antarctic marine sediment biological proxies (Hartman et al., 2018) will improve knowledge of palaeoenvironments.

## 9 WHAT IS THE FUTURE FOR BIOLOGICAL ARCHIVES?

Biological archives have already made significant contributions to our understanding of Antarctic history. These include the demonstration of the existence of terrestrial glacial refugia (Kelley et al., 2014; Stevens & D'Haese, 2014) and shifts in penguin distribution in response to past climate change (e.g., Hu et al., 2013). Perhaps the most important insight from biological proxies has been the reconstruction of the paleotemperature curve from δ^18^O in foraminifera skeletons from distal marine sediment cores, which not only provided a valuable framework for historical ecology, but also definitive proof of the orbital variations proposed as climate drivers by Milanković (Emiliani, 1955; Urey, 1948). As new and novel biological archives and proxies are refined and developed, making the most of the information they provide is critical. As with most multi‐ or transdisciplinary approaches, the practical challenges of integrating varying data and scales are transcended only by the discipline‐specific language and understanding required to combine information (Bokade et al., 2021). Although difficult, clear guidance for achieving success in transdisciplinary work has now been developed (Lawrence et al., 2022; Pineo et al., 2021), and the benefits of such novel understanding outweigh the effort required, especially given the urgency of the climate crisis.

Not all advances need to be aimed at novel archives or methods; complementing emerging approaches with traditional ones can provide important progress. For example, generation times are critical estimates in calibrating genomic coalescent analyses, but direct life‐history studies on Antarctic or Southern Ocean animals are scarce because of the practical constraints of field work. Absolute dating methods, provided by sclerochronologies (see Benthic marine species), can be used for generation time proxies, which in turn can be applied with genomic proxies of ice sheet response and subsequent habitat availability. However, in the terrestrial realm, event dating may be complicated by species with long‐term cryptobiotic capabilities such as tardigrades and some mosses (Roads et al., 2014). In these unusual circumstances, the ‘shared’ history of populations may have become independent from the timing of actual gene flow and evolutionary history, but data to calibrate these are limited. Empirical derivations can nonetheless be made by incorporating approaches such as those provided by Dynamic Energy Budget theory to provide estimates of life‐history parameters (Kooijman et al., 2021).

A fundamental challenge for developing reconstructions using archives that accumulate in layers, such as peats, lake and marine shelf sediments, and animal colonies, is that they rely on an absence of mixing and reworking during and after deposition, minimal compaction during coring, and ideally collection of more than one core at a site or in the study area. To address this, strategic site selection, the appropriate equipment and subsequent accurate sub‐sampling, is necessary. For example, bathymetric profiling of lakes and seabed mapping prior to coring can aid determining the area with no, or at least minimal, disturbance. There are different types of peat deposits and animal colonies, which need to be understood when choosing where to core. Specific corers exist for different archives, and there are different devices for sub‐sampling cores depending on requirements (see De Vleeschouwer et al., 2010 and Last & Smol, 2001 for comprehensive reviews and recommendations on the collection of stratigraphic archives). Modelling of these ‘encoding’ and ‘archiving’ uncertainties in marine and lake sediments can help to minimize their impact (Dolman & Laepple, 2018). Despite these challenges, valuable reconstructions are developed, and there are increasing efforts to integrate records.

Synchronising proxy records to a common timescale, for example, circumventing radiocarbon marine reservoir effects, will also help maximize the use of archives and their proxies (Waelbroeck et al., 2019). Methods such as proxy system modelling are emerging to better quantify proxy reconstructions and associated uncertainties (Evans et al., 2013). These aim to extract a broader range of parameters from proxy data and could prove an important advance for understanding multiple factors contributing to ecosystem change (Evans et al., 2013). Data assimilation techniques extend this approach by combining proxy data with climate model output to produce gridded products with increased spatial coverage and number of climate parameters than those contained in individual proxy records (Franke et al., 2017; Tardif et al., 2019).

Integrating multiple proxies across a single sample or site can help provide deeper insights into paleoclimates and paleoecological responses than the use of individual proxies alone. For example, multiple studies have integrated *sed*aDNA findings with other paleoecological proxy data to provide additional validation and/or contextualization (Crump et al., 2019; Mitchell & Rawlence, 2021). Having multiple, independent data comparisons gives a richer understanding of the ecosystem as a whole, and can pinpoint the timing and likely causation for extinction events long passed (Graham et al., 2016). In the Southern Ocean system, this type of integration could be very powerful. However, despite extensive integration of marine sediment and ice core data to reconstruct sea ice, Thomas et al. (2019) identified just two marine records that had comparable sample resolution and age‐scale to that of ice core records. However, importantly they identified where re‐sampling priorities might address these shortfalls, and thus achieve a multi‐proxy reconstruction. The wealth of emerging proxies highlighted here shows the power of integration across techniques, localities, and timescales, and will help to create a richer reconstructions.

Recent methods enabling analysis of whole genomes enable greater power and application for detecting past demographic change. Much of this innovation has been driven by advances in human genomics; it is clear that analyses of whole genomes sequenced from extant terrestrial species and benthic marine invertebrates, based on the sequentially Markovian coalescent (Mather et al., 2020), permit much finer scale timing of historic gene flow, including migration directionality (Leitwein et al., 2020). Analyses of whole genomes also enable fine scale investigation of past population sizes (e.g., bottlenecks and expansions), and provide a dated indication of changes in habitat availability over time. Genomic methods can now date the emergence of specific alleles (Smith et al., 2018), which enable insight into the functional basis of a species' response to environmental change. This has relevance for understanding species' abilities to adapt to rapidly changing ocean conditions related to ice sheet change, such as salinity and temperature.

To enhance the reusability of data, and therefore the potential integration of novel multi‐proxy datasets, FAIR data principles should be used (Wilkinson et al., 2016). These guidelines follow principles of findability, accessibility, interoperability and reusability, and support the continuing use of digital assets. As science increasingly relies on computer‐assisted data gathering, it is clear that we need to future proof the accessibility of data being gathered now. Some of this durability is provided by making sure data have unique digital identifiers, that the associated metadata are well‐described, meet community standards, and that the usage licence is clear. International working groups have developed protocols governing data gathering, sharing and harmonisation prior to the formalisation of the FAIR principles (Kucera et al., 2005). For established archives, this has resulted in more comprehensive reconstructions (Gersonde et al., 2005; Kaufman et al., 2020; PAGES 2k Consortium et al., 2019) than would occur otherwise. Oftentimes, it is the attempts to integrate multi‐proxy datasets that drives the establishment of standardized vocabularies, which enhance both interaction and discoverability (Morrill et al., 2021). In the light of FAIR principles, reporting standards are now being revisited, in some cases using crowd‐sourced methods (e.g., PaCTS 1.0, (Paleoclimate Community reporting Standard) Khider et al., 2019). Strategic communication around standards for emerging archives will enhance the utility and effectiveness of their potential uses.

Museum collections have and will continue to provide invaluable samples for paleoclimate reconstructions (e.g., Crumsey et al., 2019; Fraser et al., 2021; Moritz et al., 2008), but many Antarctic samples are not housed nationally and instead are spread across diffuse collections. In these cases, digital and physical Research Infrastructure's (RIs) are critical for researchers to gather the needed resources efficiently and can provide formal ways of addressing, defining, and managing standard protocols. Many of the new approaches outlined in this review can benefit from museum collections to access the geographic and time span of samples needed to address critical research questions (Meineke et al., 2018). The support provided by RIs not only enhances common language among disciplines but fosters collaboration and enhances data sharing. Integration among RIs themselves can reciprocally inform across fields and improve capabilities to anticipate the impacts of global change and biodiversity losses (Nieto‐Lugilde et al., 2021).

There is no doubt that intermediate steps will be needed to bring together disparate archives. Cross‐validation techniques will also be critical. Increasing the potential for overlap may stem from approaches that use multiple proxies from the same biological archive, or bring together independent archives through a common proxy, and these may be driven by technological developments. Transdisciplinary research is fundamental to making progress on wide‐ranging questions, and, by definition, biological archives span the biological, geological, and physical sciences. Looking at a broader range of archives and proxies will help assess the implications of ecological and molecular change for contemporary ecosystem functioning (Fordham et al 2020), and improve our ability to predict responses and adaptation to climate change and environmental stressors. Our challenge to the scientific community is to further unlock the potential of biological archives and proxies for an integrated understanding of past ecosystems and environments, alongside past climate approximations, in the unique Antarctic and sub‐Antarctic regions.

## AUTHOR CONTRIBUTIONS

JS conceived the idea for the paper, which was refined and improved with contributions from all authors. SL prepared Figures 1 and 3. All authors reviewed the literature and contributed to the tables. JS, HM, KS, NW, SC, SR, and KM contributed to the first draft of the manuscript. All authors contributed to subsequent drafts.

## CONFLICT OF INTEREST

The authors have no conflict of interest to declare.

## Supporting information


Data S1

## Data Availability

Data sharing is not applicable to this article as no new data were created or analyzed in this study.
