# Supplementary material for: Emerging biological archives can reveal ecological and climatic change in Antarctica
Source: Glob Chang Biol. 2022 Sep 8;28(22):6483–508. doi: 10.1111/gcb.16356 (PMC9826052; doi:10.1111/gcb.16356)
Supplement: Supplementary file 1 — Data S1 [file GCB-28-6483-s001.docx]

**Supplementary information 1**

Supplementary Figure 1. Map of Antarctica and the Southern Ocean. Studies investigating biological archives of paleoenvironments and paleoclimates are indicated, including (A) Marine shelf sediments, (B) Benthic marine species, (C) Terrestrial invertebrates, (D) Animal colonies, (E) Live mosses/peat, and (F) Lake sediments. Labels correspond to the reference ID of each datapoint in Supplementary Table 1-6. Ice-free areas (brown areas on the Antarctic continent) are shown following Antarctic Conservation Biogeographic Regions (ACBRs) in [(Terauds & Lee, 2016)](https://paperpile.com/c/5NLom5/pWk7) and are important locations for moss beds, terrestrial invertebrates and many animal colonies.

**
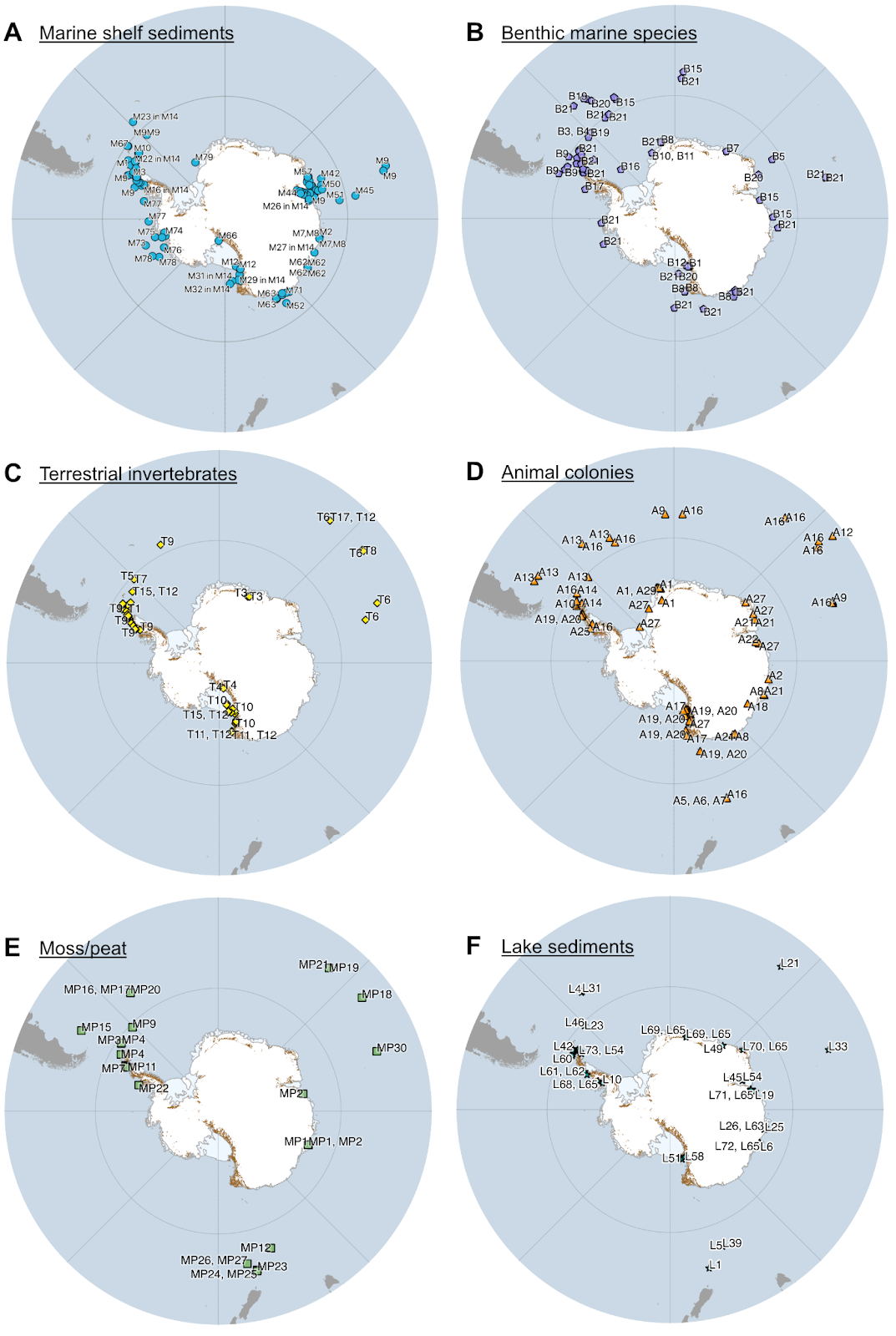
**

Supplementary Table 1. Locality and reference of existing studies investigating marine shelf sediment as biological archives with a Quaternary focus.

| **Locality** | **Reference** | **Reference ID** |
| --- | --- | --- |
| South Shetland Islands, King George Island, Maxwell Bay | Monien et al (2011) | M1 |
| East Antarctica, Bunger Oasis | Kulbe et al. (2001) | M2 |
| Antarctic Peninsula, ODP Site 1098 | Shevenell et al. (2011) | M3 |
| Antarctic Peninsula, Palmer Deep | Domack (2002) in Bentley et al. (2009) | M4, M5 |
| Antarctic Peninsula, Andvord Drift | Domack et al. (2003) in Bentley et al. (2009) | M6, M5 |
| Bunger Hills, Izvilistaja Inlet, Core 6045 | Melles et al. (1994) in Ingólfsson et al. (1998) | M7, M8 |
| Rybiy Khvost Bay, Bunger Hills, Rybiy Khvost    Bay, Core 6019 | Melles et al. (1994) in Ingólfsson et al. (1998) | M7, M8 |
| IODP, Leg 113, Site 696, Hole A | International Ocean Discovery Program (IODP) (2015) | M9 |
| IODP, Leg 113, Site 696, Hole B | International Ocean Discovery Program (IODP) (2015) | M9 |
| IODP, Leg 119, Site 736, Hole A | International Ocean Discovery Program (IODP) (2015) | M9 |
| IODP, Leg 119, Site 736, Hole B | International Ocean Discovery Program (IODP) (2015) | M9 |
| IODP, Leg 119, Site 736, Hole C | International Ocean Discovery Program (IODP) (2015) | M9 |
| IODP, Leg 119, Site 737, Hole A | International Ocean Discovery Program (IODP) (2015) | M9 |
| IODP, Leg 119, Site 737, Hole B | International Ocean Discovery Program (IODP) (2015) | M9 |
| IODP, Leg 119, Site 739, Hole A | International Ocean Discovery Program (IODP) (2015) | M9 |
| IODP, Leg 119, Site 739, Hole B | International Ocean Discovery Program (IODP) (2015) | M9 |
| IODP, Leg 119, Site 739, Hole C | International Ocean Discovery Program (IODP) (2015) | M9 |
| IODP, Leg 119, Site 740, Hole A | International Ocean Discovery Program (IODP) (2015) | M9 |
| IODP, Leg 119, Site 740, Hole B | International Ocean Discovery Program (IODP) (2015) | M9 |
| IODP, Leg 119, Site 741, Hole A | International Ocean Discovery Program (IODP) (2015) | M9 |
| IODP, Leg 119, Site 742, Hole A | International Ocean Discovery Program (IODP) (2015) | M9 |
| IODP, Leg 178, Site 1097, Hole A | International Ocean Discovery Program (IODP)  (2015) | M9 |
| IODP, Leg 178, Site 1100, Hole A | International Ocean Discovery Program (IODP) (2015) | M9 |
| IODP, Leg 178, Site 1100, Hole B | International Ocean Discovery Program (IODP) (2015) | M9 |
| IODP, Leg 178, Site 1100, Hole C | International Ocean Discovery Program (IODP) (2015) | M9 |
| IODP, Leg 178, Site 1100, Hole D | International Ocean Discovery Program (IODP) (2015) | M9 |
| IODP, Leg 178, Site 1102, Hole A | International Ocean Discovery Program (IODP) (2015) | M9 |
| IODP, Leg 178, Site 1102, Hole B | International Ocean Discovery Program (IODP) (2015) | M9 |
| IODP, Leg 178, Site 1102, Hole C | International Ocean Discovery Program (IODP) (2015) | M9 |
| IODP, Leg 178, Site 1102, Hole D | International Ocean Discovery Program (IODP) (2015) | M9 |
| IODP, Leg 178, Site 1103, Hole A | International Ocean Discovery Program (IODP) (2015) | M9 |
| IODP, Leg 188, Site 1166, Hole A | International Ocean Discovery Program (IODP) (2015) | M9 |
| IODP, Leg 188, Site 1166, Hole B | International Ocean Discovery Program (IODP) (2015) | M9 |
| SHALDRILL II, Hole 3C | Anderson et al. (2011) | M10 |
| SHALDRILL II, Hole 12A | Anderson et al. (2011) | M10 |
| SHALDRILL II, Hole 5C | Anderson et al. (2011) | M10 |
| SHALDRILL II, Hole 5D | Anderson et al. (2011) | M10 |
| SHALDRILL II, Hole 6C | Anderson et al. (2011) | M10 |
| SHALDRILL II, Hole 6D | Anderson et al. (2011) | M10 |
| Cape Roberts Project (CRP), CRP-3 | Dunbar et al. (2008) | M11 |
| Cape Roberts Project (CRP), CRP-2/2A | Dunbar et al. (2008) | M11 |
| Cape Roberts Project (CRP), CRP-2/1 | Dunbar et al. (2008) | M11 |
| MSSTS-1 | Ehrmann (1998) | M12 |
| CIROS-1 | Ehrmann (1998) | M12 |
| ANDRILL, AND-1B | Naish et al. (2009) | M13 |
| TPC522 | Peck et al. (2015) in Crosta et al. (2022) | M15 in M14 |
| NBP0201-JPC43 | Allen et al. (2010) in Crosta et al. (2022) | M16 in M14 |
| PD92-II-01 GC1 | Taylor et al. (2001) in Crosta et al. (2022) | M17 in M14 |
| ODP1089 | Sjunneskog & Taylor (2002) in Crosta et al. (2022) | M18 in M14 |
| NBP9903-JPC10 | Etourneau et al. (2013) in Crosta et al. (2022) | M19 in M14 |
| NBP0201-PC61 | Heroy et al. (2008) in Crosta et al. (2022) | M20 in M14 |
| NBP0502-KC2 | Minzoni et al. (2015) in Crosta et al. (2022) | M21 in M14 |
| NBP0003-JPC38 | Barbara et al. (2016) in Crosta et al. (2022) | M22 in M14 |
| PS67/206-1 | Xiao et al. (2016) in Crosta et al. (2022) | M23 in M14 |
| KROCK/15/GC29 | Taylor & McMinn (2002) in Crosta et al. (2022) | M24 in M14 |
| NBP0101-JPC24 | Denis et al. (2010) in Crosta et al. (2022) | M25 in M14 |
| Co1011 | Berg et al. (2010) in Crosta et al. (2022) | M26 in M14 |
| Co1010 | Berg et al. (2010) in Crosta et al. (2022) | M26 in M14 |
| PG1433 | Cremer et al. (2003) in Crosta et al. (2022) | M27 in M14 |
| MD03-2601 | Crosta et al. (2007) in Crosta et al.  (2022) | M28 in M14 |
| NBP9501-31 | Cunningham et al. (1999) in Crosta et al. (2002) | M29 in M14 |
| BAY05-43c | Mezgec et al. (2017) in Crosta et al. (2022) | M30 in M14 |
| NBP9501-37 | Cunningham et al. (1999) in Crosta et al. (2002) | M31 in M14 |
| NBP9501-39 | Cunningham et al. (1999) in Crosta et al.  (2002) | M31 in M14 |
| ANTA99-cJ5 | Mezgec et al. (2017) in Crosta et al. (2022) | M32 in M14 |
| ODP Site 740 | Domack et al. (1991) | M33 |
| ODP Site 741 | Whitehead et al. (2006) | M34 |
| GC5 | Rathburn et al. (1994) | M35 |
| GC35 | Rathburn et al. (1994) | M35 |
| AM02 | Hemer & Harris (2003) | M36 |
| GC1 | Taylor & McMinn (2001) | M37 |
| GC29 | Taylor & McMinn (2001) | M38 |
| KC29B | Taylor & Leventer (2003) | M39 |
| KC30B | Taylor & Leventer (2003) | M39 |
| KC31 | Taylor & Leventer (2003) | M39 |
| KC32 | Taylor & Leventer (2003) | M39 |
| 186 GC22 | Domack et al. (1998) | M40 |
| KROCK/GC22 | Domack et al. (1998) | M40 |
| ODP Site 1167 | Cooper & O’Brien (2004) | M41 |
| ODP Site 1165 | Whitehead & Bohaty (2003) | M42 |
| ODP Site 1166 | Whitehead & Bohaty (2003) | M42 |
| ODP Site 742A | Mahood & Barron (1996) | M43 |
| Bardin Bluffs Formation, Amery Oasis | Whitehead & Mckelvey (2001) | M44 |
| ODP Site 751 | Bohaty & Harwood (1998) | M45 |
| AA186-GC34 | Sedwick et al. (2001) | M46 |
| KROCK-GC1 | Sedwick et al. (2001) | M46 |
| AA149-GC2 | Sedwick et al. (2001) | M46 |
| KROCK-GC2 | Sedwick et al. (2001) | M46 |
| AA149-GC1 | Sedwick et al. (2001) | M46 |
| ANT30/P1-03 | Wu et al. (2017) | M47 |
| ANT29/P4-01 | Wu et al. (2017) | M47 |
| ANT30/P1-02 | Tang et al. (2016) | M48 |
| 901/GC18 | O'Brien & Harris (1996) | M49 |
| 901/GC21 | O'Brien & Harris (1996) | M49 |
| 901/GC24 | O'Brien & Harris (1996) | M49 |
| 901/GC27 | O'Brien & Harris (1996) | M49 |
| ANT29/P5-03 | Wu et al. (2021) | M50 |
| ODP Site 744 | Ehrmann (1991) | M51 |
| IODP U1361A | Wilson et al. (2018) | M52 |
| AM01b | Post et al. (2014) | M53 |
| AM02 | Post et al. (2014) | M53 |
| AM03 | Post et al. (2014) | M53 |
| AM04 | Post et al. (2014) | M53 |
| AM05 | Post et al. (2014) | M53 |
| AM06 | Post et al. (2014) | M53 |
| JPC 43B | Stickley et al. (2005), Mackintosh et al. (2011), Leventer et al. (2006) | M54, M55, M56 |
| JPC40 | Mackintosh et al. (2011), Leventer et al. (2006) | M55, M56 |
| JPC24 | Leventer et al. (2006) | M56 |
| JPC11 | Leventer et al. (2006) | M56 |
| JPC41 | Alley et al. (2018) | M57 |
| PD92-30 | Leventer et al. (1996) | M58 |
| KC23 | Brachfeld et a. (2003) | M59 |
| KC208.09 | Leventer et al. (1993) | M60 |
| DF88 WG17 | Leventer et al. (1993) | M60 |
| DF89 WG35 | Leventer et al. (1993) | M60 |
| NBP9903-KC1 | Domack et al. (2003) | M6 |
| NBP9903-KC3 | Domack et al. (2003) | M6 |
| NBP9903-18B | Domack et al. (2003) | M6 |
| NBP9903-18C | Domack et al. (2003) | M6 |
| NBP9903-JPC18 | Domack et al. (2003) | M6 |
| NBP9903-JPC28 | Domack et al. (2003) | M6 |
| PS1599-3 | Huang et al. (2020) | M61 |
| NBP1402-JPC30 | Gulick et al. (2017) | M62 |
| NBP1402-JPC31 | Gulick et al. (2017) | M62 |
| NBP1402-JPC55 | Gulick et al. (2017) | M62 |
| NBP1402-JPC54 | Gulick et al. (2017) | M62 |
| NBP0101-KC-1 | McMullen et al. (2006) | M63 |
| NBP0101-KC-2 | McMullen et al. (2006) | M63 |
| NBP0101-KC-12 | McMullen et al. (2006) | M63 |
| NBP0101-KC-13 | McMullen et al. (2006) | M63 |
| NBP0101-JPC10 | Maddison et al. (2006) | M64 |
| NBP1001-KC-54 | Christ et al. (2015) | M65 |
| NBP1001-KC-55 | Christ et al. (2015) | M65 |
| JPC-126 | Christ et al. (2015) | M65 |
| WGZ | Venturelli et al. (2020) | M66 |
| TG-01 | López-Quirós et al. (2021) | M67 |
| TG-03 | López-Quirós et al. (2021) | M67 |
| PS69/849-2 | Borchers et al. (2016) | M68 |
| IODP U1357 | Ashley et al. (2021b), Behrens et al. (2022) | M69, M70 |
| DTGC2011 | Ashley et al. (2021a) | M71 |
| CB2010 | Campagne et al. (2015) | M72 |
| PS58/254 | Konfirst et al. (2012) | M73 |
| PS75/160 | Hillenbrand et al. (2013) | M74 |
| PS75/167 | Hillenbrand et al. (2013) | M74 |
| PS75/214 | Hillenbrand et al. (2013) | M74 |
| PS75/215 | Hillenbrand et al. (2017) | M75 |
| PS69/251 | Hillenbrand et al. (2017) | M75 |
| ANA08B/33 | Kim et al. (2021) | M76 |
| Eastern Bellingshausen Sea | Hillenbrand et al. (2003) | M77 |
| Western Bellingshausen Sea | Hillenbrand et al. (2003) | M77 |
| Amundsen Sea | Hillenbrand et al. (2003) | M77 |
| PS2547 | Hillenbrand et al. (2002) | M78 |
| PS2551 | Hillenbrand et al. (2002) | M78 |

Supplementary Table 2. Locality and reference of existing studies investigating benthic marine species as biological archives with a Quaternary focus.

| **Species** | **Locality** | **Reference** | **Reference ID** |
| --- | --- | --- | --- |
| *Adamussium colbecki* (bivalve) | Ross Sea, McMurdo Sound, Explorers Cove | Cronin et al. (2020) | B1 |
| *Adamussium colbecki* (bivalve) | Ross Sea, McMurdo Sound, Bay of Sails | Cronin et al. (2020) | B2 |
| *Aequiyoldia eightsii* (bivalve) | South Orkney Islands | Román-González et al. (2017) | B3, B4 |
| *Laternula elliptica* (bivalve) | South Shetland Islands, King George Island | Brey et al. (2011) | B5 |
| *Laternula elliptica* (bivalve) | Ross Sea, McMurdo sound | Wing et al. (2020) | B6 |
| *Laternula elliptica* (bivalve) | Queen Maud Land, Lutzow-Holm Bay | Tada et al. (2006) | B7 |
| *Errina* spp. (coral) | Ross Sea, Adare Basin (1) | King et al. (2018) | B8 |
| *Errina* spp. (coral) | Ross Sea, Adare Basin (2) | King et al. (2018) | B8 |
| *Errina* spp. (coral) | Ross Sea, Adare Basin (3) | King et al. (2018) | B8 |
| *Errina* spp. (coral) | Ross Sea, Adare Basin (4) | King et al. (2018) | B8 |
| *Errina* spp. (coral) | Ross Sea, Adare Basin (5) | King et al. (2018) | B8 |
| *Errina* spp. (coral) | Weddell Sea, Larsen A | King et al. (2018) | B8 |
| *Errina* spp. (coral) | Wilkes Land, Mertz-Ninnis trough (1) | King et al. (2018) | B8 |
| *Errina* spp. (coral) | Wilkes Land, Mertz-Ninnis trough (2) | King et al. (2018) | B8 |
| *Desmophyllum dianthus*, *Caryophyllia* spp., *Paraconotrochus antarcticus* (coral) | Drake Passage, Sars seamount | Wilson et al. (2020) | B9 |
| *Desmophyllum dianthus*, *Caryophyllia* spp., *Paraconotrochus antarcticus* (coral) | Drake Passage, Interim seamount | Wilson et al. (2020) | B9 |
| *Desmophyllum dianthus*, *Caryophyllia* spp., *Paraconotrochus antarcticus* (coral) | Drake Passage, Shakleton fracture zone | Wilson et al. (2020) | B9 |
| *Cellaria incula* (Bryozoa) | Weddell Sea | Brey et al. (1999) in Roman-Gonzalez (2021) | B10, B11 |
| *Cellarinella nutti* (Bryozoa) | Ross Sea | Smith (2007) | B12 |
| *Cellarinella nodulata* (Bryozoa) | Ross Sea | Smith (2007) | B12 |
| *Swanomia belgica* (Bryozoa) | Ross Sea | Smith (2007) | B12 |
| Four species of Trematominae | Ross Sea | Janko et al. (2007) | B13 |
| Four species of Trematominae | Adélie Land | Janko et al. (2007) | B13 |
| Four species of Trematominae | South Shetland Islands | Janko et al. (2007) | B13 |
| *Trematomus bernacchii* (fish) | Ross Sea, McMurdo Sound | McMullin et al. (2017) | B14 |
| *Trematomus pennellii* (fish) | Ross Sea, McMurdo Sound | McMullin et al. (2017) | B14 |
| *Pagothenia borchgrevinki* (fish) | Ross Sea, McMurdo Sound | McMullin et al. (2017) | B14 |
| *Nymphon australe* (Pyconogonida) | Antarctic Peninsula | Soler-Membrives et al. (2017) | B15 |
| *Nymphon australe* (Pyconogonida) | Terra Adelie | Soler-Membrives et al. (2017) | B15 |
| *Nymphon australe* (Pyconogonida) | Ross Sea | Soler-Membrives et al. (2017) | B15 |
| *Nymphon australe* (Pyconogonida) | Davis Station | Soler-Membrives et al. (2017) | B15 |
| *Nymphon australe* (Pyconogonida) | Bruce Rise | Soler-Membrives et al. (2017) | B15 |
| *Nymphon australe* (Pyconogonida) | Bouvet Is. | Soler-Membrives et al. (2017) | B15 |
| *Nymphon australe* (Pyconogonida) | South Sandwich Iss. | Soler-Membrives et al. (2017) | B15 |
| *Nymphon australe* (Pyconogonida) | Weddell Sea | Soler-Membrives et al. (2017) | B15 |
| *Sterechinus neumayeri* (Echinodermata) | Antarctic Peninsula | Díaz et al. (2018) | B16 |
| *Sterechinus neumayeri* (Echinodermata) | Weddell Sea | Díaz et al. (2018) | B16 |
| *Sterechinus neumayeri* (Echinodermata) | Adelie Land | Díaz et al. (2018) | B16 |
| *Paralomis birsteini* (King crab) | Antarctic Peninsula, Marguerite Bay | Hellberg et al. (2019) | B17 |
| *Limacina antarctica* (Lithodidae) | Drake Passage | Sromek et al 2015 | B18 |
| *Nacella concinna* (Gastropoda) | South Georgia | González-Wevar et al. (2016) | B19 |
| *Nacella concinna* (Gastropoda) | South Orkney Islands | González-Wevar et al. (2016) | B19 |
| *Nacella concinna* (Gastropoda) | South Shetland Islands | González-Wevar et al. (2016) | B19 |
| *Nacella concinna* (Gastropoda) | Antarctic Peninsula | González-Wevar et al. (2016) | B19 |
| *Pareledone turqueti* (Cephalopoda) | Shag Rocks | Strugnell et al. (2012) | B20 |
| *Pareledone turqueti* (Cephalopoda) | South Georgia | Strugnell et al. (2012) | B20 |
| *Pareledone turqueti* (Cephalopoda) | South Sandwich Islands | Strugnell et al. (2012) | B20 |
| *Pareledone turqueti* (Cephalopoda) | Elephant Island | Strugnell et al. (2012) | B20 |
| *Pareledone turqueti* (Cephalopoda) | King George Island | Strugnell et al. (2012) | B20 |
| *Pareledone turqueti* (Cephalopoda) | Livingstone Island | Strugnell et al. (2012) | B20 |
| *Pareledone turqueti* (Cephalopoda) | Antarctic Peninsula | Strugnell et al. (2012) | B20 |
| *Pareledone turqueti* (Cephalopoda) | East Weddell Sea | Strugnell et al. (2012) | B20 |
| *Pareledone turqueti* (Cephalopoda) | Prydz Bay | Strugnell et al. (2012) | B20 |
| *Pareledone turqueti* (Cephalopoda) | West Ross Sea | Strugnell et al. (2012) | B20 |
| *Pareledone turqueti* (Cephalopoda) | Adélie Land | Strugnell et al. (2012) | B20 |
| *Ophionotus victoriae* (Ophuroidea) | Adélie Land | Lau et al. (2021) | B21 |
| *Ophionotus victoriae* (Ophuroidea) | Amundsen Sea | Lau et al. (2021) | B21 |
| *Ophionotus victoriae* (Ophuroidea) | Balleny Islands | Lau et al. (2021) | B21 |
| *Ophionotus victoriae* (Ophuroidea) | Bellingshausen Sea | Lau et al. (2021) | B21 |
| *Ophionotus victoriae* (Ophuroidea) | Bouvet Island | Lau et al. (2021) | B21 |
| *Ophionotus victoriae* (Ophuroidea) | Bransfield Strait | Lau et al. (2021) | B21 |
| *Ophionotus victoriae* (Ophuroidea) | Davis Sea | Lau et al. (2021) | B21 |
| *Ophionotus victoriae* (Ophuroidea) | Discovery Bank | Lau et al. (2021) | B21 |
| *Ophionotus victoriae* (Ophuroidea) | Elephant Island | Lau et al. (2021) | B21 |
| *Ophionotus victoriae* (Ophuroidea) | Heard Island | Lau et al. (2021) | B21 |
| *Ophionotus victoriae* (Ophuroidea) | Herdman Bank | Lau et al. (2021) | B21 |
| *Ophionotus victoriae* (Ophuroidea) | Larsen Ice Shelf | Lau et al. (2021) | B21 |
| *Ophionotus victoriae* (Ophuroidea) | Prydz Bay | Lau et al. (2021) | B21 |
| *Ophionotus victoriae* (Ophuroidea) | Ross Sea | Lau et al. (2021) | B21 |
| *Ophionotus victoriae* (Ophuroidea) | Scott Island | Lau et al. (2021) | B21 |
| *Ophionotus victoriae* (Ophuroidea) | South Shetland Islands | Lau et al. (2021) | B21 |
| *Ophionotus victoriae* (Ophuroidea) | South Georgia | Lau et al. (2021) | B21 |
| *Ophionotus victoriae* (Ophuroidea) | South Orkney Islands | Lau et al. (2021) | B21 |
| *Ophionotus victoriae* (Ophuroidea) | South Sandwich Islands | Lau et al. (2021) | B21 |
| *Ophionotus victoriae* (Ophuroidea) | Weddell Sea | Lau et al. (2021) | B21 |
| *Ophionotus victoriae* (Ophuroidea) | West Antarctic Peninsula | Lau et al. (2021) | B21 |
| *Ophionotus hexactis* (Ophuroidea) | Bransfield Strait | Lau et al. (2021) | B21 |
| *Ophionotus hexactis* (Ophuroidea) | Heard Island | Lau et al. (2021) | B21 |
| *Ophionotus hexactis* (Ophuroidea) | Larsen Ice Shelves | Lau et al. (2021) | B21 |
| *Ophionotus hexactis* (Ophuroidea) | Shag Rocks | Lau et al. (2021) | B21 |
| *Ophionotus hexactis* (Ophuroidea) | South Georgia | Lau et al. (2021) | B21 |

Supplementary Table 3. Locality and reference of existing studies investigating terrestrial invertebrates as biological archives with a Quaternary focus.

| **Species** | **Locality** | **Reference** | **Reference ID** |
| --- | --- | --- | --- |
| *Belgica antarctica* (Chironomidae) | South Shetland Islands | Allegrucci et al. (2012) | T1 |
| *Belgica antarctica* (Chironomidae) | Antarctic Peninsula (1) | Allegrucci et al. (2012) | T1 |
| *Belgica antarctica* (Chironomidae) | Antarctic Peninsula (2) | Allegrucci et al. (2012) | T1 |
| *Belgica antarctica* (Chironomidae) | Antarctic Peninsula, Palmer Station | Kelley et al. (2014) | T2 |
| *Cryptopygus sverdrupi* (Collembola) | Dronning Maud Land, Nunatack Utsteinen | Stevens & D’Haese (2014) | T3 |
| *Cryptopygus sverdrupi* (Collembola) | Dronning Maud Land, Nunatack Pinguinane | Stevens & D’Haese (2014) | T3 |
| *Cryptopygus sverdrupi* (Collembola) | Dronning Maud Land, Tanngarden | Stevens & D’Haese (2014) | T3 |
| *Cryptopygus sverdrupi* (Collembola) | Dronning Maud Land, Duboisbreen | Stevens & D’Haese (2014) | T3 |
| *Cryptopygus sverdrupi* (Collembola) | Dronning Maud Land, Vengen | Stevens & D’Haese (2014) | T3 |
| *Cryptopygus sverdrupi* (Collembola) | Dronning Maud Land, Brattnipane | Stevens & D’Haese (2014) | T3 |
| Six species of Collembola | Ross Sea, Queen Maud Mountains | Collins et al. (2020) | T4 |
| Six species of Collembola | Ross Sea, Queen Alexandra Range | Collins et al. (2020) | T4 |
| Six species of Collembola | Ross Sea, southern Victoria Land | Collins et al. (2020) | T4 |
| *Boeckella poppei* (Copepoda) | South Orkney Islands, Signy Island | Maturana et al. (2020) | T5 |
| Ectemnorhinini | Prince Edward Islands | Baird et al. (2021) | T6 |
| Ectemnorhinini | Crozet Islands | Baird et al. (2021) | T6 |
| Ectemnorhinini | Kerguelen Islands | Baird et al. (2021) | T6 |
| Ectemnorhinini | Heard Island | Baird et al. (2021) | T6 |
| Mites | South Orkney Islands, Signy Island | Hodgson & Convey (2005) | T7 |
| *Pachnobium dreuxi* (Ectemnorrhininae) | Crozet Islands | Chapelin-Viscardi et al. (2010) | T8 |
| *Cryptopygus* sp. (springtail) | Antarctic Peninsula, Bernardo O'Higgins | McGaughran et al. (2019) | T9 |
| *Cryptopygus* sp. (springtail) | Robert Island, Coppermine Peninsula | McGaughran et al. (2019) | T9 |
| *Cryptopygus* sp. (springtail) | Deception Island | McGaughran et al. (2019) | T9 |
| *Cryptopygus* sp. (springtail) | Livingston Island, Byers Peninsula | McGaughran et al. (2019) | T9 |
| *Cryptopygus* sp. (springtail) | Livingston Island, Hannah Point | McGaughran et al. (2019) | T9 |
| *Cryptopygus* sp. (springtail) | Nelson Island, Harmony Point | McGaughran et al. (2019) | T9 |
| *Cryptopygus* sp. (springtail) | Paulet Island | McGaughran et al. (2019) | T9 |
| *Cryptopygus* sp. (springtail) | King George Island, Potter Cove | McGaughran et al. (2019) | T9 |
| *Cryptopygus* sp. (springtail) | Berthelot Island | McGaughran et al. (2019) | T9 |
| *Cryptopygus* sp. (springtail) | East Coast Lemaire | McGaughran et al. (2019) | T9 |
| *Cryptopygus* sp. (springtail) | Grand Island | McGaughran et al. (2019) | T9 |
| *Cryptopygus* sp. (springtail) | Paradise Harbour | McGaughran et al. (2019) | T9 |
| *Cryptopygus* sp. (springtail) | Petermann Island | McGaughran et al. (2019) | T9 |
| *Cryptopygus* sp. (springtail) | Port Lockroy (Gouider Island) | McGaughran et al. (2019) | T9 |
| *Cryptopygus* sp. (springtail) | Prospect Point (Graham Land) | McGaughran et al. (2019) | T9 |
| *Cryptopygus* sp. (springtail) | Spert Island | McGaughran et al. (2019) | T9 |
| *Cryptopygus* sp. (springtail) | Terrada Point | McGaughran et al. (2019) | T9 |
| *Cryptopygus* sp. (springtail) | Alamode Island | McGaughran et al. (2019) | T9 |
| *Cryptopygus* sp. (springtail) | Anchorage | McGaughran et al. (2019) | T9 |
| *Cryptopygus* sp. (springtail) | Detaille Island | McGaughran et al. (2019) | T9 |
| *Cryptopygus* sp. (springtail) | Evensen Point | McGaughran et al. (2019) | T9 |
| *Cryptopygus* sp. (springtail) | Jenny Island | McGaughran et al. (2019) | T9 |
| *Cryptopygus* sp. (springtail) | Killingbeck Island | McGaughran et al. (2019) | T9 |
| *Cryptopygus* sp. (springtail) | Leonie Island | McGaughran et al. (2019) | T9 |
| *Cryptopygus* sp. (springtail) | Adelaide Island, Mackay Point | McGaughran et al. (2019) | T9 |
| *Cryptopygus* sp. (springtail) | Adelaide Island, Reptile Ridge | McGaughran et al. (2019) | T9 |
| *Cryptopygus* sp. (springtail) | Adelaide Island, Rothera | McGaughran et al. (2019) | T9 |
| *Cryptopygus* sp. (springtail) | South Sandwich Islands, Saunders Island | McGaughran et al. (2019) | T9 |
| *Acutuncus antarcticus* (tardigrade) | Ross Sea, Cape Harlett | Cesari et al. (2016) | T10 |
| *Acutuncus antarcticus* (tardigrade) | Ross Sea, Crater Cirque | Cesari et al. (2016) | T10 |
| *Acutuncus antarcticus* (tardigrade) | Ross Sea, Edmonson Point | Cesari et al. (2016) | T10 |
| *Acutuncus antarcticus* (tardigrade) | Ross Sea, Terranova Bay | Cesari et al. (2016) | T10 |
| *Acutuncus antarcticus* (tardigrade) | Ross Sea, Inexpressible Island | Cesari et al. (2016) | T10 |
| *Acutuncus antarcticus* (tardigrade) | Ross Sea, Granite Harbour | Cesari et al. (2016) | T10 |
| *Acutuncus antarcticus* (tardigrade) | Ross Sea, Dry Valleys | Cesari et al. (2016) | T10 |
| *Desoria klovstadi* (springtail) | Ross Sea, Cape Jones | Stevens et al. (2007) in McGaughran et al. (2011) | T11, T12 |
| *Desoria klovstadi* (springtail) | Ross Sea, Crater Cirque | Stevens et al. (2007) in McGaughran et al. (2011) | T11, T12 |
| *Desoria klovstadi* (springtail) | Ross Sea, Daniell Peninsula | Stevens et al. (2007) in McGaughran et al. (2011) | T11, T12 |
| *Desoria klovstadi* (springtail) | Ross Sea, Hallett Peninsula–Football Saddle | Stevens et al. (2007) in McGaughran et al. (2011) | T11, T12 |
| *Desoria klovstadi* (springtail) | Ross Sea, Cape Hallett | Stevens et al. (2007) in McGaughran et al. (2011) | T11, T12 |
| *Gressittacantha terranova* (springtail) | Ross Sea, Terra Nova Bay 1 | Hawes et al. (2010) in McGaughran et al. (2011) | T13, T12 |
| *Gressittacantha terranova* (springtail) | Ross Sea, Terra Nova Bay 2 | Hawes et al. (2010) in McGaughran et al. (2011) | T13, T12 |
| *Gressittacantha terranova* (springtail) | Ross Sea, Terra Nova Bay 3 | Hawes et al. (2010) in McGaughran et al. (2011) | T13, T12 |
| *Gressittacantha terranova* (springtail) | Ross Sea, Terra Nova Bay 4 | Hawes et al. (2010) in McGaughran et al. (2011) | T13, T12 |
| *Gomphiocephalus hodgsoni* (springtail) | Ross Sea, Granite Harbour | McGaughran et al. (2009) in McGaughran et al. (2011) | T14, T12 |
| *Gomphiocephalus hodgsoni* (springtail) | Ross Sea, Dry Valleys | McGaughran et al. (2009) in McGaughran et al. (2011) | T14, T12 |
| *Gomphiocephalus hodgsoni* (springtail) | Ross Sea, southern Dry Valleys | McGaughran et al. (2009) in McGaughran et al. (2011) | T14, T12 |
| *Gomphiocephalus hodgsoni* (springtail) | Ross Sea, Ross Island | McGaughran et al. (2009) in McGaughran et al. (2011) | T14, T12 |
| *Cryptopygus antarcticus antarcticus* (springtail) | South Shetland Islands, King George Island | McGaughran et al. (2009) in McGaughran et al. (2011) | T14, T12 |
| *Cryptopygus antarcticus antarcticus* (springtail) | Central Antarctic Peninsula | McGaughran et al. (2009) in McGaughran et al. (2011) | T14, T12 |
| *Cryptopygus antarcticus antarcticus* (springtail) | Southern Antarctic Peninsula | McGaughran et al. (2009) in McGaughran et al. (2011) | T14, T12 |
| *Friesea grisea* (springtail) | Ross Sea, Cape Hallett | Torricelli et al (2010) in McGaughran et al. (2011) | T15, T12 |
| *Friesea grisea* (springtail) | Ross Sea, Crater Cirque | Torricelli et al (2010) in McGaughran et al. (2011) | T15, T12 |
| *Friesea grisea* (springtail) | South Shetland Islands | Torricelli et al (2010) in McGaughran et al. (2011) | T15, T12 |
| *Friesea grisea* (springtail) | Antarctic Peninsula, Adelaide Island | Torricelli et al (2010) in McGaughran et al. (2011) | T15, T12 |
| *Cryptopygus antarcticus travei* (springtail) | Marion Island | McGaughran et al. (2010) in McGaughran et al. (2011) | T16, T12 |
| *Tullbergia bisetosa* (springtail) | Marion Island | Myburgh et al. (2007) in McGaughran et al. (2011) | T17, T12 |

Supplementary Table 4. Locality and reference of existing studies investigating animal colonies as biological archives with a Quaternary focus.

| **Species** | **Locality** | **Reference** | **Reference ID** |
| --- | --- | --- | --- |
| *Pagodroma nivea* (snow petrel) | Dronning Maud Land, Petermann Range (1) | Berg et al. (2018) | A1 |
| *Pagodroma nivea* (snow petrel) | Dronning Maud Land, Petermann Range (2) | Berg et al. (2018) | A1 |
| *Pagodroma nivea* (snow petrel) | Dronning Maud Land, Petermann Range (3) | Berg et al. (2018) | A1 |
| *Pagodroma nivea* (snow petrel) | Dronning Maud Land, Untersee Oasis (1) | Berg et al. (2018) in McClymont et al. (2021) | A1, A29 |
| *Pagodroma nivea* (snow petrel) | Dronning Maud Land, Untersee Oasis (2) | Berg et al. (2018) | A1 |
| *Pagodroma nivea* (snow petrel) | Dronning Maud Land, Untersee Oasis (3) | Berg et al. (2018) | A1 |
| *Pagodroma nivea* (snow petrel) | Dronning Maud Land, Dalmann-Berge, E | Berg et al. (2018) | A1 |
| *Pagodroma nivea* (snow petrel) | Dronning Maud Land, Dallmann-Berge, SW | Berg et al. (2018) | A1 |
| *Pagodroma nivea* (snow petrel) | Dronning Maud Land, Scharffenbergbotnen/Heimefrontfjella (1) | Berg et al. (2018) | A1 |
| *Pagodroma nivea* (snow petrel) | Dronning Maud Land, Scharffenbergbotnen/Heimefrontfjella (2) | Berg et al. (2018) | A1 |
| *Pagodroma nivea* (snow petrel) | East Antarctica, Bunger Hills | Ainley et al. (2006) | A2 |
| *Mirounga leonina* (Southern elephant seal) | Ross Sea, Victoria Land Coast | de Bruyn et al. (2009) | A3 |
| *Mirounga leonina* (Southern elephant seal) | Macquarie Island | de Bruyn et al. (2009), Hall et al. (2006), de Bruyn et al. (2014) in Younger et al. (2015a) | A5, A6, A7 in A4 |
| *Leptonychotes weddellii* (Weddell seal) | Mawson Coast, Colbeck | Younger et al. (2016) | A8 |
| *Leptonychotes weddellii* (Weddell seal) | Prydz Bay, Amanda Bay | Younger et al. (2016) | A8 |
| *Leptonychotes weddellii* (Weddell seal) | Windmill Islands, Herring Islands | Younger et al. (2016) | A8 |
| *Leptonychotes weddellii* (Weddell seal) | Adélie Land, Pointe Geologie | Younger et al. (2016) | A8 |
| *Arctocephalus gazella* (Antarctic fur seals) | South Shetland Islands, Livingston Island | Cleary et al. (2021) | A9 |
| *Arctocephalus gazella* (Antarctic fur seals) | South Georgia, Bird Island | Cleary et al. (2021) | A9 |
| *Arctocephalus gazella* (Antarctic fur seals) | Bouvet Island | Cleary et al. (2021) | A9 |
| *Arctocephalus gazella* (Antarctic fur seals) | Kerguelen Islands | Cleary et al. (2021) | A9 |
| Seals | South Shetland Islands, King George Island, Fildes Peninsula | Huang et al. (2011b) | A10 |
| Penguins | South Shetland Islands, King George Island, Ardley peninsula | Sun et al. (2000) | A11 |
| *Aptenodytes patagonicus* (King penguin) | Crozet islands | Trucchi et al. (2014) | A12 |
| *Pygoscelis antarcticus* (Chinstrap penguin) | South Sandwich Islands, Zavodovski Island | Clucas et al. (2014) in Younger et al. (2015a) | A13 in A4 |
| *Pygoscelis antarcticus* (Chinstrap penguin) | South Orkney Islands, Signy Island | Clucas et al. (2014) in Younger et al. (2015a) | A13 in A4 |
| *Pygoscelis antarcticus* (Chinstrap penguin) | South Shetland Islands, King George Island | Clucas et al. (2014) in Younger et al. (2015a) | A13 in A4 |
| *Pygoscelis antarcticus* (Chinstrap penguin) | Antarctic Peninsula, Orne Harbour | Clucas et al. (2014) in Younger et al. (2015a) | A13 in A4 |
| *Pygoscelis papua* (Gentoo penguin) | South Orkney Islands, Signy Island | Clucas et al. (2014) in Younger et al. (2015a) | A13 in A4 |
| *Pygoscelis papua* (Gentoo penguin) | South Sandwich Islands, Saunders Island | Clucas et al. (2014) in Younger et al. (2015a) | A13 in A4 |
| *Pygoscelis papua* (Gentoo penguin) | Falkland Islands, Volunteer Point | Clucas et al. (2014) in Younger et al. (2015a) | A13 in A4 |
| *Pygoscelis papua* (Gentoo penguin) | South Georgia, Bird Island | Clucas et al. (2014) in Younger et al. (2015a) | A13 in A4 |
| *Pygoscelis papua* (Gentoo penguin) | South Shetland Islands, King George Island | Clucas et al. (2014) in Younger et al. (2015a) | A13 in A4 |
| *Pygoscelis papua* (Gentoo penguin) | Antarctic Peninsula, Port Lockroy | Clucas et al. (2014) in Younger et al. (2015a) | A13 in A4 |
| *Pygoscelis papua* (Gentoo penguin) | Elephant Island | Peña et al. (2014) in Younger et al. (2015a) | A14 in A4 |
| *Pygoscelis papua* (Gentoo penguin) | South Shetland Islands, King Geroge Island, Admiralty Bay | Peña et al. (2014) in Younger et al. (2015a) | A14 in A4 |
| *Pygoscelis papua* (Gentoo penguin) | South Shetland Islands, King Geroge Island, Ardley Island | Peña et al. (2014) in Younger et al. (2015a) | A14 in A4 |
| *Pygoscelis papua* (Gentoo penguin) | Antarctic Peninsula, O’Higgins Base | Peña et al. (2014) in Younger et al. (2015a) | A14 in A4 |
| *Pygoscelis papua* (Gentoo penguin) | Antarctic Peninsula, Gabriel Gonzalez Videla Base | Peña et al. (2014) in Younger et al. (2015a) | A14 in A4 |
| *Pygoscelis papua* (Gentoo penguin) | South Shetland Islands, King George Island, Potter Peninsula | Del Valle et al. (2002) in Younger et al. (2015a) | A15 in A4 |
| *Pygoscelis papua* (Gentoo penguin) | South Shetland Islands, King George Island, Potter Peninsula | Del Valle et al. (2002) in Younger et al.(2015a) | A15 in A4 |
| *Pygoscelis adeliae* (Adelie penguin) | South Shetland Islands, King George Island | Clucas et al. (2014) in Younger et al. (2015a) | A13 in A4 |
| *Pygoscelis adeliae* (Adelie penguin) | South Orkney Islands, Signy Island | Clucas et al. (2014) in Younger et al. (2015a) | A13 in A4 |
| *Pygoscelis adeliae* (Adelie penguin) | South Sandwich Islands, Saunders Island | Clucas et al. (2014) in Younger et al. (2015a) | A13 in A4 |
| *Pygoscelis adeliae* (Adelie penguin) | Antarctic Peninsula, Lagotellerie | Clucas et al. (2014) in Younger et al. (2015a) | A13 in A4 |
| *Pygoscelis adeliae* (Adelie penguin) | Ross Sea, Inexpressible Island | Emslie et al. (2007) | A17 |
| *Pygoscelis adeliae* (Adelie penguin) | Ross Sea, Edmonson Point | Emslie et al. (2007) | A17 |
| *Pygoscelis adeliae* (Adelie penguin) | Ross Sea, Cape Hallett | Emslie et al. (2007) | A17 |
| *Pygoscelis adeliae* (Adelie penguin) | Ross Sea, Beaufort Island | Emslie et al. (2007) | A17 |
| *Pygoscelis adeliae* (Adelie penguin) | Ross Sea, Cape Adare | Emslie et al. (2007) | A17 |
| *Pygoscelis adeliae* (Adelie penguin) | Ross Sea, Adelie Cove | Emslie et al. (2007) | A17 |
| *Pygoscelis adeliae* (Adelie penguin) | Ross Sea, Campo Icarus | Emslie et al. (2007) | A17 |
| *Pygoscelis adeliae* (Adelie penguin) | Ross Sea, Clear Lake | Emslie et al. (2007) | A17 |
| *Pygoscelis adeliae* (Adelie penguin) | Ross Sea, Prior Island | Emslie et al. (2007) | A17 |
| *Pygoscelis adeliae* (Adelie penguin) | East Antarctica, Windmill Islands, Shirley Island | Emslie & Patterson (2007) in Younger et al. (2015a) | A18 in A4 |
| *Pygoscelis adeliae* (Adelie penguin) | East Antarctica, Wilkes | Emslie & Patterson (2007) in Younger et al. (2015a) | A18 in A4 |
| *Pygoscelis adeliae* (Adelie penguin) | Ross Sea, Edmonson Point | Emslie & Patterson (2007) in Younger et al. (2015a) | A18 in A4 |
| *Pygoscelis adeliae* (Adelie penguin) | Ross Sea, Cape Hallett | Emslie & Patterson (2007) in Younger et al. (2015a) | A18 in A4 |
| *Pygoscelis adeliae* (Adelie penguin) | Ross Sea, Inexpressible Island | Emslie & Patterson (2007) in Younger et al. (2015a) | A18 in A4 |
| *Pygoscelis adeliae* (Adelie penguin) | South Shetland Islands, King George Island | Emslie & Patterson (2007) in Younger et al. (2015a) | A18 in A4 |
| *Pygoscelis adeliae* (Adelie penguin) | Ross Sea, Cape Crozier | Emslie & Patterson (2007) in Younger et al. (2015a) | A18 in A4 |
| *Pygoscelis adeliae* (Adelie penguin) | Antarctic Peninsula, Palmer Station | Emslie & Patterson (2007) in Younger et al. (2015a) | A18 in A4 |
| *Pygoscelis adeliae* (Adelie penguin) | Antarctic Peninsula, Cape Evans, Scott’s Hut | Emslie & Patterson (2007) in Younger et al. (2015a) | A18 in A4 |
| *Pygoscelis adeliae* (Adelie penguin) | Ross Sea, Cape Crozier | Ritchie et al. (2004), Lambert et al. (2002) in  Younger et al (2015a) | A19, A20 in A4 |
| *Pygoscelis adeliae* (Adelie penguin) | Ross Sea, Cape Bird | Ritchie et al. (2004), Lambert et al. (2002) in  Younger et al (2015a) | A19, A20 in A4 |
| *Pygoscelis adeliae* (Adelie penguin) | Ross Sea, Beaufort Island | Ritchie et al. (2004), Lambert et al. (2002) in Younger et al (2015a) | A19, A20 in A4 |
| *Pygoscelis adeliae* (Adelie penguin) | Ross Sea, Marble Point | Ritchie et al. (2004), Lambert et al. (2002) in Younger et al (2015a) | A19, A20 in A4 |
| *Pygoscelis adeliae* (Adelie penguin) | Ross Sea, Cape Roberts | Ritchie et al. (2004), Lambert et al. (2002) in Younger et al (2015a) | A19, A20 in A4 |
| *Pygoscelis adeliae* (Adelie penguin) | Ross Sea, Cape Ross | Ritchie et al. (2004), Lambert et al. (2002) in Younger et al (2015a) | A19, A20 in A4 |
| *Pygoscelis adeliae* (Adelie penguin) | Ross Sea, Depot Island | Ritchie et al. (2004), Lambert et al. (2002) in Younger et al (2015a) | A19, A20 in A4 |
| *Pygoscelis adeliae* (Adelie penguin) | Ross Sea, Cape Day | Ritchie et al. (2004), Lambert et al. (2002) in Younger et al (2015a) | A19, A20 in A4 |
| *Pygoscelis adeliae* (Adelie penguin) | Ross Sea, Cape Hickey | Ritchie et al. (2004), Lambert et al. (2002) in Younger et al (2015a) | A19, A20 in A4 |
| *Pygoscelis adeliae* (Adelie penguin) | Ross Sea, Inexpressible Island | Ritchie et al. (2004), Lambert et al. (2002) in Younger et al (2015a) | A19, A20 in A4 |
| *Pygoscelis adeliae* (Adelie penguin) | Ross Sea, Adelie Cove | Ritchie et al. (2004), Lambert et al. (2002) in Younger et al (2015a) | A19, A20 in A4 |
| *Pygoscelis adeliae* (Adelie penguin) | Ross Sea, Northern Foothills | Ritchie et al. (2004), Lambert et al. (2002) in Younger et al (2015a) | A19, A20 in A4 |
| *Pygoscelis adeliae* (Adelie penguin) | Ross Sea, Gondwana Station | Ritchie et al. (2004), Lambert et al. (2002) in Younger et al (2015a) | A19, A20 in A4 |
| *Pygoscelis adeliae* (Adelie penguin) | Ross Sea, Edmonson Point | Ritchie et al. (2004), Lambert et al. (2002) in Younger et al (2015a) | A19, A20 in A4 |
| *Pygoscelis adeliae* (Adelie penguin) | Ross Sea, Cape Hallett | Ritchie et al. (2004), Lambert et al. (2002) in Younger et al (2015a) | A19, A20 in A4 |
| *Pygoscelis adeliae* (Adelie penguin) | East Antarctica, Mac. Robertson Land, Welch    Island | Ritchie et al. (2004), Lambert et al. (2002) in Younger et al (2015a) | A19, A20 in A4 |
| *Pygoscelis adeliae* (Adelie penguin) | East Antarctica, Prydz Bay, Gardner Island | Ritchie et al. (2004), Lambert et al. (2002) in Younger et al (2015a) | A19, A20 in A4 |
| *Pygoscelis adeliae* (Adelie penguin) | Antarctic Peninsula, Torgersen Island | Ritchie et al. (2004), Lambert et al. (2002) in Younger et al (2015a) | A19, A20 in A4 |
| *Pygoscelis adeliae* (Adelie penguin) | Ross Sea, Cape Royds | Ritchie et al. (2004), Lambert et al. (2002) in Younger et al (2015a) | A19, A20 in A4 |
| *Pygoscelis adeliae* (Adelie penguin) | Ross Sea, Franklin Island | Ritchie et al. (2004), Lambert et al. (2002) in Younger et al (2015a) | A19, A20 in A4 |
| *Pygoscelis adeliae* (Adelie penguin) | Ross Sea, Cape Wheatstone | Ritchie et al. (2004), Lambert et al. (2002) in Younger et al (2015a) | A19, A20 in A4 |
| *Pygoscelis adeliae* (Adelie penguin) | Ross Sea, Cape Adare | Ritchie et al. (2004), Lambert et al. (2002) in Younger et al (2015a) | A19, A20 in A4 |
| *Pygoscelis adeliae* (Adelie penguin) | Adélie Land, Port Martin | Ritchie et al. (2004), Lambert et al. (2002) in Younger et al (2015a) | A19, A20 in A4 |
| *Pygoscelis adeliae* (Adelie penguin) | Balleny Islands | Ritchie et al. (2004), Lambert et al. (2002) in Younger et al (2015a) | A19, A20 in A4 |
| *Pygoscelis adeliae* (Adelie penguin) | East Antarctica, Wilkes Land, Blakeney Point | Younger et al. (2015b) | A21 |
| *Pygoscelis adeliae* (Adelie penguin) | East Antarctica, Windmill Islands, Holl Island | Younger et al. (2015b) | A21 |
| *Pygoscelis adeliae* (Adelie penguin) | East Antarctica, Mac. Robertson Land, Macey Island | Younger et al. (2015b) | A21 |
| *Pygoscelis adeliae* (Adelie penguin) | East Antarctica, Mac. Robertson Land, Welch Island | Younger et al. (2015b) | A21 |
| *Pygoscelis adeliae* (Adelie penguin) | East Antarctica, Mac. Robertson Land, Bechervaise Island | Younger et al. (2015b) | A21 |
| *Pygoscelis adeliae* (Adelie penguin) | East Antarctica, Adélie Land, Pétrels Island | Younger et al. (2015b) | A21 |
| *Pygoscelis adeliae* (Adelie penguin) | East Antarctica, Prydz Bay, Zolotov Island | Huang et al. (2011a) | A22 |
| *Pygoscelis adeliae* (Adelie penguin) | Ross Sea, Cape Irizar | Emslie, 2021 | A23 |
| *Pygoscelis adeliae* (Adelie penguin) | Ross Sea, Inexpressible Island | Xu et al. (2020) | A24 |
| *Aptenodytes forsteri* (Emperor penguin) | Antarctic Peninsula, Marguerite Bay, Emperor Island | Li et al. (2014) | A25 |
| *Pygoscelis adeliae* (Adelie penguin) | Ross Sea, Inexpressible Island | Li et al. (2014) | A25 |
| *Aptenodytes forsteri* (Emperor penguin) | Ross Sea, Cape Crozier | Younger et al. (2015c) | A27 |
| *Aptenodytes forsteri* (Emperor penguin) | Ross Sea, Cape Washington | Younger et al. (2015c) | A27 |
| *Aptenodytes forsteri* (Emperor penguin) | Weddell Sea, Halley Bay | Younger et al. (2015c) | A27 |
| *Aptenodytes forsteri* (Emperor penguin) | Weddell Sea, Gould Bay | Younger et al. (2015c) | A27 |
| *Aptenodytes forsteri* (Emperor penguin) | East Antarctica, Kemp Land Coast, Fold Island | Younger et al. (2015c) | A27 |
| *Aptenodytes forsteri* (Emperor penguin) | East Antarctica, Kemp Land Coast, Auster | Younger et al. (2015c) | A27 |
| *Aptenodytes forsteri* (Emperor penguin) | East Antarctica, Prydz Bay, Amanda Bay | Younger et al. (2015c) | A27 |
| *Aptenodytes forsteri* (Emperor penguin) | East Antarctica, Prydz Bay, Club Lake | Younger et al. (2015c) | A27 |
| *Aptenodytes forsteri* (Emperor penguin) | Adélie Land, Pointe Geologie | Younger et al. (2015c) | A27 |
| *Aptenodytes forsteri* (Emperor penguin) | East Antarctica, Prydz Bay, Amanda Bay | Huang et al. (2016) | A26 |
| *Eudyptes filholi* (Eastern rockhopper penguin) | Macquarie Island | Frugone et al. (2018) | A16 |
| *Eudyptes filholi* (Eastern rockhopper penguin) | Kerguelen Island | Frugone et al. (2018) | A16 |
| *Eudyptes filholi* (Eastern rockhopper penguin) | Crozet Island | Frugone et al. (2018) | A16 |
| *Eudyptes filholi* (Eastern rockhopper penguin) | Marion Island | Frugone et al. (2018) | A16 |
| *Eudyptes chrysolophus* (Macaroni penguin) | Kerguelen Island | Frugone et al. (2018) | A16 |
| *Eudyptes chrysolophus* (Macaroni penguin) | Crozet Island | Frugone et al. (2018) | A16 |
| *Eudyptes chrysolophus* (Macaroni penguin) | Marion Island | Frugone et al. (2018) | A16 |
| *Eudyptes chrysolophus* (Macaroni penguin) | Bouvet Island | Frugone et al. (2018) | A16 |
| *Eudyptes chrysolophus* (Macaroni penguin) | Bird Island | Frugone et al. (2018) | A16 |
| *Eudyptes chrysolophus* (Macaroni penguin) | Elephant Island | Frugone et al. (2018) | A16 |
| *Eudyptes schlegeli* (Royal penguin) | Macquarie Island | Frugone et al. (2018) | A16 |

Supplementary Table 5. Locality and reference of existing studies investigating live mosses or peats as biological archives with a Quaternary focus.

| **Locality** | **Reference** | **Reference ID** |
| --- | --- | --- |
| East Antarctica, Bailey Peninsula, ASPA135 (1) | Robinson et al. (2018) | MP1 |
| East Antarctica, Bailey Peninsula, ASPA135 (2) | Clarke et al. 2012, Robinson et al. (2018) | MP1, MP2 |
| East Antarctica, Bailey Peninsula, Casey Station (Red Shed) (1) | Clarke et al. 2012, Robinson et al. (2018) | MP1, MP2 |
| East Antarctica, Bailey Peninsula, Casey Station (Red Shed) (2) | Robinson et al. (2018) | MP1 |
| East Antarctica, Bailey Peninsula, Burrows Wallow (1) | Robinson et al. (2018) | MP1 |
| East Antarctica, Bailey Peninsula, Casey Station (Red Shed) (3) | Robinson et al. (2018) | MP1 |
| East Antarctica, Bailey Peninsula, Burrows Wallow (2) | Robinson et al. (2018) | MP1 |
| East Antarctica, Bailey Peninsula, Burrows Wallow (3) | Robinson et al. (2018) | MP1 |
| East Antarctica, Bailey Peninsula, Casey Station, Red Shed (4) | Robinson et al. (2018) | MP1 |
| East Antarctica, Clark Peninsula, C2 (1) | Robinson et al. (2018) | MP1 |
| East Antarctica, Clark Peninsula, C2 (2) | Clarke et al. 2012, Robinson et al. (2018) | MP1, MP2 |
| East Antarctica, Clark Peninsula, C2 (3) | Robinson et al. (2018) | MP1 |
| East Antarctica, Clark Peninsula, C2 (4) | Robinson et al. (2018) | MP1 |
| East Antarctica, Clark Peninsula, C1 (5) | Robinson et al. (2018) | MP1 |
| East Antarctica, Clark Peninsula, C1 (6) | Robinson et al. (2018) | MP1 |
| East Antarctica, Clark Peninsula, Clark Ridge | Clarke et al. 2012, Robinson et al. (2018) | MP1, MP2 |
| East Antarctica, Windmill Islands, Robinson Ridge (1) | Robinson et al. (2018) | MP1 |
| East Antarctica, Windmill Islands, Robinson Ridge (2) | Robinson et al. (2018) | MP1 |
| East Antarctica, Vestfold Hills, Grimmia Gorge (1) | Clarke et al. 2012 | MP2 |
| East Antarctica, Vestfold Hills, Grimmia Gorge (2) | Clarke et al. 2012 | MP2 |
| Elephant Island | Björck et al. (1991b) | MP3 |
| Elephant Island | Charman et al. (2018) | MP4 |
| Antarctic Peninsula, Green Island | Charman et al. (2018) | MP4 |
| Antarctic Peninsula, Green Island | Amesbury et al. (2017) | MP5 |
| Antarctic Peninsula, Barrientos Island | Charman et al. (2018) | MP4 |
| Antarctic Peninsula, Norsel Point | Charman et al. (2018) | MP4 |
| Antarctic Peninsula, Cape Rasmussen | Loisel et al. (2017) | MP6 |
| Antarctic Peninsula, around Anvers Island and Galindez Island | Yu et al. (2016) | MP7 |
| Antarctic Peninsula, around Lazarev Bay,  Alexander Island, west of Antarctic Peninsula | Royles et al. (2013) | MP8 |
| South Orkney Is;ands, Signy Island | Royles et al. (2012) | MP9 |
| Antarctic Peninsula, Litchfield Island | Stelling et al. (2018) | MP10 |
| Antarctic Peninsula, Cape Rasmussen | Loisel et al. (2017) | MP11 |
| Macquarie Island, Green Gorge | Bergstrom et al. (2002) | MP12 |
| Campbell Island | McGlone et al. (2007), McGlone et al. (2010) | MP13, MP14 |
| Falkland Islands | Turney et al. (2016) | MP15 |
| South Georgia | Van der Putten et al. (2009a), Van der Putten et al. (2009b) | MP16, MP17 |
| Crozet Island | Van der Putten et al. (2008) | MP18 |
| Marion Island | Yeloff et al. (2007) | MP19 |
| South Georgia | Barrow (1978) | MP20 |
| Marion Island | Miller & Heatwole (2003) | MP21 |
| Antarctic Peninsula, Alexander Island | Convey et al. (2011) | MP22 |
| Auckland Island | McGlone et al. (2000) | MP23 |
| Auckland Islands, Enderby Island | Fleming et al. (1976) in McGlone (2002) | MP24, MP25 |
| Campbell Island | Moar (1973) in McGlone (2002), McGlone & Moar (1997) | MP26, MP25, MP27 |
| Macquarie Island | Selkirk et al. (1983), Selkirk et al.(1988) | MP28, MP29 |
| Kerguelen Island | Van der Putten et al. (2015) | MP30 |

Supplementary Table 6. Locality and reference of existing studies investigating lake sediment as biological archives with a Quaternary focus.

| **Locality** | **Reference** | **Reference ID** |
| --- | --- | --- |
| Auckland Island | Browne et al. (2017) | L1 |
| South Shetland Islands, King George Island, Ardley Island, Ardley Lake | Roberts et al. (2017) | L2 |
| South Georgia, Annenkov Island, Fan Lake | Foster et al. (2016) | L3 |
| South Georgia, Annenkov Island, Fan Lake | Strother et al. (2015) | L4 |
| Macquarie Island, Emerald Lake | Saunders et al. (2018) | L5 |
| Windmill Islands, Beall island, Lacustrine site | Gasparon et al. (2007) | L6 |
| Windmill Islands, Browning peninsula, Lacustrine site | Gasparon et al. (2007) | L6 |
| Windmill Islands, Peterson island, Lacustrine site | Gasparon et al. (2007) | L6 |
| Windmill Islands, Peterson island, Lacustrine site | Gasparon et al. (2007) | L6 |
| Windmill Islands, Robertson channel, Marine site | Gasparon et al. (2007) | L6 |
| Windmill Islands, Stevenson cove, Marine site | Gasparon et al. (2007) | L6 |
| Vestfold Hills, Ace Lake | Roberts & McMinn 1999, Roberts et al. (2001b) | L7, L8 |
| Vestfold Hills | Bird et al. (1991) | L9 |
| Antarctic Peninsula, Alexander Island, Hodgson Lake | Hodgson et al. (2009) | L10 |
| East Antarctica, Amery Oasis, Lake Terrasovoje | Wagner et al. (2004) | L11 |
| East Antarctica, Schirmacher Oasis | Govil et al. (2016) | L12 |
| South Shetland Islands, Livingston Island | Björck et al. (1991a) | L13 |
| Antarctic Peninsula, James Ross Island | Björck et al. (1996) | L14 |
| Antarctic Peninsula, Vega Island, Lake Esmeralda | Píšková et al. (2019) | L15 |
| East Antarctica, Rauer Islands, Flag Island depression (isolation basin) | Berg et al. (2010), Hodgson et al. (2016) | L16, L76 |
| Larsemann Hills, Pup Lagoon (isolation basin) | Verleyen et al. (2004), Verleyen et al. (2005) | L17, L18 |
| Larsemann Hills, Heard Lake (isolation basin) | Verleyen et al. (2005) in Hodgson et al. 2016 | L18, L76 |
| Larsemann Hills, Kirisjes Pond (isolation basin) | Verleyen et al. (2005) in Hodgson et al. 2016 | L18, L76 |
| Vestfold Hills, Organic Lake (isolation basin) | Bird et al. (1991) in Hodgson et al. 2016 | L9, L76 |
| Vestfold Hills, Highway Lake (isolation basin) | Bird et al. (1991) | L9 |
| Vestfold Hills, Watts Lake (isolation basin) | Zwartz et al. (1998) in Hodgson et al. 2016 | L19, L76 |
| Vestfold Hills, Lake Duzhby (isolation basin) | Zwartz et al. (1998) in Hodgson et al. 2016 | L19, L76 |
| Vestfold Hills, Anderson Lake (isolation basin) | Zwartz et al. (1998) in Hodgson et al. 2016 | L19, L76 |
| Vestfold Hills, Ace Lake (isolation basin) | Zwartz et al. (1998), Coolen et al. (2004a) in Hodgson et al. 2016 | L19, L20, L76 |
| Marion Island, Lake on La Grange Kop | Perren et al. (2020) | L21 |
| South Shetland Islands | Björck et al. (2013) | L22 |
| South Orkney Islands, Signy Island | Jones et al. (2000) | L23 |
| East Antarctica, Bunger Hills | Berg et al. (2020) | L24 |
| East Antarctica, Bunger Hills, 17 m lake | Kulbe (1997) | L25 |
| East Antarctica, Bunger Hills, 8 m lake | Kulbe (1997) | L25 |
| East Antarctica, Bunger Hills, Algae Lake | Kulbe (1997) | L25 |
| East Antarctica, Bunger Hills, Algae Lake | Melles et al. (1994); Verkulich et al. (2002) | L26, L27 |
| East Antarctica, Bunger Hills, Izvilistaja Inlet | Kulbe (1997) | L25 |
| East Antarctica, Bunger Hills, Paz Cove | Kulbe (1997) | L25 |
| East Antarctica, Bunger Hills, Paz Cove | Kulbe et al. (2001) | L28 |
| East Antarctica, Bunger Hills, Pol'anskogo Lake | Melles et al. (1995) | L28 |
| East Antarctica, Bunger Hills, Jaw Lake | Roberts et al. (2000) | L30 |
| South Georgia | Berg et al. (2019) | L31 |
| East Antarctica, Larsemann Hills | Hodgson et al. (2005) | L32 |
| Kerguelen Islands | Ficetola et al. (2018) | L33 |
| East Antarctica, Larsemann Hills, Lake Reid | Cromer et al. (2006) | L34 |
| East Antarctica, Larsemann Hills, Lake Reid | Hodgson et al. (2005) | L35 |
| East Antarctica, Larsemann Hills, Progress Lake | Hodgson et al. (2006) | L36 |
| East Antarctica, Vestfold Hills, Ace Lake | Cromer et al. (2005) | L37 |
| Antarctic Peninsula, Hope Bay, Lake Boeckella | Zale & Karlen (1989) | L38 |
| Antarctic Peninsula, James Ross Island, Hidden Lake | Zale & Karlen (1989) | L38 |
| Macquarie Island, Palaeolake Skua | Keenan (1995) | L39 |
| Antarctic Peninsula, Hope Bay, Lake Boeckella | Zale (1993), Björck et al. (1991c) | L40, L41 |
| South Shetland Islands, King George Island | Oliva et al. (2019) | L42 |
| East Antarctica, Vestfold Hills, Abraxas Lake | Gibson et al. (2009) | L43 |
| East Antarctica, Larsemann Hills, Lake Reid | Gibson & Bayly (2007) | L44 |
| East Antarctica, Amery Oasis, Beaver Lake | Wagner et al. (2007) | L45 |
| South Orkney Islands, Signy Island | Hodgson & Convey (2005) | L46 |
| South Shetland Islands, King George Island, Ardley Island | Liu et al. (2005) | L47 |
| South Orkney Islands, Signy Island, Sombre Lake | Noon et al. (2003) | L48 |
| East Antarctica, Lützow–Holm Bay, Lake Abi | Rudd et al. (2016) | L49 |
| South Orkney Islands, Signy Island | Hodgson & Convey (2005) | L46 |
| Ross Sea, Ross Sea Island | Chen et al. (2013) | L51 |
| Ross Sea, Beaufort Island | Chen et al. (2013) | L51 |
| Larsemann Hills, Progress Lake | Cromer et al. (2008) | L52 |
| East Antarctica, Vestfold Hills, Ace Lake | Coolen et al. (2004b) | L53 |
| Antarctic Peninsula, Livingston Island, Limnopolar Lake | Gibson et al. (2007) | L54 |
| Larsemann Hills, Lake Reid | Gibson et al. (2007) | L54 |
| Vestfold Hills, Waterfall Lake | Gibson et al. (2007) | L54 |
| Amery Oasis, Lake Terrasovoje | Gibson et al. (2007) | L54 |
| East Antarctica, Vestfold Hills, Ace Lake | Coolen et al. (2004a) | L20 |
| East Antarctica, Vestfold Hills, Ace Lake | Roberts et al. (1999) | L55 |
| South Shetland Islands, Livingston Island, Byers Peninsula | Aymerich et al. (2016) | L56 |
| South Shetland Islands, King George Island, Fildes Peninsula | Vieira et al. (2015) | L57 |
| Ross Sea, McMurdo Dry Valleys | Hendy (2000) | L58 |
| East Antarctica, Larsemann Hills, Kirisjes Pond | Squier et al. (2002) | L59 |
| South Shetland Islands, Livingston Island, Byers Peninsula, Limnopolar Lake | Toro et al. (2013) | L60 |
| Antarctic Peninsula, Lallemand Fjord | Shevenell et al. (1996) in Bentley et al. (2009) | L61, L62 |
| Ross Sea, southern Victoria Land, McMurdo Dry Valleys, Lake Hoare | Squyres et al. (1991) in Ingólfsson et al. (1998) | L64, L63 |
| East Antarctica, Bunger Hills, Figurnoe Lake (1) | Melles et al. (1994) in Ingólfsson et al. (1998) | L26, L63 |
| East Antarctica, Bunger Hills, Figurnoe Lake (2) | Melles et al. (1994) in Ingólfsson et al. (1998) | L26, L63 |
| East Antarctica, central Holl Island, Holl Lake | Goodwin (1993) in Ingólfsson et al. (1998) | L66, L63 |
| East Antarctica, northern Holl Island, Holl Pond A | Goodwin (1993) in Ingólfsson et al. (1998) | L66, L63 |
| East Antarctica, northwestern Bailey Peninsula, Bailey Pond | Goodwin (1993) in Ingólfsson et al. (1998) | L66, L63 |
| East Antarctica, western Mitchell Peninsula, Mitchell Pond B | Goodwin (1993) in Ingólfsson et al. (1998) | L66, L63 |
| East Antarctica, Shirey Island, Shirley Pond | Goodwin (1993) in Ingólfsson et al. (1998) | L66, L63 |
| Antarctic Peninsula, Porquoi-Pas & Horseshoe Is | Wasell & Håkansson (1992) in Verleyen et al. (2011) | L67, L65 |
| Antarctic Peninsula, Alexander Island, Ablation Point | Heywood (1977) in Verleyen et al. (2011) | L68, L65 |
| East Antarctica, Schirmacher Oasis | Schwab (1998) in Verleyen et al. (2011) | L69, L65 |
| East Antarctica, Untersee Oasis | Schwab (1998) in Verleyen et al. (2011) | L69, L65 |
| Mt Riiser-Larsen | Zwartz et al. (1998b) in Verleyen et al. (2011) | L70, L65 |
| East Antarctica, Vestfold Hills | Verleyen et al. (2003) in Verleyen et al. (2011) | L71, L65 |
| East Antarctica, Larsemann Hills | Verleyen et al. (2003) in Verleyen et al. (2011) | L71, L65 |
| East Antarctica, Prydz Bay, Rauer Islands | Verleyen et al. (2003) in Verleyen et al. (2011) | L71, L65 |
| East Antarctica, Prydz Bay, Bølingen Islands | Verleyen et al. (2003) in Verleyen et al. (2011) | L71, L65 |
| East Antarctica, Windmill Islands | Verleyen et al. (2003) in Verleyen et al. (2011) | L71, L65 |
| East Antarctica, Windmill Islands, Bell Island | Roberts et al. (2001a) in Verleyen et al. (2011) | L72, L65 |
| East Antarctica, Windmill Islands, Holl Island | Roberts et al. (2001a) in Verleyen et al. (2011) | L72, L65 |
| East Antarctica, Windmill Islands, Warrington Island | Roberts et al. (2001a) in Verleyen et al. (2011) | L72, L65 |
| East Antarctica, Windmill Islands, Browning Peninsula (1) | Roberts et al. (2001a) in Verleyen et al. (2011) | L72, L65 |
| East Antarctica, Windmill Islands, Browning Peninsula (2) | Roberts et al. (2001a) in Verleyen et al. (2011) | L72, L65 |
| East Antarctica, Windmill Islands, Browning Peninsula (3) | Roberts et al. (2001a) in Verleyen et al. (2011) | L72, L65 |
| East Antarctica, Windmill Islands, Browning Peninsula (4) | Roberts et al. (2001a) in Verleyen et al. (2011) | L72, L65 |
| East Antarctica, Windmill Islands, Browning Peninsula (5) | Roberts et al. (2001a) in Verleyen et al. (2011) | L72, L65 |
| East Antarctica, Windmill Islands, Browning Peninsula (6) | Roberts et al. (2001a) in Verleyen et al. (2011) | L72, L65 |
| East Antarctica, Windmill Islands, Peterson Island (1) | Roberts et al. (2001a) in Verleyen et al. (2011) | L72, L65 |
| East Antarctica, Windmill Islands, Peterson Island (2) | Roberts et al. (2001a) in Verleyen et al. (2011) | L72, L65 |
| East Antarctica, Windmill Islands, Peterson Island (3) | Roberts et al. (2001a) in Verleyen et al. (2011) | L72, L65 |
| East Antarctica, Windmill Islands, Peterson Island (4) | Roberts et al. (2001a) in Verleyen et al. (2011) | L72, L65 |
| East Antarctica, Windmill Islands, Browning Peninsula (7) | Roberts et al. (2001a) in Verleyen et al. (2011) | L72, L65 |
| Antarctic Peninsula, Hope Bay, Lake Boeckella | Gibson & Zale (2006), Gibson et al. (2007) | L73, L54 |
| Vestfold Hills, Gardner Island | Huang et al. (2009a), Huang et al. (2009b) in Hodgson et al. 2016 | L74, L75, L76 |
| Vestfold Hills, Zolotov Island | Huang et al. (2011a) in Hodgson et al. (2016) | L50, L76 |

**References (for supplementary Table 1-6 only)**

Ainley, D. G., Hobson, K. A., Crosta, X., Rau, G. H., Wassenaar, L. I., & Augustinus, P. C. (2006). Holocene variation in the Antarctic coastal food web: linking δD and δ13C in snow petrel diet and marine sediments. *Marine Ecology Progress Series*, **306**, 31-40. https://doi.org/10.3354/meps306031.

Allen, C. S., Oakes-Fretwell, L., Anderson, J. B., & Hodgson, D. A. (2010). A record of Holocene glacial and oceanographic variability in Neny Fjord, Antarctic Peninsula. *The Holocene*, **20**(4), 551-564.

Allegrucci, G., Carchini, G., Convey, P., & Sbordoni, V. (2012). Evolutionary geographic relationships among orthocladine chironomid midges from maritime Antarctic and sub-Antarctic islands. *Biological Journal of the Linnean Society*, **106**(2), 258–274. https://doi.org/10.1111/j.1095-8312.2012.01864.x.

Alley, K., Patacca, K., Pike, J., Dunbar, R., & Leventer, A. (2018). Iceberg Alley, East Antarctic Margin: Continuously laminated diatomaceous sediments from the late Holocene. *Marine Micropaleontology*, **140**, 56–68. https://doi.org/10.1016/j.marmicro.2017.12.002.

Amesbury, M. J., Roland, T. P., Royles, J., Hodgson, D. A., Convey, P., Griffiths, H., & Charman, D. J. (2017).

Widespread Biological Response to Rapid Warming on the Antarctic Peninsula. *Current Biology*, **27**(11), 1616-1622.e2. http://dx.doi.org/10.1016/j.cub.2017.04.034.

Anderson, J. B., Warny, S., Askin, R. A., Wellner, J. S., Bohaty, S. M., Kirshner, A. E., Livsey, D. N., Simms, A. R., Smith, T. R., Ehrmann, W., Lawver, L. A., Barbeau, D., Wise, S. W., Kulhanek, D. K., Weaver, F. M., & Majewski, W. (2011). Progressive Cenozoic cooling and the demise of Antarctica's last refugium. *Proceedings of the National Academy of Sciences of the United States of America*, **108**(28), 11356–11360. https://doi.org/10.1073/pnas.1014885108.

Ashley, K. E., Crosta, X., Etourneau, J., Campagne, P, Gilchrist, H., Ibraheem, U., Greene, S. E., Schmidt, S., Eley, Y., Massé, G., & Bendle, J. (2021a). Exploring the use of compound-specific carbon isotopes as a palaeoproductivity proxy off the coast of Adélie Land, East Antarctica. *Biogeosciences*, **18**(9), 5555–5571. https://doi.org/10.5194/bg-18-5555-2021.

Ashley, K. E., McKay, R., Etourneau, J., Jimenez-Espejo, F. J., Condron, A., Albot, A., Crosta, X., Riesselman, C., Seki, O., Massé, G., Golledge, N. R., Gasson, E., Lowry, D. P., Barrand, N. E., Johnson, K., Bertler, N., Escutia, C., Dunbar, R., & Bendle, J. A. (2021b). Mid-Holocene Antarctic sea-ice increase driven by marine ice sheet retreat. *Climate of the Past*, **17**, 1–19. https://doi.org/10.5194/cp-17-1-2021.

Aymerich, I. F., Oliva, M., Giralt, S., & Martín-Herrero, J. (2016). Detection of Tephra Layers in Antarctic Sediment Cores with Hyperspectral Imaging. *PLoS ONE*, **11**(1), e0146578. https://doi.org/10.1371/journal.pone.0146578.

Baird, H. P., Shin, S., Oberprieler, R. G., Hullé, M., Vernon, P., Moon, K. L., Adams, R. H., McKenna, D. D., & Chown, S. L. (2021). Fifty million years of beetle evolution along the Antarctic Polar Front. *Proceedings of the National Academy of Sciences of the United States of America*, **118**(24), e2017384118. https://doi.org/10.1073/pnas.2017384118.

Barbara, L., Crosta, X., Leventer, A., Schmidt, S., Etourneau, J., Domack, E., & Massé, G. (2016). Environmental responses of the Northeast Antarctic Peninsula to the Holocene climate variability. *Paleoceanography and Paleoclimatology*, **31**(1), 131–147. https://doi.org/10.1002/2015PA002785.

Barrow, C.J. (1978). Postglacial pollen diagrams from South Georgia (sub-Antarctic) and West Falkland island (South Atlantic). *Journal of Biogeography*, **5**, 251-274. https://doi.org/10.2307/3038040.

Behrens, B. C., Yokoyama, Y., Miyairi, Y., Sproson, A. D., Yamane, M., Jimenez-Espejo, F. J., McKay, R. M., Johnson, K. M., Escutia, C., & Dunbar, R. B. (2022). Beryllium isotope variations recorded in the Adélie Basin, East Antarctica reflect Holocene changes in ice dynamics, productivity, and scavenging efficiency. *Quaternary Science Advances*, **7**, 100054. https://doi.org/10.1016/j.qsa.2022.100054.

Bentley, M. J., Hodgson, D. A., Smith, J. A., Cofaigh, C. Ó., Domack, E. W., Larter, R. D., Roberts, S. J., Brachfeld, S., Leventer, A., Hjort, C., Hillenbrand, C.-D., & Evans, J. (2009). Mechanisms of Holocene palaeoenvironmental change in the Antarctic Peninsula region. *The Holocene*, **19**(1), 51–69. https://doi.org/10.1177/0959683608096603.

Berg, S., Melles, M., Gore, D., Verkulich, S., & Pushina, Z. (2020). Postglacial evolution of marine and lacustrine water bodies in Bunger Hills. *Antarctic Science*, **32**(2), 107–129. https://doi.org/10.1017/S0954102019000476.

Berg, S., Melles, M., Hermichen, W.-D., McClymont, E. L., Bentley, M. J., Hodgson, D. A., & Kuhn, G. (2018). Evaluation of Mumiyo Deposits From East Antarctica as Archives for the Late Quaternary Environmental and Climatic History. *Geochemistry, Geophysics, Geosystems*, **20**(1), 260–276. https://doi.org/10.1029/2018GC008054.

Berg, S.,Wagner, B., Cremer, H., Leng, M.J., & Melles, M. (2010). Late Quaternary environmental and climate history of Rauer Group, East Antarctica. *Palaeogeography, Palaeoclimatology, Palaeoecology*. **297**(1), 201–213. https://doi.org/10.1016/j.palaeo.2010.08.002.

Berg, S., Wagner, B., White, D. A., Cremer, H., Bennike, O., & Melles, M. (2009). New marine core record of Late Pleistocene glaciation history, Rauer Group, East Antarctica. *Antarctic Science*, **21**(3), 299-300. https://doi.org/10.1017/S0954102009001886.

Berg, S., White, D. A., Jivcov, S., Melles, M., Leng, M. J., Rethemeyer, J., Allen, C., Perren, B., Bennike, O., & Viehberg, F. (2019). Holocene glacier fluctuations and environmental changes in subantarctic South Georgia inferred from a sediment record from a coastal inlet. *Quaternary Research*, **91**(1), 132–148. https://doi.org/10.1017/qua.2018.85.

Bergstrom, D.M., Stewart, G.R., Selkirk, P.M., & Schmidt, S. (2002). ^15^N natural abundance of fossil peat reflects the influence of animal-derived nitrogen on vegetation. *Oecologia*, **130**, 309–314. https://doi.org/10.1007/s004420100807

Bird, M. I., Chivas, A. R., Radnell, C. J., & Burton, H. R. (1991). Sedimentological and stable-isotope evolution of lakes in the Vestfold Hills, Antarctica. *Palaeogeography, Palaeoclimatology, Palaeoecology*, **84**(1-4), 109-130. https://doi.org/10.1016/0031-0182(91)90039-T.

Björck, S., Håkansson, H., Olsson, S., Barnekow, L., & Janssens, J.A. (1993). Palaeoclimatic studies in South Shetland Islands, Antarctica, based on numerous stratigraphic variables in lake sediments. *Journal of Paleolimnology*, **8**, 233e272. https://doi.org/10.1007/BF00177858.

Björck, S., Håkansson, H., Zale, R., Karlén, W., & Jönsson, B. (1991a). A late Holocene lake sediment sequence from Livingston Island, South Shetland Islands, with palaeoclimatic implications. *Antarctic Science*, **3**(1), 61-72. https://doi.org/10.1017/S095410209100010X.

Björck, S., Malmer, N., Hjort, C., Sandgren, P., Ingólfsson, O., Wallén, B., Smith, R. I. L., & Jonsson, B. L. (1991b). Stratigraphic and Palaeoclimate studies of a 5500-year-old moss bank on Elephant Island, Antarctica. *Arctic and Alpine Research*, **23**(4), 361e374.

Björck, S., Olsson, S., Ellis-Evans, C., Håkansson, H., Humlum, O., & de Lirio, J. M. (1996). Late Holocene palaeoclimatic records from lake sediments on James Ross Island, Antarctica. *Palaeogeography, Palaeoclimatology, Palaeoecology*, **121**(3-4), 195–220. https://doi.org/10.1016/0031-0182(95)00086-0.

Björck, S., Sandgren, P., & Zale, R. (1991c). Late Holocene tephrochronology of the northern Antarctic Peninsula. *Quaternary Research*, **36**(3), 322-328. https://doi.org/10.1016/0033-5894(91)90006-Q.

Bohaty, S. M., & Harwood, D. M. (1998). Southern Ocean Pliocene paleotemperature variation from high resolution silicoflagellate biostratigraphy. *Marine Micropaleontology*, **33**, 241–272.

Brey T., Gutt J., Mackensen A., & Starmans A. (1998). Growth and productivity of the high Antarctic Bryozoan *Melicerita obliqua*. *Marine Biology*, **132**, 327–333. https://doi.org/10.1007/s002270050398.

Brey, T., Voigt, M., Jenkins, K., & Ahn, I.-Y. (2011). The bivalve *Laternula elliptica* at King George Island — A biological recorder of climate forcing in the West Antarctic Peninsula region. *Journal of Marine Systems*, **88**(4), 542–552. https://doi.org/10.1016/j.jmarsys.2011.07.004.

Brachfeld, S., Domack, E., Kissel, C., Laj, C., Leventer, A., Ishman, S., Gilbert, R., Camerlenghi, A., & Eglinton, L. B. (2003). Holocene history of the Larsen-A Ice Shelf constrained by geomagnetic paleointensity dating. *Geology*, **31**(9), 749–752. https://doi.org/10.1130/G19643.1.

Borchers, A., Dietze, E., Kuhn, G., Esper, O., Voigt, I., Hartmann, K., & Diekmann, B. (2016). Holocene ice dynamics and bottom-water formation associated with Cape Darnley polynya activity recorded in Burton Basin, East Antarctica. *Marine Geophysical Research*, **37**, 49–70. https://doi.org/10.1007/s11001-015-9254-z.

Browne, I. M., Moy, C. M., Riesselman, C. R., Neil, H. L., Curtin, L. G., Gorman, A. R., & Wilson, G. S. (2017). Late Holocene intensification of the westerly winds at the subantarctic Auckland Islands (51° S), New Zealand. *Climate of the Past*, **13**, 1301–1322. https://doi.org/10.5194/cp-13-1301-2017,%202017.

Campagne, P., Crosta, X., Houssais, M., Swingedouw, D., Schmidt, S., Martin, A., Devred, E., Capo, S., Marieu, V., Closset, I., & Massé, G. (2015). Glacial ice and atmospheric forcing on the Mertz Glacier Polynya over the past 250 years. *Nature Communications*, **6**, 6642. https://doi.org/10.1038/ncomms7642.

Chapelin-Viscardi, J.-D., Voisin, J.-F., Ponel, P., & Van der Putten, N. (2010). *Pachnobium dreuxin*. g., n. sp., ses occurrences modernes et fossiles sur l’archipel Crozet (Coléoptère Curculionidae Ectemnorrhininae). Annales de La Societe Entomologique de France. *Societe Entomologique de France*, **46**(1-2), 125–131. https://doi.org/10.1080/00379271.2010.10697647.

Charman, D. J., Amesbury, M. J., Roland, T. P., Royles, J., Hodgson, D A., Convey, P., & Griffiths, H. (2018). Spatially coherent late Holocene Antarctic Peninsula surface air temperature variability. *Geology*, **46**(12) 1071–1074. https://doi.org/10.1130/G45347.1.

Chen, Q., Liu, X., Nie, Y., & Sun, L. (2013). Using visible reflectance spectroscopy to reconstruct historical changes in chlorophyll a concentration in East Antarctic ponds. *Polar Research*, **32**, 19932. https://doi.org/10.3402/polar.v32i0.19932.

Christ, A. J., Talaia-Murray, M., Elking, N., Domack, E. W., Leventer, A., Lavoie, C., Brachfeld, S., Yoo, K.-C., Gilbert, R., Jeong, S.-M., Petrushak, S., Wellner, J., & the LARISSA Group. (2015). Late Holocene glacial advance and ice shelf growth in Barilari Bay, Graham Land, west Antarctic Peninsula. *Geological Society of America Bulletin*, **127**(1-2), 297–315. https://doi.org/10.1130/B31035.1

Clarke, L. J., Robinson, S. A., Hua, Q., Ayre, D. J., & Fink, D. (2012). Radiocarbon bomb spike reveals biological effects of Antarctic climate change. *Global Change Biology*, **18**, 301–310. https://doi.org/10.1111/j.1365-2486.2011.02560.x.

Cleary, A. C., Hoffman, J. I., Forcada, J., Lydersen, C., Lowther, A. D., & Kovacs, K. M. (2021). 50,000 years of ice and seals: Impacts of the Last Glacial Maximum on Antarctic fur seals. *Ecology and Evolution*, **11**(20), 14003–14011. https://doi.org/ 10.1002/ece3.8104.

Clucas, G., Dunn, M., Dyke, G., Emslie, S. D., Levy, H., Naveen, R., Polito, M. J., Pybus, O. G., Rogers, A. D., & Hart, T. (2014). A reversal of fortunes: climate change ‘winners’ and ‘losers’ in Antarctic Peninsula penguins. *Scientific Reports*, **4**, 5024. https://doi.org/10.1038/srep05024.

Collins, G. E., Hogg, I. D., Convey, P., Sancho, L. G., Cowan, D. A., Lyons, W. B., Adams, B. J., Wall, D. H., & Green, T. G. A. (2020). Genetic diversity of soil invertebrates corroborates timing estimates for past collapses of the West Antarctic Ice Sheet. *Proceedings of the National Academy of Sciences of the United States of America*, **117**(36), 22293–22302. https://doi.org/10.1073/pnas.2007925117.

Coolen, M. J. L., Muyzer, G., Rijpstra, W. I. C., Schouten, S., Volkman, J. K., & Sinninghe Damsté, J.S. (2004a). Combined DNA and lipid analyses of sediments reveal changes in Holocene haptophyte and diatom populations in an Antarctic lake. *Earth and Planetary Science Letters*, **223**(1-2), 225–239. https://doi.org/10.1016/j.epsl.2004.04.014.

Coolen, M. J. L., Hopmans, E. C., Rijpstra, W. I. C., Muyzer, G., Schouten, S, Volkman, J. K., & Sinninghe Damsté, J. S. (2004b). Evolution of the methane cycle in Ace Lake (Antarctica) during the Holocene: response of methanogens and methanotrophs to environmental change. *Organic Geochemistry*, **35**(10), 1151–1167. https://doi.org/10.1016/j.orggeochem.2004.06.009.

Cooper, A. K. & O’Brien, P. E. (2004). Leg 188 synthesis: transitions in the glacial history of the Prydz Bay region, East Antarctica, from ODP drilling. In Cooper, A. K., O’Brien, P. E. & Shipboard Scientific Party. Prydz Bay–Cooperation Sea, Antarctica: Glacial History and Paleoceanography Sites 1165–1167. Proceedings of the Ocean Drilling Program, Scientific Results, 188.

Convey, P., Hopkins, D. W., Roberts, S. J., & Tyler, A. N. (2011). Global southern limit of flowering plants and moss peat accumulation. *Polar Research*, **30**, 8929. https://doi.org/10.3402/polar.v30i0.8929.

Cremer, H., Gore, D., Melles, M., & Roberts, D. (2003). Palaeoclimatic significance of late Quaternary diatom assemblages from southern Windmill Islands, East Antarctica, *Palaeogeography, Palaeoclimatology, Palaeoecology*, **195**(3–4), 261–280. https://doi.org/10.1016/S0031-0182(03)00361-4.

Cromer, L., Gibson, J. A. E., McInnes, S. J., & Agius, J.T. (2008). Tardigrade remains from lake sediments. *Journal of Paleolimnology*, **39**,143-150. https://doi.org/10.1007/s10933-007-9102-5.

Cromer, L., Gibson, J. A. E., Swadling, K. M., & Hodgson, D. A. (2006). Evidence for a Lacustrine Faunal Refuge in the Larsemann Hills, East Antarctica, during the Last Glacial Maximum. *Journal of Biogeography*, **33**(7), 1314–1323. https://doi.org/10.1111/j.1365-2699.2006.01490.x.

Cromer, L., Gibson, J. A. E., Swadling, K. M., & Ritz, D. A. (2005). Faunal microfossils: Indicators of Holocene ecological change in a saline Antarctic lake. *Palaeogeography, Palaeoclimatology, Palaeoecology*, **221**, 83–97. https://doi.org/10.1016/j.palaeo.2005.02.005.

Cronin, K. E., Walker, S. E., Mann, R., Chute, A. S., Chase Long, M., & Bowser, S. S. (2020). Growth and longevity of the Antarctic scallop *Adamussium colbecki* under annual and multiannual sea ice. *Antarctic Science*, **32**(6), 466–475. https://doi.org/10.1017/S0954102020000322.

Crosta, X., Debret, M., Denis, D., Courty, M. A., and Ther, O. (2007). Holocene long- and short-term climate changes off Adélie Land, East Antarctica. *Geochemistry, Geophysics, Geosystems*, **8**, Q11009. https://doi.org/10.1029/2007GC001718.

Crosta, X., Kohfeld, K. E., Bostock, H. C., Chadwick, M., Du Vivier, A., Esper, O., Etourneau, J., Jones, J., Leventer, A., Müller, J., Rhodes, R. H., Allen, C. S., Ghadi, P., Lamping, N., Lange, C., Lawler, K.-A., Lund, D., Marzocchi, A., Meissner, K. J., Menviel, L., … Yang, J. (2022). Antarctic sea ice over the past 130,000 years, Part 1: A review of what proxy records tell us. *EGUsphere,* [preprint]. https://doi.org/10.5194/egusphere-2022-99.

Cunningham, W. L., Leventer, A., Andrews, J.T., Jennings, A.E., & Licht, K.J. (1999). Late Pleistocene-Holocene marine conditions in the Ross Sea, Antarctica: evidence from the diatom record. *The Holocene*, **9**(2), 129–139. https://doi.org/10.1191/095968399675624796.

de Bruyn, M., Hall, B. L., Chauke, L. F., Baroni, C., Koch, P. L., & Hoelzel, A. R. (2009). Rapid Response of a Marine Mammal Species to Holocene Climate and Habitat Change. *PLoS Genetics*, **5**(7), e1000554. https://doi.org/10.1371/journal.pgen.1000554.

de Bruyn, M., Pinsky, M.L., Hall, B., Koch, P., Baroni, C., & Hoelzel, A. R. (2014). Rapid increase in southern elephant seal genetic diversity after a founder event. *Proceedings of the Royal Society of London B: Biological Sciences*, **281**(1779), 20133078. https://doi.org/10.1098/rspb.2013.3078.

Del Valle, R. A., Montalti, D., & Inbar, M. (2002). Mid-Holocene macrofossil-bearing raised marine beaches at Potter Peninsula, King George Island, South Shetland Islands. *Antarctic Science*, **14**, 263–269. https://doi.org/10.1017/S0954102002000081.

Denis, D., Crosta, X., Barbara, L., Massé, G., Renssen, H., Ther, O., & Giraudeau, J. (2010). Sea ice and wind variability during the Holocene in East Antarctica: insight on middle–high latitude coupling. *Quaternary Science Reviews*, **29**(27-28), 3709–3719. https://doi.org/10.1016/j.quascirev.2010.08.007.

Díaz, A., Gérard, K., González-Wevar, C., Maturana, C., Féral, J.-P., David, B., Saucède, T., & Poulin, E. (2018). Genetic structure and demographic inference of the regular sea urchin *Sterechinus neumayeri* (Meissner, 1900) in the Southern Ocean: The role of the last glaciation. *PLoS ONE*, **13**(6), e0197611. https://doi.org/10.1371/journal.pone.0197611.

Domack, E. W. (2002). A synthesis for site 1098: Palmer Deep. In: P. F. Barker, A. Camerlenghi, G. D. Acton, & A. T. S. Ramsay (Eds). Proceedings of the Ocean Drilling Program, Scientific Results. Ocean Drilling Program, Texas A&M University.

Domack, E. W., Jull, A. J. T., & Donahue, D. J. (1991). 42. HOLOCENE CHRONOLOGY FOR THE UNCONSOLIDATED SEDIMENTS AT HOLE 740A: PRYDZ BAY, EAST ANTARCTICA. In: Barron, J., Larsen B., Baldauf, J., Allbert, C., Berkowltz, S., Caulet, J.-P., Chambers, S., Cooper, A., Cranston, R., Dorn, W., Ehrmann, W., Fox, R., Fryxell, G., Hambrey, M., Huber, B., Jenkins, C., Kang, S.-H., Keating, B., Mehl, K., … Wei, W. (Eds). Proceedings of the Ocean Drilling Program, Scientific Results. Ocean Drilling Program, Texas A&M University.

Domack, E. W., Leventer, A., Root, S., Ring, J., Williams, E., Carlson, D., Hirshorn, E., Wright, W., Gilbert, R., & Burr, G. (2003). Marine sedimentary record of natural environmental variability and recent warming in the Antarctic Peninsula. In: Domack, E. W., Leventer, A., Burnett, A., Bindschadler, R., Convey, P., & Kirby, M. (Eds). Antarctic Peninsula climate variability: historical and paleoenvironmental perspectives. Antarctic Research Series 7. (pp. 205-222). American Geophysical Union.

Domack, E., O'Brien, P., Harris, P., Taylor, F., Quilty, P., Santis, L., & Raker, B. (1998). Late Quaternary sediment facies in Prydz Bay, East Antarctica and their relationship to glacial advance onto the continental shelf. *Antarctic Science*, **10**(3), 236–246. https://doi.org/10.1017/S0954102098000339.

Dunbar, G. B., Naish, T. R., Barrett, P. J., Fielding, C. R. & Powell, R.D. (2008). Constraining the amplitude of late Oligocene bathymetric changes in Western Ross Sea during orbitally-induced oscillations in the East Antarctic Ice Sheet: (1) Implications for glacimarine sequence stratigraphic models. *Palaeogeography, Palaeoclimatology, Palaeoecology*, **260**(1–2), 50–65.

Ehrmann, W. U. (1991). Implications of sediment composition on the southern Kerguelen Plateau for paleoclimate and depositional environment. In: Barron, J., Larsen B., Baldauf, J., Allbert, C., Berkowltz, S., Caulet, J.-P., Chambers, S., Cooper, A., Cranston, R., Dorn, W., Ehrmann, W., Fox, R., Fryxell, G., Hambrey, M., Huber, B., Jenkins, C., Kang, S.-H., Keating, B., Mehl, K., … Wei, W. (Eds). Proceedings of the Ocean Drilling Program, Scientific Results. Ocean Drilling Program, Texas A&M University.

Ehrmann, W. (1998). Implications of late Eocene to early Miocene clay mineral assemblages in McMurdo Sound (Ross Sea, Antarctica) on paleoclimate and ice dynamics. *Palaeogeography, Palaeoclimatology, Palaeoecology*, **139**(3-4), 213-231. https://doi.org/10.1016/S0031-0182(97)00138-7.

Emslie, S. D. (2021). Ancient Adélie penguin colony revealed by snowmelt at Cape Irizar, Ross Sea, Antarctica. *Geology*, **49**(2), 145–149. https://doi.org/10.1130/G48230.1.

Emslie, S. D., Coats, L., & Licht, K. (2007). A 45,000 yr record of Adélie penguins and climate change in the Ross Sea, Antarctica. *Geology*, **35**(1), 61–64. https://doi.org/10.1130/G23011A.1.

Emslie, S. D., & Patterson, W. P. (2007). Abrupt recent shift in δ13C and δ15N values in Adélie penguin eggshell in Antarctica. *Proceedings of the National Academy of Sciences of the United States of America*, **104**, 11666–11669.

Etourneau, J., Collins, L. G., Willmott, V., Kim, J. H., Barbara, L., Leventer, A., Schouten, S., Damste, J. S. S., Bianchini, A., Klein, V., Crosta, X., & Massé, G. (2013). Holocene climate variations in the western Antarctic Peninsula: evidence for sea ice extent predominantly controlled by changes in insolation and ENSO variability. *Climate of the Past*, **9**, 1431–1446. https://doi.org/10.5194/cp-9-1431-2013,%202013.

Ficetola, G. F., Poulenard, J., Sabatier, P., Messager, E., Gielly, L., Leloup, A., Etienne, D., Bakke, J., Malet, E., Fanget, B., Støren, E., Reyss, J.-L., Taberlet, P., & Arnaud, F. (2018). DNA from lake sediments reveals long-term ecosystem changes after a biological invasion. *Science Advances*, **4**(5), eaar4292. https://doi.org/10.1126/sciadv.aar4292.

Fleming, C. A., Mildenhall, D. C., & Moar, N. T. (1976). Quaternary sediments and plant microfossils from Enderby Islands, Auckland Islands. *Journal of the Royal Society of New Zealand*, **6**, 433–458. https://doi.org/10.1080/03036758.1976.10421484.

Foster, L. C., Pearson, E. J., Juggins, S., Hodgson, D. A., Saunders, K. M., Verleyen, E., & Roberts, S. J. (2016).

Development of a regional glycerol dialkyl glycerol tetraether (GDGT)–temperature calibration for Antarctic and sub-Antarctic lakes. *Earth and Planetary Science Letters*, **433**, 370–379, https://doi.org/10.1016/j.epsl.2015.11.018.

Frugone, M. J., Lowther, A., Noll, D., Ramos, B., Pistorius, P., Dantas, G. P. M., Petry, M. V., Bonadonna, F., Steinfurth, A., Polanowski, A., Raya Rey, A., Lois, N. A., Pütz, K., Trathan, P., Wienecke, B., Poulin, E., & Vianna, J. A. (2018). Contrasting phylogeographic pattern among *Eudyptes* penguins around the Southern Ocean. *Scientific Reports*, **8**(1), 17481.

Gasparon, M., Ehrler, K., Matschullat, J., & Melles, M. (2007). Temporal and spatial variability of geochemical backgrounds in the Windmill Islands, East Antarctica: Implications for climatic changes and human impacts. *Applied Geochemistry*, **22**(5), 888–905. https://doi.org/10.1016/j.apgeochem.2006.12.018.

Gibson, J., & Bayly, I. (2007). New insights into the origins of crustaceans of Antarctic lakes. *Antarctic Science*, **19**(2), 157-164. https://doi.org/10.1017/S0954102007000235.

Gibson, J. A. E., Cromer, L., Agius, J. T., McInnes, S. J., & Marley, N. J. (2007). Tardigrade eggs and exuviae in Antarctic lake sediments: insights into Holocene dynamics and origins of the fauna. *Journal of limnology*, **66**(Suppl. 1), 65-71 https://doi.org/10.4081/jlimnol.2007.s1.65.

Gibson, J., Paterson, K., White, C., & Swadling, K. (2009). Evidence for the continued existence of Abraxas Lake, Vestfold Hills, East Antarctica during the Last Glacial Maximum. *Antarctic Science*, **21**(3), 269-278. https://doi.org/10.1017/S095410200900180.

Gibson, J. A. E., & Zale, R. (2006) Holocene development of the fauna of Lake Boeckella, northern Antarctic Peninsula. *The Holocene*, **16**, 625-634. https://doi.org/10.1191/0959683606hl959rp.

Goodwin, I. D. (1993). Holocene Deglaciation, Sea-Level Change, and the Emergence of the Windmill Islands, Budd Coast, Antarctica. *Quaternary Research*, **40**(1), 70–80. https://doi.org/10.1006/qres.1993.1057.

Govil, P., Mazumder, A., Asthana, R., Tiwari, A., & Mishra, R. (2016). Holocene climate variability from the lake sediment core in Schirmacher Oasis region, East Antarctica: Multiproxy approach. *Quaternary International*, **425**, 453-463. https://doi.org/10.1016/j.quaint.2016.09.032.

González-Wevar, C. A., Chown, S. L., Morley, S., Coria, N., Saucéde, T., & Poulin, E. (2016). Out of Antarctica: quaternary colonization of sub-Antarctic Marion Island by the limpet genus *Nacella* (Patellogastropoda: Nacellidae). *Polar Biology*, **39**(1), 77–89. https://doi.org/10.1007/s00300-014-1620-9.

Gulick, S. P. S., Shevenell, A. E., Montelli, A., Fernandez, R., Smith, C., Warny, S., Bohaty, S. M., Sjunneskog, C., Leventer, A., Frederick, B., & Blankenship, D. D. (2017). Initiation and long-term instability of the East Antarctic Ice Sheet. *Nature*, **552**, 225–229. https://doi.org/10.1038/nature25026.

Hall, B., Hoelze,l A., Baroni, C., Denton, G., Le Boeuf, B., Overturf, B., & Töpf, A. (2006). Holocene elephant seal distribution implies warmer-than-present climate in the Ross Sea. *Proceedings of the National Academy of Sciences of the United States of America*, **103**(27), 10213–10217. https://doi.org/10.1073/pnas.0604002103.

Hellberg, M. E., Aronson, R. B., Smith, K. E., Duhon, M. I., Ahyong, S. T., Lovrich, G. A., Thatje, S., & McClintock, J. B. (2019). Population expansion of an Antarctic king crab? *Frontiers of Biogeography*, **11**(3). https://doi.org/10.21425/F5FBG43165.

Hemer, M. A., & Harris, P. T. (2003). Sediment core from beneath the Amery Ice Shelf, East Antarctica, suggests mid-Holocene ice-shelf retreat. *Geology*, **31**(2), 127–130. https://doi.org/10.1130/0091-7613(2003)031<0127:SCFBTA>2.0.CO;2

Hendy, C. H. (2000). Late Quaternary lakes in the McMurdo Sound region of Antarctica. *Geografiska Annaler: Series A, Physical Geography*, **82**(2-3), 411-432. https://doi.org/10.1111/j.0435-3676.2000.00131.x.

Heroy, D. C., Sjunneskog, C., & Anderson, J. B. (2008). Holocene climate change in the Bransfield Basin, Antarctic Peninsula: Evidence from sediment and diatom analysis. *Antarctic Science*, **20**(1), 69-87. https://doi.org/10.1017/S0954102007000788.

Heywood, R. B. (1977). A limnological survey of the Ablation Point area, Alexander Island, Antarctica. *Philosophical Transactions of the Royal Society B: Biological Sciences*, **279**, 39-54. https://doi.org/10.1098/rstb.1977.0070.

Hillenbrand, C.-D., Fütterer, D.K., Grobe, H., & Frederichs, T. (2002). No evidence for a Pleistocene collapse of the West Antarctic Ice Sheet from continental margin sediments recovered in the Amundsen Sea. *Geo-Marine Letters*, **22**, 51–59. https://doi.org/10.1007/s00367-002-0097-7

Hillenbrand, C.-D., Grobe, H., Diekmann, B., Kuhn, G., & Fütterer, D. K. (2003). Distribution of clay minerals and proxies for productivity in surface sediments of the Bellingshausen and Amundsen seas (West Antarctica) – Relation to modern environmental conditions. *Marine Geology*, **193**(3-4), 253–271. https://doi.org/10.1016/S0025-3227(02)00659-X.

Hillenbrand, C.-D., Kuhn, G., Smith, J. A., Gohl, K., Graham, A. G. C., Larter, R. D., Klages, J. P., Downey, R., Moreton, S. G., Forwick, M., & Vaughan, D. G. (2013). Grounding-line retreat of the West Antarctic Ice Sheet from inner Pine Island Bay. *Geology*, **41**(1): 35–38. https://doi.org/10.1130/G33469.1.

Hillenbrand, C.-D., Smith, J., Hodell, D., Greaves, M., Poole, C. R., Kender, S., Williams, M., Andersen, T. J., Jernas, P. E., Elderfield, H., Klages, J. P., Roberts, S. J., Gohl, K., Larter, R. D., & Kuhn, G. (2017). West Antarctic Ice Sheet retreat driven by Holocene warm water incursions. *Nature*, **547**, 43–48. https://doi.org/10.1038/nature22995.

Hodgson, D. A., & Convey, P. (2005). A 7000-year Record of Oribatid Mite Communities on a Maritime-Antarctic Island: Responses to Climate Change. *Arctic, Antarctic, and Alpine Research*, **37**(2), 239–245. https://doi.org/10.1657/1523-0430(2005)037[0239:AYROOM]2.0.CO;2

Hodgson, D. A., Roberts, S. J., Bentley, M.  J., Carmichael, E. L., Smith, J. A., Verleyen, E., Vyverman, W., Geissler, P., Leng, M. J., & Sanderson, D. C. W. (2009). Exploring former subglacial Hodgson Lake, Antarctica. Paper II: palaeolimnology.

*Quaternary Science Reviews*, **28**(23–24), 2310–2325. https://doi.org/10.1016/j.quascirev.2009.04.014.

Hodgson, D. A., Whitehouse, P. L.  De Cort, G., Berg, S, Verleyen, E., Tavernier, I., Roberts, S. J., Vyverman, W., Sabbe, K., O'Brien, P. (2016). Rapid early Holocene sea-level rise in Prydz Bay, East Antarctica. *Global and Planetary Change*, **139**, 128-140. https://doi.org/10.1016/j.gloplacha.2015.12.020.

Hodgson, D., Verleyen, E., Sabbe, K., Squier, A., Keely, B., Leng, M., Saunders, K.M., & Vyverman, W. (2005). Late Quaternary climate-driven environmental change in the Larsemann Hills, East Antarctica, multi-proxy evidence from a lake sediment core. *Quaternary Research*, **64**(1), 83-99. https://doi.org/10.1016/j.yqres.2005.04.002.

Hodgson, D., Verleyen, E., Squier, A., Sabbe, K., Keely, B. J., Saunders, K. M., & Vyverman, W. (2006). Interglacial environments of coastal east Antarctica: comparison of MIS 1 (Holocene) and MIS 5e (Last Interglacial) lake-sediment records. *Quaternary Science Reviews*, **25**(1-2), 179-197. https://doi.org/10.1016/j.quascirev.2005.03.004.

Huang, H., Gutjahr, M., Eisenhauer, A., & Kuhn, G. (2020). No detectable Weddell Sea Antarctic Bottom Water export during the Last and Penultimate Glacial Maximum. *Nature Communication*, **11**, 424. https://doi.org/10.1038/s41467-020-14302-3.

Huang, T., Sun, L., Wang, Y., & Kong, D. (2011a). Late Holocene Adélie penguin population dynamics at Zolotov Island, Vestfold Hills, Antarctica. *Journal of Paleolimnology*, **45**, 273–285. https://doi.org/10.1007/s10933-011-9497-x.

Huang, T., Sun, L., Wang, Y., Liu, X., Zhu, R. (2009a). Penguin population dynamics for the past 8500 years at Gardner Island, Vestfold Hills. *Antarctic Science*, **21**, 571–578. https://doi.org/10.1017/S0954102009990332.

Huang, J., Sun, L., Wang, X., Wang, Y., & Huang, T. (2011b). Ecosystem evolution of seal colony and the influencing factors in the 20th century on Fildes Peninsula, West Antarctica. *Journal of Environmental Sciences*, **23**(9), 1431–1436. https://doi.org/10.1016/S1001-0742(10)60601-8.

Huang, T., Sun, L., Wang, Y., Zhu, R. (2009b). Penguin occupation in the Vestfold Hills. *Antarctic Science*, **21**, 131–134. https://doi.org/10.1017/S095410200800165X.

Huang, T., Yang, L., Chu, Z., Sun, L., & Yin, X. (2016). Geochemical record of high emperor penguin populations during the Little Ice Age at Amanda Bay, Antarctica. *Science of the Total Environment*, **565**, 1185–1191. https://doi.org/10.1016/j.scitotenv.2016.05.166.

Ingólfsson, Ó, Hjort, C, Berkman, P., Björck, S., Colhoun, E., Goodwin, I., Hall, B., Hirakawa, K., Möller P., & Prentice, M. L. (1998). Antarctic glacial history since the Last Glacial Maximum: An overview of the record on land. *Antarctic Science*, **10**(3), 326-344. doi:10.1017/S095410209800039X.

International Ocean Discovery Program (IODP) (2015). Archive of Geosample Information from Scientific Ocean Drilling. NOAA National Centers for Environmental Information. https://doi.org/10.7289/V57M05XN. Accessed 6 May 2022.

Janko, K., Lecointre, G., DeVriess, A., Couloux, A., Cruaud C., & Marshall, C. (2007). Did glacial advances during the Pleistocene influence differently the demographic histories of benthic and pelagic Antarctic shelf fishes? – Inferences from intraspecific mitochondrial and nuclear DNA sequence diversity. *BMC Evolutionary Biology*, **7**, 220. https://doi.org/10.1186/1471-2148-7-220.

Jones, V. J., Hodgson, D. A., & Chepstow-Lusty, A. (2000). Palaeolimnological evidence for marked Holocene environmental changes on Signy Island, Antarctica. *The Holocene*, **10**, 43e60. https://doi.org/10.1191/095968300673046662.

Keenan, H. (1995). Modern and fossil terrestrial and freshwater habitats on Subantarctic Macquarie Island. PhD thesis, Macquarie University, Australia.

Kelley, J. L., Peyton, J. T., Fiston-Lavier, A.-S., Teets, N. M., Yee, M.-C., Johnston, J. S., Bustamante, C. D., Lee, R. E., & Denlinger, D. L. (2014). Compact genome of the Antarctic midge is likely an adaptation to an extreme environment. *Nature Communications*, **5**, 4611. https://doi.org/10.1038/ncomms5611

Konfirst, M. A., Scherera, R. P., Hillenbrand, C.-D., & Kuhn, G. (2012). A marine diatom record from the Amundsen Sea — Insights into oceanographic and climatic response to the Mid-Pleistocene Transition in the West Antarctic sector of the Southern Ocean. *Marine Micropaleontology*, **92-93**, 40–51. https://doi.org/10.1016/j.marmicro.2012.05.001.

Kim, S.-Y., Lim, D., Rebolledo, L., Park, T., Esper, O., Muñoz, P., La, H. S., Kim, T. W., & Lee, S. (2021). A 350-year multiproxy record of climate-driven environmental shifts in the Amundsen Sea Polynya, Antarctica. *Global and Planetary Change*, **205**, 103589.

King, T. M., Rosenheim, B. E., Post, A. L., Gabris, T., Burt, T., & Domack, E. W. (2018). Large-Scale Intrusion of Circumpolar Deep Water on Antarctic Margin Recorded by Stylasterid Corals. *Paleoceanography and Paleoclimatology*, **33**(11), 1306–1321.

Kulbe, T. (1997). The late Quaternary climatic and environmental history of Bunger Oasis, East Antarctica. Reports on Polar Research 254, Alfred Wegener Institute for Polar and Marine Research, Bremerhaven, 130 pp.

Kulbe, T., Melles, M., Verkulich, S. R., & Pushina, Z. V. (2001). East Antarctic Climate and Environmental Variability over the Last 9400 Years Inferred from Marine Sediments of the Bunger Oasis. *Arctic, Antarctic, and Alpine Research*, **33**(9), 223–230. https://doi.org/10.1080/15230430.2001.12003425.

Lambert, D. M., Ritchie, P. A., Millar, C. D., Holland, B., Drummond, A. J., & Baroni, C. (2002). Rates of evolution in ancient DNA from Adelie penguins. *Science*, **295**, 2270–2273. https://doi.org/10.1126/science.1068105.

Lau, S. C. Y., Strugnell, J. M., Sands, C. J., Silva, C. N. S., & Wilson, N. G. (2021). Evolutionary innovations in Antarctic brittle stars linked to glacial refugia. *Ecology and Evolution*, **11**(23), 17428–17446. https://doi.org/10.1002/ece3.8376.

Leventer, A., Domack, E., Dunbar, R., Pike., J., Stickley, C., Maddison, E., Branchfeld, S., Manley, P., & McClennen, C. (2006). Marine sediment record from the East Antarctic margin reveals dynamics of ice sheet recession. *Geological Society of America Today*, **16**(12), 4–10. http://dx.doi.org/10.1130/GSAT01612A.1.

Leventer, A., Domack, E. W., Ishman, S. E., Brachfeld, S., McClennen, C. E., & Manley, P. (1996). Productivity cycles of 200–300 years in the Antarctic Peninsula region: Understanding linkages among the sun, atmosphere, oceans, sea ice, and biota. *Geological Society of America Bulletin*, **108**(12), 1626–1644. https://doi.org/10.1130/0016-7606(1996)108<1626:PCOYIT>2.3.CO;2.

Leventer, A., Dunbar, R. B., & DeMaster, D. J. (1993). Diatom Evidence for Late Holocene Climatic Events in Granite Harbor, Antarctica. *Paleoceanography and Paleoclimatology*, **8**(3), 373–386. https://doi.org/10.1029/93PA00561.

Li, C., Zhang, Y., Li, J., Kong, L., Hu, H., Pan, H., Xu, L., Deng, Y., Li, Q., Jin, L., Yu, H., Chen, Y., Liu, B., Yang, L., Liu, S., Zhang, Y., Lang, Y., Xia, J., He, W., … Zhang, G. (2014). Two Antarctic penguin genomes reveal insights into their evolutionary history and molecular changes related to the Antarctic environment. *GigaScience*, **3**(1), 27. https://doi.org/10.1186/2047-217X-3-27.

Loisel, J., Yu, Z., Beilman, D. W., Kaiser, K., & Parnikoza, I. (2017). Peatland Ecosystem Processes in the Maritime Antarctic During Warm Climates. *Scientific Reports*, **7**, 12344. https://doi.org/10.1038/s41598-017-12479-0.

López-Quirós, A., Lobo, F. J., Duffy, M., Leventer, A., Evangelinos, D., Escutia, C., & Bohoyo, F. (2021). Late Quaternary high-resolution seismic stratigraphy and core-based paleoenvironmental reconstructions in Ona Basin, southwestern Scotia Sea (Antarctica). *Marine Geology*, **439**, 106565. https://doi.org/10.1016/j.margeo.2021.106565.

Mahood, A. D. & Barron, J. A. (1996). Late Pliocene diatoms in a diatomite from Prydz Bay, East Antarctica. *Micropaleontology*, **42**(3), 285–302. https://doi.org/10.2307/1485876.

Mackintosh, A. N., Verleyen, E., O'Brien, P. E., White, D. A., Jones, R. S., McKay, R., Dunbar, R., Gore, D. B., Fink, D., Post, A. L., Miura, H., Leventer, A., Goodwin, I., Hodgson, D. A., Lilly, K., Crosta, X., Golledge, N. R., Wagner, B., Berg, S., … Masse, G. (2014). Retreat history of the East Antarctic Ice Sheet since the Last Glacial Maximum. *Quaternary Science Reviews*, **100**, 10–30. https://doi.org/10.1016/j.quascirev.2013.07.024.

Maddison, E. J., Pike, J., Leventer, A., Dunbar, R., Brachfeld, S., Domack, E. W., Manley, P., & McClennen, C. (2006). Post-glacial seasonal diatom record of the Mertz Glacier Polynya, East Antarctica. *Marine Micropaleontology*, **60**(1), 66–88. https://doi.org/10.1016/j.marmicro.2006.03.001.

Maturana, C. S., Segovia, N. I., González-Wevar, C. A., Díaz, A., Rosenfeld, S., Poulin, E., Jackson, J. A., & Convey, P. (2020). Evidence of strong small‐scale population structure in the Antarctic freshwater copepod *Boeckella poppei* in lakes on Signy Island, South Orkney Islands. *Limnology and Oceanography*, **65**(9), 2024–2040. https://doi.org/10.1002/lno.11435.

McClymont, E. L., Bentley, M. J., Hodgson, D. A., Spencer-Jones, C. L., Wardley, T., West, M. D., Croudace, I. W., Berg, S., Gröcke, D. R., Kuhn, G., Jamieson, S. S. R., Sime, L., & Phillips, R. A. (2022). Summer sea-ice variability on the Antarctic margin during the last glacial period reconstructed from snow petrel (*Pagodroma nivea*) stomach-oil deposits. *Climate of the Past*, **18**(2), 381–403. https://doi.org/10.5194/cp-18-381-2022.

McGlone, M. S. (2002). The Late Quaternary peat, vegetation and climate history of the Southern Oceanic Islands of New Zealand. *Quaternary Science Reviews*, **21**, 683-707. https://doi.org/10.1016/S0277-3791(01)00044-0.

McGlone, M. S., & Moar, N. T. (1997). Pollen–vegetation relationships on the subantarctic Auckland Islands, New Zealand. *Review of Palaeobotany and Palynology*, **96**, 317–338. https://doi.org/10.1016/S0034-6667(96)00058-9.

McGlone, M. S., Turney, C. S. M., Wilmshurst, J. M., Renwich, J., & Pahnke, K. (2010). Divergent trends in land and ocean temperature in the Southern Ocean over the past 18,000 years. *Nature Geoscience*, **3**, 622–626. https://doi.org/10.1038/ngeo931.

McGlone, M., Wilmshurst, J., & Meurk, C. (2007). Climate, fire, farming and the recent vegetation history of subantarctic Campbell Island. *Earth and Environmental Science Transactions of the Royal Society of Edinburgh*, 98(1), 71–84. https://doi.org/10.1017/S1755691007000060.

McGlone, M.S., Wilmshurst, J.M., & Wiser, S.K. (2000). Late-glacial and Holocene vegetation and climate change on Auckland Island, subantarctic New Zealand. *The Holocene*, **10**, 719–728. https://doi.org/10.1177%2F095968369700700101.

McMullen, K., Domack, E., Leventer, A., Olson, C., Dunbar, R., & Brachfeld, S. (2006). Glacial morphology and sediment formation in the Mertz Trough, East Antarctica. *Palaeogeography, Palaeoclimatology, Palaeoecology*, **231**(1-2), 169–180. https://doi.org/10.1016/j.palaeo.2005.08.004.

Melles, M., Kulbe, T., Verkulich, S. R., Pushina, Z. V., & Hubberten, H.-W. (1995): Late Pleistocene and Holocene environmental history of Bunger Hills, East Antarctica, as revealed by fresh-water and epishelf lake sediments. - In: Ricci, C. A. (Ed.), The Antarctic Region: Geological evolution and processes, Proceedings of the 7th International Symposium on Antarctic Earth Sciences (pp. 809-820). Siena: Terra Antartica Publication.

Melles, M., Verkulich, S., & Hermichen, W. (1994). Radiocarbon dating of lacustrine and marine sediments from the Bunger Hills, East Antarctica. *Antarctic Science*, **6**(3), 375-378. https://doi.org/10.1017/S095410209400057X.

Mezgec, K., Stenni, B., Crosta, X., Masson-Delmotte, V., Baroni, C., Braida, M., Ciardini, V., Colizza, E., Melis, R., Salvatore, M. C., Severi, M., Scarchilli, C., Traversi, R., Udisti, R., & Frezzotti, M. (2017). Holocene sea ice variability driven by wind and polynya efficiency in the Ross Sea. *Nature Communications*, **8**, 1334. https://doi.org/10.1038/s41467-017-01455-x.

Miller, W. R., & Heatwole, H. F. (2003) Tardigrades of the Sub-Antarctic: 5000 year old eggs from Marion Island. Abstracts, 9th International Symposium on Tardigrada, St Pete Beach, Florida: 46.

Minzoni, R. T., Anderson, J. B., Fernandez, R., & Wellner, J. S. (2015). Marine record of Holocene climate, ocean, and cryosphere interactions: Herbert Sound, James Ross Island, Antarctica. *Quaternary Science Reviews*, **129**, 239–259. https://doi.org/10.1016/j.quascirev.2015.09.009.

Moar, N. T. (1973). Late Pleistocene vegetation and environment in southern New Zealand. In: van Zinderen Bakker, E.M. (Ed.), Palaeoecology of Africa and the Surrounding Islands and Antarctica, Vol. 8 (pp. 179–198). Cape Town: Balkema.

Monien, P., Schnetger, B., Brumsack, H.-J., Hass, H. C., & Kuhn, G. (2011). A geochemical record of late Holocene palaeoenvironmental changes at King George Island (maritime Antarctica). An*tarctic Science*, **23**(3), 255–267. https://doi.org/10.1017/S095410201100006X

McMullin, R. M., Wing, S. R., & Reid, M. R. (2017). Ice fish otoliths record dynamics of advancing and retreating sea ice in Antarctica. *Limnology and Oceanography*, **62**(6), 2662–2673. https://doi.org/10.1002/lno.10597.

Naish, T., Powell, R., Levy, R., Wilson, G., Scherer, R., Talarico, F., Krissek, L., Niessen, F., Pompilio, M., Wilson, T., Carter, L., DeConto, R.,  Huybers, P., McKay, R., Pollard, D., Ross, J., Winter, D., Barrett, P., Browne, G., … Williams, T. (2009).

Obliquity-paced Pliocene West Antarctic ice sheet oscillations. *Nature*, **458**, 322–328. https://doi.org/10.1038/nature07867.

Noon, P. E., Leng, M. J., & Jones, V. J. (2003). Oxygen-isotope (d18O) evidence of Holocene hydrological changes at Signy Island, maritime Antarctica. *The Holocene*, **13**, 251-263. https://doi.org/10.1191/0959683603hl611rp.

O'Brien, P. E. & Harris, P. T. (1996). Patterns of glacial erosion and deposition in Prydz Bay and the past behaviour of the Lambert Glacier.*Papers and Proceedings of the Royal Society of Tasmania*, **130**(2), 79–85, https://doi.org/10.26749/rstpp.130.2.79.

Oliva, M., Antoniades, D., Serrano, E., Giralt, S., Liu, E., Granados, I., Pla-Rabes, S., Toro, M., Hong, S. G., & Vieira, G. (2019). The deglaciation of Barton Peninsula (King George Island, South Shetland Islands, Antarctica) based on geomorphological evidence and lacustrine records. *Polar Record*, **55**(3), 177-188. https://doi.org/10.1017/S0032247419000469.

Peck, V. L., Allen, C. S., Kender, S., McClymont, E. L., & Hodgson, D. A. (2015). Oceanographic variability on the West Antarctic Peninsula during the Holocene and the influence of upper circumpolar deep water. *Quaternary Science Reviews*, **119**, 54-65. https://doi.org/10.1016/j.quascirev.2015.04.002.

Peña, F., Poulin, E., Dantas, G. P., González-Acuña, D., Petry, M. V., & Vianna, J. A. (2014) Have historical climate changes affected gentoo penguin (*Pygoscelis papua*) populations in Antarctica? *PLoS ONE*, **9**, e95375. https://doi.org/10.1371/journal.pone.0095375.

Perren, B. B., Hodgson, D. A., Roberts, S. J., Sime, L., Nieuwenhuyze, W. V., Verleyen, E., & Vyverman, W. (2020). Southward migration of the Southern Hemisphere westerly winds corresponds with warming climate over centennial timescales. *Communications Earth & Environment*, **1**, 58. https://doi.org/10.1038/s43247-020-00059-6.

Píšková, A., Roman, M., Bulínová, M., Pokorný, M., Sanderson, D., Cresswell, A., Lirio, J. M., Coria, S. H., Nedbalová, L., Lami, A., Musazzi, S., Van de Vijver, B., Nývlt, D., & Kopalová, K. (2019). Late-Holocene palaeoenvironmental changes at Lake Esmeralda (Vega Island, Antarctic Peninsula) based on a multi-proxy analysis of laminated lake sediment. *The Holocene*, **29**(7), 1155–1175. https://doi.org/10.1177/0959683619838033.

Post, A. L., Galton-Fenzi, B. K., Riddle, M. J., Herraiz-Borreguero, L., O’Brien, P. E., Hemer, M. A., McMinn, A., Rasch, D., & Craven, M. (2014). Modern sedimentation, circulation and life beneath the Amery Ice Shelf, East Antarctica. *Continental Shelf Research*, **74**, 77–87. https://doi.org/10.1016/j.csr.2013.10.010.

Rathburn, A. E., Pichon, J.-J., Ayress, M. A., & De Deckker, P. (1997). Microfossil and stable-isotope evidence for changes in Late Holocene palaeoproductivity and palaeoceanographic conditions in the Prydz Bay region of Antarctica. *Palaeogeography, Palaeoclimatology, Palaeoecology*, **131**(3-4), 485-510. https://doi.org/10.1016/S0031-0182(97)00017-5.

Ritchie, P. A., Millar, C. D., Gibb, G. C., Baroni, C., & Lambert, D. M. (2004). Ancient DNA enables timing of the Pleistocene origin and Holocene expansion of two Adélie penguin lineages in Antarctica. *Molecular Biology and Evolution*, **21**, 240–248. https://doi.org/10.1093/molbev/msh012.

Roberts, D., & McMinn, A. (1999). A diatom-based palaeosalinity history of Ace Lake, Vestfold Hills, Antarctica. *The Holocene*, **9**(4), 401–408. https://doi.org/10.1191/095968399671725699.

Roberts, D., McMinn, A., Johnston, N., Gore, D., Melles, M., & Cremer, H. (2001a). An analysis of the limnology and sedimentary diatom flora of fourteen lakes and ponds from the Windmill Islands, East Antarctica. *Antarctic Science*, **13**(4), 410-419. https://doi.org/10.1017/S0954102001000578.

Roberts, D., McMinn, A., & Zwartz, D. (2000). An initial palaeosalinity history of Jaw Lake, Bunger Hills based on a diatom–salinity transfer function applied to sediment cores. *Antarctic Science*, **12**(2), 172-176. https://doi.org/10.1017/S0954102000000225.

Roberts, S., Monien, P., Foster, L. C., Loftfield, J., Hocking, E. P., Schnetger, B., Pearson, E. J, Juggins, S., Fretwell, P., Ireland, L., Ochyra, R., Haworth, A. R., Allen, C. S., Moreton, S. G., Davies, S. J., Brumsack, H.-J., Bentley, M. J., & Hodgson, D. A. (2017). Past penguin colony responses to explosive volcanism on the Antarctic Peninsula. *Nature communication*, **8**, 14914. https://doi.org/10.1038/ncomms14914.

Roberts, D., Roberts, J. L., Gibson, J. A. E., McMinn, A., & Heijnis, H. (1999). Palaeohydrological modelling of Ace Lake, Vestfold Hills, Antarctica. *The Holocene*, **9**(5), 515–520. https://doi.org/10.1191/095968399672424476.

Roberts, D., van Ommen, T. D., McMinn, A., Morgan, V., & Roberts, J. L. (2001b). Late-Holocene East Antarctic climate trends from ice-core and lake-sediment proxies. *The Holocene*, **11**(1), 117–120. https://doi.org/10.1191/095968301677143452.

Robinson, S.A., King, D.H., Bramley-Alves, J., Waterman, M. J., Ashcroft, M. B., Wasley, J., Turnbull, J. D., Miller, R. E., Ryan-Colton, E., Benny, T., Mullany, K., Clarke, L. J., Barry, L. A., & Hua, Q. (2018). Rapid change in East Antarctic terrestrial vegetation in response to regional drying. *Nature Climate Change*, **8**, 879–884. https://doi.org/10.1038/s41558-018-0280-0.

Román-González, A. (2021). Sclerochronology in the Southern Ocean. *Polar Biology*, **44**, 1485–1515. https://doi.org/10.1007/s00300-021-02899-0.

Román-González, A., Scourse, J. D., Butler, P. G., Reynolds, D. J., Richardson, C. A., Peck, L. S., Brey, T., & Hall, I. R. (2017). Analysis of ontogenetic growth trends in two marine Antarctic bivalves *Yoldia eightsi* and *Laternula elliptica*: Implications for sclerochronology. *Palaeogeography, Palaeoclimatology, Palaeoecology*, **465**, 300–306. https://doi.org/10.1016/j.palaeo.2016.05.004.

Royles, J., Amesbury, M. J., Convey, P., Griffiths, H., Hodgson, D. A., Leng, M. J., & Charman, D. J. (2013). Plant and soil microbes respond to recent warming on the Antarctic Peninsula. *Current Biology*, **23**(17), 1702–1706. https://doi.org/10.1016/j.cub.2013.07.011.

Royles, J., Ogée, J., Wingate, L., Hodgson, D. A., Convey, P., & Griffiths, H. (2012). Carbon isotope evidence for recent climate-related enhancement of CO2 assimilation and peat accumulation rates in Antarctica. *Global Change Biology*, **18**(10), 3112–3124. https://doi.org/10.1111/j.1365-2486.2012.02750.x/.

Rudd, R. C., Tyler, J. J., Tibby, J., Yokoyama, Y., Tavernier, I., Verleyen, E., Fukui, M., & Takano, Yl (2016). A diatom-inferred record of lake variability during the last 900 years in Lützow–Holm Bay, East Antarctica. *Journal of Quaternary Science*, **31**, 114-125. https://doi.org/10.1002/jqs.2845.

Saunders, K. M., Roberts, S. J., Perren, B., Butz, C., Sime, L., Davies, S., Nieuwenhuyze, W. V., Grosjean, M., & Hodgson, D. A. (2018). Holocene dynamics of the Southern Hemisphere westerly winds and possible links to CO2 outgassing. *Nature Geoscience*, **11**, 650–655. https://doi.org/10.1038/s41561-018-0186-5.

Schwab, M. (1998). Rekonstruktion der spätquartären Klima- und Umweltgeschichte der Schirmacher Oase und des Wohlthat Massivs (Ostantarktika) = Reconstruction of the late quaternary climatic and environmental history of the Schirmacher Oasis and the Wohlthat Massif (East Antarctica). Berichte zur Polarforschung (Reports on Polar Research). Bremerhaven: Alfred Wegener Institute for Polar and Marine Research, 293.

Sedwick, P. N., Harris, P. T., Robertson, L. G., McMurtry, G. N., Cremer, M. D., & Robinson, P. (2010). Holocene sediment records from the continental shelf of Mac. Robertson Land, East Antarctica. *Paleoceanography and Paleoclimatology*, **16**(2), 212–225. https://doi.org/10.1029/2000PA000504.

Selkirk, D. R., Selkirk, P. M., Bergstrom, D. M., & Adamson, D. A. (1988). Ridge top peats and palaeolake deposits on Macquarie Island. *Papers and Proceedings of the Royal Society of Tasmania*, **122**, 83–90.

Selkirk, D. R., Selkirk, P. M., & Griffin, K. (1983). Palynological evidence for Holocene environmental change and uplift on Wireless Hill, Macquarie Island. *Proceedings of the Linnean Society of New South Wales*, **107**, 1-17.

Shevenell, A. E., Ingalls, A. E., Domack, E. W., & Kelly, C. (2011). Holocene Southern Ocean surface temperature variability west of the Antarctic Peninsula. *Nature*, **470**, 250-254. https://doi.org/10.1038/nature09751.

Sjunneskog, C. & Taylor, F. (2002). Postglacial marine diatom record of the Palmer Deep, Antarctic Peninsula (ODP Leg 178, Site 1098) 1. Total diatom abundance, *Paleoceanography*, **17**(3), PAL 4-1–PAL 4-8. https://doi:10.1029/2000PA000563.

Smith, A. M. (2007). Age, growth and carbonate production by erect rigid bryozoans in Antarctica. *Palaeogeography, Palaeoclimatology, Palaeoecology*, **256**(1), 86–98. https://doi.org/10.1016/j.palaeo.2007.09.007.

Soler-Membrives, A., Linse, K., Miller, K. J., & Arango, C. P. (2017). Genetic signature of Last Glacial Maximum regional refugia in a circum-Antarctic sea spider. *Royal Society Open Science*, **4**(10), 170615. https://doi.org/10.1098/rsos.170615.

Squier, A. H., Hodgson, D. A., & Keely, B. J. (2002). Sedimentary pigments as markers for environmental change in an Antarctic lake. *Organic Geochemistry*, **33**(12),1655-1665. https://doi.org/10.1016/S0146-6380(02)00177-8.

Squyres, S. W., Andersen, D. W., Nedell, S. S., & Wharton, R. A. Jr. (1991). Lake Hoare, Antarctica: sedimentation through a thick perennial ice cover. *Sedimentology*, **38**, 363-379. https://doi.org/10.1111/j.1365-3091.1991.tb01265.x.

Stelling, J. J., Yu, Z., Loisel, J., & Beilman, D. W. (2018). Peatbank response to late Holocene temperature and hydroclimate changes in the western Antarctic Peninsula. *Quaternary Science Reviews*, **188**, 77–89. https://doi.org/10.1016/j.quascirev.2017.10.033.

Stickley, C. E., Pike, J., Leventer, A., Dunbar, R., Domack, E. W., Brachfeld, S., Manley, P., & McClennan, C. (2005). Deglacial ocean and climate seasonality in laminated diatom sediments, Mac.Robertson Shelf, Antarctica. *Palaeogeography, Palaeoclimatology, Palaeoecology*, **227**(4), 290–310. https://doi.org/10.1016/j.palaeo.2005.05.021.

Shevenell, A. E., Domack, E. W., & Kernan, G. (1996). Record of Holocene paleoclimate change along the Antarctic Peninsula: evidence from glacial marine sediments, Lallemand Fjord. In: Banks, M.R., & Brown, M.J., (Eds.), Climate succession and glacial record of the Southern Hemisphere (pp. 55–64). Proceedings of the Royal Society of Tasmania 130.

Stevens, M. I., & D’Haese, C. A. (2014). Islands in ice: isolated populations of *Cryptopygus sverdrupi* (Collembola) among nunataks in the Sør Rondane Mountains, Dronning Maud Land, Antarctica. *Biodiversity*, **15**(2-3), 169–177. https://doi.org/10.1080/14888386.2014.928791.

Strother, S. L., Salzmann, U., Roberts, S. J., Hodgson, D. A., Woodward, J., Nieuwenhuyze, W. V., Verleyen, E., Vyverman, W., & Moreton, S. G. (2015). Changes in Holocene climate and the intensity of Southern Hemisphere Westerly Winds based on a high-resolution palynological record from sub-Antarctic South Georgia. *The Holocene*, **25**(2), 263–279. https://doi.org/10.1177/0959683614557576.

Strugnell, J. M., Watts, P. C., Smith, P. J., & Allcock, A. L. (2012). Persistent genetic signatures of historic climatic events in an Antarctic octopus. *Molecular Ecology*, **21**(11), 2775–2787. https://doi.org/10.1111/j.1365-294X.2012.05572.x.

Sun, L., Xie, Z., & Zhao, J. (2000). A 3,000-year record of penguin populations. *Nature*, **407**, 858. https://doi.org/10.1038/35038163.

Tada, Y., Wada, H., & Miura, H. (2006). Seasonal stable oxygen isotope cycles in an Antarctic bivalve shell (*Laternula elliptica*): a quantitative archive of ice-melt runoff. *Antarctic Science*, **18**(1), 111–115. https://doi.org/10.1017/S0954102006000101.

Tang, Z., Shi, X., Zhang, X., Chen, Z., Chen, M.-T., Wang, X., Wang, H., Liu, H., Lohmann, G., Li, P., Ge, S., & Huang, Y. (2016). Deglacial biogenic opal peaks revealing enhanced Southern Ocean upwelling during the last 513 ka. *Quaternary International*, **425**, 445–452. https://doi.org/10.1016/j.quaint.2016.09.020.

Taylor, F., & Leventer, A. (2003). Late Quaternary palaeoenvironments in Prydz Bay, East Antarctica: Interpretations from marine diatoms. *Antarctic Science*, **15**(4), 512–521. https://doi.org/10.1017/S0954102003001639.

Taylor, F., & McMinn, A. (2002). Evidence from diatoms for Holocene climate fluctuation along the East Antarctic margin. *The Holocene*, **11**(4), 455–466. https://doi.org/10.1191/095968301678302896.

Taylor, F., & McMinn, A. (2002). Late Quaternary Diatom Assemblages from Prydz Bay, Eastern Antarctica. *Quaternary Research*, **51**(1), 151–161. https://doi.org/10.1006/qres.2001.2279.

Taylor, F., Whitehead, J., & Domack, E. (2001). Holocene paleoclimate change in the Antarctic Peninsula: evidence from the diatom, sedimentary and geochemical record. *Marine Micropaleontology*, **41**, 25-43. https://doi.org/10.1016/S0377-8398(00)00049-9.

Toro, M., Granados, I., Pla, S., Giralt, S., Antoniades, D., Galán, L., Cortizas, A. M., Lim, H. S., & Appleby, P. E. (2013). Chronostratigraphy of the sedimentary record of Limnopolar Lake, Byers Peninsula, Livingston Island, Antarctica. *Antarctic Science*, **25**(2), 198–212. https://doi.org/10.1017/S0954102012000788.

Trucchi, E., Gratton, P., Whittington, J. D., Cristofari, R., Le Maho, Y., Stenseth, N. C., & Le Bohec, C. (2014). King penguin demography since the last glaciation inferred from genome-wide data. *Proceedings of the Royal Society B: Biological Sciences*, **281**, 1787. https://doi.org/10.1098/rspb.2014.0528.

Turney, C. S. M., Jones, R. T., Fogwill, C., Hatton, J., Williams, A. N., Hogg, A., Thomas, Z. A., Palmer, J., Mooney, S., & Reimer, R. W. (2016). A 250-year periodicity in Southern Hemisphere westerly winds over the last 2600 years. *Climate of the Past*, **12**, 189–200. https://doi.org/10.5194/cp-12-189-2016, 2016.

Van der Putten, N., Hébrard, J.-P., Verbruggen, C., Van de Vijver, B., Disnar, J.-R., Spassov, S., Keravis, D., de Beaulieu, J.-L., De Dapper, M., Hus, J., Thouveny, N., & Frenot, Y. (2008). An integrated palaeoenvironmental investigation of a 6200 year old peat sequence from Île de la Possession, Îles Crozet, sub-Antarctica. *Palaeogeography, Palaeoclimatology, Palaeoecology*, **270**, 179e195. https://doi.org/10.1016/j.palaeo.2008.09.014.

Van der Putten, N., Verbruggen, C., Ochyra, R., de Beaulieu, J.-L., De Dapper, M., Spassov, S., Hus, J., & Thouveny, N. (2009a). Peat bank growth, Holocene palaeoecology and climate history of South Georgia (sub-Antarctica), based on a botanical macrofossil record. *Quaternary Science Reviews*, **28**(1-2), 65-79. https://doi.org/10.1016/j.quascirev.2008.09.023.

Van der Putten, N., Verbruggen, C., Björck, S., Michel, E., Disnar, J.-R., Chapron, E., Moine, B. N., & de Beaulieu, J. L. (2015). The Last Termination in the South Indian Ocean: A unique terrestrial record from Kerguelen Islands (49°S) situated within the Southern Hemisphere westerly belt. *Quaternary Science Reviews*, **122**, 142-157. https://doi.org/10.1016/j.quascirev.2015.05.010.

Van der Putten, N., Verbruggen, C., Ochyra, R., Spassov, S., de Beaulieu, J.-L., De Dapper, M., Hus, J., & Thouveny, N. (2009b) Peat bank growth, Holocene palaeoecology and climate history of South Georgia (sub-Antarctica), based on a botanical macrofossil record. *Quaternary Science Reviews*, **28**, 65-79. https://doi.org/10.1016/j.quascirev.2008.09.023.

Venturelli, R. A., Siegfried, M. R., Roush, K. A., Li, W., Burnett, J., Zook, R., Fricker, H. A., Priscu, J. C., Leventer, A., & Rosenheim, B. E. (2020). Mid-Holocene Grounding Line Retreat and Readvance at Whillans Ice Stream, West Antarctica. *Geophysical Research Letters*, **47**(15), e2020GL088476. https://doi.org/10.1029/2020GL088476.

Verkulich, S. R., Melles, M., Hubberten, H.-W. & Pushina, Z. V. (2002). Holocene environmental changes and development of Figurnoye Lake in the southern Bunger Hills, East Antarctica. *Journal of Paleolimnology*, **28**, 253–267. https://doi.org/10.1023/A:1021661700899.

Verleyen, E., Hodgson, D. A., Milne, G. A., Sabbe, K., & Vyverman, W. (2005). Relative sea level history from the Lambert Glacier Region (East Antarctica) and its relation to deglaciation and Holocene glacier re-advance. *Quaternary Research*, **63**(1), 45–52. https://doi.org/10.1016/j.yqres.2004.09.005.

Verleyen, E., Hodgson, D. A., Sabbe, K., Cremer, H., Emslie, S. D., Gibson, J., Hall, B., Imura, S., Kudoh, S., Marshall, G. J.,  McMinn, A., Melles, M., Newman, L., Roberts, D., Roberts, S. .J., Singh, S. M., Sterken, M., Tavernier, I., Verkulich, S., Van de Vyver, E., Nieuwenhuyze, W. V., Wagner, B., & Vyverman, W. (2011) Post-glacial regional climate variability along the East Antarctic coastal margin—Evidence from shallow marine and coastal terrestrial records. *Earth-Science Reviews*, **104**(4), 199-212. https://doi.org/10.1016/j.earscirev.2010.10.006.

Verleyen, E., Hodgson, D. A., Sabbe, K., Vanhoutte, K., & Vyverman, W. (2004). Coastal oceanographic conditions in the Prydz Bay region (East Antarctica) during the Holocene recorded in an isolation basin. *The Holocene*, **14**(2), 246–257. https://doi.org/10.1191/0959683604hl702rp.

Verleyen, E., Hodgson, D.A., Vyverman, W., Roberts, D., McMinn, A., Vanhoutte, K., & Sabbe, K. (2003). Modelling diatom responses to climate induced fluctuations in the moisture balance in continental Antarctic lakes. *Journal of Paleolimnology*, **30**, 195–215. https://doi.org/10.1023/A:1025570904093.

Vieira, R., Marotta, H., Kellem da Rosa, K., Jaña, R., Lorenz Simões, C., de Souza Júnior, E., Ferreira, F., Ronquette Santos, L., Victor dos Santos, J., Aurélio Perroni, M., Gonçalves, M., Farias Santos, J. P., Issa Rodrigues, R., Medeiros Galvão, J. C., & de Sá Felizardo, J. P. (2015). Análisis Sedimentológico y geomorfológico de áreas lacustres en la Península Fildes, Isla Rey Jorge, Antártica Marítima. *Investigaciones Geográficas*, **49**, 3–30. https://doi.org/10.5354/0719-5370.2015.37511.

Wasell, A., & Håkansson, H. (1992). Diatom stratigraphy in a lake on horseshoe island, antarctica: A marine-brackish-fresh water transition with comments on the systematics and ecology of the most common diatoms. *Diatom Research*, **7**(1), 157-194. https://doi.org/10.1080/0269249X.1992.9705205.

Wagner, B., Cremer, H., Hultzsch, N., Gore, D. B., & Melles, M. (2004). Late Pleistocene and Holocene history of Lake Terrasovoje, Amery Oasis, East Antarctica, and its climatic and environmental implications. *Journal of Paleolimnology*, **32**, 321–339. https://doi.org/10.1007/s10933-004-0143-8.

Wagner, B., Hultzsch, N., Melles, M., & Gore, D. (2007). Indications of Holocene sea-level rise in Beaver Lake, East Antarctica. *Antarctic Science*, **19**(1), 125–128. https://doi.org/10.1017/S095410200700017X.

Whitehead J. M., & Bohaty, S. M. (2003). Pliocene summer sea-surface temperature reconstruction using silicoflagellates from Southern Ocean ODP Site 1165. *Paleoceanography and Paleoclimatology*, **18**(3), 1–10. https://doi.org/10.1029/2002PA000829.

Whitehead, J. M., & Mckelvey, B. C. (2001). The stratigraphy of the Pliocene—lower Pleistocene Bardin Bluffs Formation, Amery Oasis, northern Prince Charles Mountains, Antarctica. *Antarctic Science*, **13**(1), 79–86. https://doi.org/10.1017/S0954102001000128.

Whitehead, J. M., Quilty, P. G., Mckelvey, B. C., & O’Brien, P. E. (2006). A review of the Cenozoic stratigraphy and glacial history of the Lambert Graben—Prydz Bay region, East Antarctica. *Antarctic Science*, **18**(1), 83–99.

Wilson, D. J., Bertram, R. A., Needham, E. F. van de Flierdt, T., Welsh, K., McKay, R. M., Mazumder, A., Riesselman, C. R., Jimenez-Espejo, F. J., & Escutia, C. (2018). Ice loss from the East Antarctic Ice Sheet during late Pleistocene interglacials. *Nature*, **561**, 383–386. https://doi.org/10.1038/s41586-018-0501-8.

Wilson, D. J., Struve, T., van de Flierdt, T., Chen, T., Li, T., Burke, A., & Robinson, L. F. (2020). Sea-ice control on deglacial lower cell circulation changes recorded by Drake Passage deep-sea corals. *Earth and Planetary Science Letters*, **544**, 116405. https://doi.org/10.1016/j.epsl.2020.116405.

Wing, S. R., O’Connell-Milne, S. A., Wing, L. C., & Reid, M. R. (2020). Trace metals in Antarctic clam shells record the chemical dynamics of changing sea ice conditions. *Limnology and Oceanography*, **65**(3), 504–514. https://doi.org/10.1002/lno.11318.

Wu, L., Wilson, D. J., Wang, R., Passchier, S., Krijgsman, W., Yu, X., Wen, T., Xiao, W., & Liu, Z. (2021). Late Quaternary dynamics of the Lambert Glacier-Amery Ice Shelf system, East Antarctica. *Quaternary Science Reviews*, **252**, 106738. https://doi.org/10.1016/j.quascirev.2020.106738.

Wu, L., Wang, R., Xian, W., Ge, S., Chen, Z., & Krijgsman, W. (2017). Productivity-climate coupling recorded in Pleistocene sediments off Prydz Bay (East Antarctica). *Palaeogeography, Palaeoclimatology, Palaeoecology*, **485**(1), 260–270. https://doi.org/10.1016/j.palaeo.2017.06.018.

Liu, X., Sun, L., Xie, Z., Yin, Z., & Wang, Y. (2005). A 1300-year record of penguin populations at Ardley Island in the Antarctic, as deduced from the geochemical data in the ornithogenic lake sediments. *Arctic, Antarctic, and Alpine Research*, **37**(4), 490-498. https://doi.org/10.1657/1523-0430(2005)037[0490:AYROPP]2.0.CO;2.

Xiao, W., Frederichs, T., Gersonde, R., Kuhn, G., Esper, O., & Zhang, X. (2016). Constraining the dating of late Quaternary marine sediment records from the Scotia Sea (Southern Ocean). *Quaternary Geochronology*, **31**, 97–118. https://doi.org/10.1016/j.quageo.2015.11.003.

Xu, Q. B., Gao, Y. S., Yang, L. J., Yang, W. Q., Chu, Z. D., Wang, Y. H., Sun, L. G., & Xie, Z. Q. (2020). Abandonment of penguin subcolonies in the late nineteenth century on inexpressible island, Antarctica. *Journal of Geophysical Research: Biogeosciences*, **125**(12), e2020JG006080. https://doi.org/10.1029/2020jg006080.

Yeloff, D., Mauquoy, D., Barber, K., Way, S., van Geel, B., & Turney, C. S. M. (2007). Volcanic ash deposition and long-term vegetation change on Subantarctic Marion Island. *Arctic. Antarctic and Alpine Research*, **39**(3), 500-511. https://doi.org/10.1657/1523-0430(06-040)[YELOFF]2.0.CO;2.

Younger, J. L., Emmerson, L. M., & Miller, K. J. (2015a). The influence of historical climate changes on Southern Ocean marine predator populations: a comparative analysis. *Global Change Biology*, **22**(2), 474-493. https://doi.org/10.1111/gcb.13104.

Younger, J. L., Emmerson, L., Southwell, C., Lelliott, P., & Miller, K. (2015b) Proliferation of East Antarctic Adélie penguins in response to historical deglaciation. *BMC Evolutionary Biology*, **15**, 236. https://doi.org/10.1186/s12862-015-0502-2.

Younger, J. L., Clucas, G. V., Kooyman, G., Wienecke, B., Rogers, A. D., Trathan, P. N., Hart, T., & Miller, K. J. (2015c), Too much of a good thing: sea ice extent may have forced emperor penguins into refugia during the last glacial maximum. *Global Change Biology*, **21**, 2215-2226. https://doi.org/10.1111/gcb.12882.

Younger, J.L., van den Hoff, J., Wienecke, B., Hindell, M., & Miller, K. J. (2016). Contrasting responses to a climate regime change by sympatric, ice-dependent predators. *BMC Evolutionary Biology*, **16**, 61. https://doi.org/10.1186/s12862-016-0630-3.

Yu, Z., Beilman, D. W., & Loisel, J. (2016). Transformations of landscape and peat-forming ecosystems in response to late Holocene climate change in the western Antarctic Peninsula. Geophysical Research Letters, **43**(13), 7186–7195, https://doi.org/10.1002/2016GL069380.

Zale, R. (1993). Lake sediments around the Antarctic Peninsula: archives of climatic and environmental changes. PhD thesis, University of Umeå.

Zale, R., & Karlen, W. (1989). Lake sediment cores from the Antarctic Peninsula and surrounding islands. *Geografiska Annaler: Series A, Physical Geography*, **71**, 211-220. https://doi.org/10.1080/04353676.1989.11880288.

Zwartz, D. P., Bird, M., Stone, J., & Lambeck, K. (1998a). Holocene sea-level change and ice-sheet history in the Vestfold Hills, East Antarctica. *Earth and Planetary Science Letters*, **155**(1-2), 131–145. https://doi.org/10.1016/S0012-821X(97)00204-5.

Zwartz, D. P., Miura, H., Takada, M., & Moriwaki, K. (1998b). Holocene lake sediments and sea-level change at Mt. Riiser-Larsen. *Polar Geoscience*, **11**, 249-259.
